# Regioselective Hydroboration of Terminal Alkenes with the Addition of Triflic Acid to Borane

**DOI:** 10.1021/acs.joc.5c03231

**Published:** 2026-04-30

**Authors:** Ilias Fourati, K. A. Woerpel

**Affiliations:** Department of Chemistry, 5894 New York University, 100 Washington Square East, New York, New York 10003, United States

## Abstract

The preactivation of borane dimethyl sulfide complex with triflic acid formed a highly reactive hydroborating agent, H_2_BOTf. This reagent, generated in situ, reacted with terminal alkenes with higher regioselectivity than for borane dimethyl sulfide complex, favoring the primary alcohol products with regioisomeric ratios of up to 99:1. Oxidation of boranes could be performed with *t*-BuOOH and CsOH·H_2_O, which, in the case of hindered boranes, was faster and higher-yielding than reactions using standard oxidation conditions (H_2_O_2_ and NaOH).

## Graphical abstract

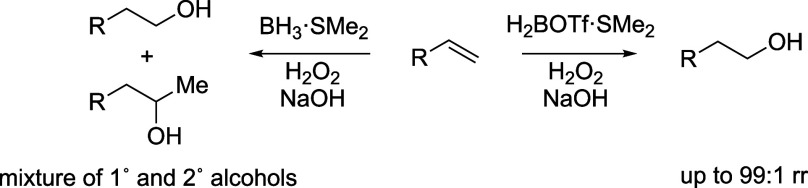

## Introduction

The formation of alcohols from alkenes by hydroboration-oxidation is a widely used process in synthetic organic chemistry.^1^ Despite its synthetic utility, hydroboration-oxidation can suffer from some complications. The regioselectivity of hydroboration-oxidation reactions of monosubstituted alkenes with simple boranes is often high, but the secondary alcohol product is usually formed as an impurity.^2–4^ The regioselectivity can be improved by the use of sterically hindered boranes such as 9-borabicyclononane (9-BBN).^5^ Metal-catalyzed hydroborations have also emerged as methods to address reactivity and regioselectivity.^6,7^ Once hydroboration is completed, oxidation to form the alcohol product can be slow, particularly for sterically hindered alkenes.^8–10^ In this note, we demonstrate that the pretreatment of borane dimethyl sulfide complex (BH_3_·SMe_2_) with triflic acid (TfOH) forms a reagent that undergoes hydroboration with higher regioselectivity than the corresponding reaction with BH_3_·SMe_2_. We also demonstrate that the oxidation of boranes with *tert*-butyl hydroperoxide (*t*-BuOOH) and cesium hydroxide hydrate (CsOH·H_2_O) in polar aprotic solvent is faster than the oxidation under standard conditions (H_2_O_2_ and NaOH).

## Results and Discussion

Initial experiments to address the regioselectivity of the hydroboration reaction were conducted because we needed a synthesis of primary alcohols that did not contain any secondary alcohols as impurities. Although a number of variants of the reaction have been reported, such as the use of H_2_BCl·dioxane,^11^ we found that BH_3_·SMe_2_ was an easier borane to handle and it was more economical. Initial experiments indicated that an effective reagent could be generated by mixing BH_3_·SMe_2_ with either a Lewis or Brønsted acid before introduction of the alkene. Among the reagents examined were boron trifluoride etherate, trimethylsilyl trifluoromethanesulfonate, trityl tetrafluoroborate,^12^ camphorsulfonic acid, copper triflate, and scandium triflate. The use of none of these additives improved regioselectivity.

Optimization studies revealed that addition of TfOH to BH_3_·SMe_2_ ^13^ significantly improved regioselectivity, favoring formation of the primary alcohol product compared to using BH_3_·SMe_2_ without pretreatment. Further optimization studies showed that a reaction temperature of −25 °C led to the highest regioselectivity for the hydroboration step (Table 1), where the ratios of products reported were determined by single-pulse NMR spectroscopy, a method that has been used to determine ratios of products accurately provided that samples are concentrated.^14^ These ratios were confirmed by quantitative ^13^C­{^1^H} NMR spectroscopy.^15^ By contrast, the regioselectivity of hydroboration with BH_3_·SMe_2_ did not vary with temperature. Preactivation of BH_3_·SMe_2_ at −78 °C prior to the addition of alkene was most effective. The formation of gas bubbles (presumably H_2_ gas) was observed if TfOH were added to BH_3_·SMe_2_ at higher temperatures. To avoid formation of frothy mixtures, the addition was performed at −78 °C. The use of TfOH with borane-THF complex did not change the regioselectivity (84:16). Although regioselectivities of hydroboration with TfOH and BH_3_·SMe_2_ in hexanes, Et_2_O, and DMF were similar, the use of CH_2_Cl_2_ was most convenient experimentally.

**Table 1:**
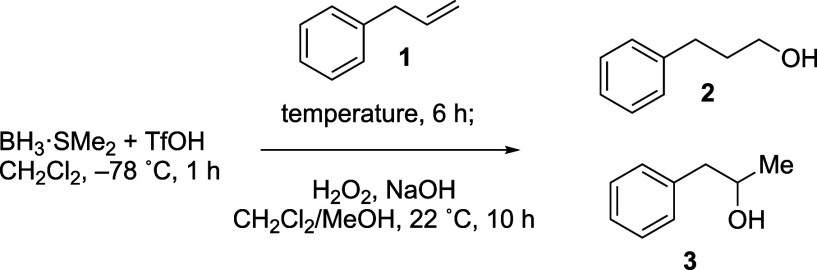
Optimization of the Temperature of the Reaction

entry	temperature	**2:3** ^a^	**2:3** ^a^ (without TfOH)
1	22 °C	90:10	86:14
2	0 °C	94:6	86:14
3	–25 °C	99:1	86:14

a Determined by single-pulse ^1^H NMR spectroscopy^14^ and inverse-gated ^13^C­{^1^H} spectroscopy.^15^

Control experiments suggested that the combination of BH_3_·SMe_2_ and TfOH formed H_2_BOTf and H_2_ gas.^13^ The ^1^H NMR spectrum of a mixture of BH_3_·SMe_2_ after addition of TfOH in toluene-*d*_8_ showed a singlet at δ 4.62 ppm, closely matching the reported peak of H_2_(*g*) in deuterated toluene (δ 4.55 ppm).^16^ The ^1^H NMR spectrum also revealed that dimethyl sulfide (Me_2_S) was present and likely complexed to the borane species in the resting state of the reagent. This conclusion is supported by an experiment where the addition of exogenous Me_2_S (4 equiv) almost completely suppressed the reaction.^17^ The ^11^B NMR spectrum showed that, upon addition of TfOH, the signal corresponding to BH_3_·SMe_2_ (δ −20 ppm) disappeared and the major product (about 90% of the mixture) appeared as a triplet at δ −2.0 ppm (*J* = 128 Hz). This peak is characteristic of the chemical shifts reported for boryl triflates.^13,18^ A smaller triplet was observed at δ −8.7 ppm, which is likely another H_2_BX·SMe_2_ species that could react similarly.

The steric and electronic properties of the electron-deficient borane H_2_BOTf likely enhance its preference for addition to the less substituted carbon atom of terminal alkenes.^19^ Sterically hindered borane reagents such as 9-BBN^20^ and thexylchloroborane^21^ undergo hydroboration with alkenes with high regioselectivity, and the improved selectivity of H_2_BOTf may similarly coincide with the added steric hindrance at the borane center. The electronic properties of H_2_BOTf could also play a role in the increased regioselectivity by making the boron atom more electrophilic, which likely also explains the regioselective hydroboration by chloroboranes.^11^ The origin of the regioselectivity of hydroboration, however, cannot be analyzed so simply.^22^

The optimized reaction conditions were employed to form primary alcohols from several terminal alkenes bearing electron-donating and electron-withdrawing groups (Table 2). The regioselectivity of the reaction was generally higher with the use of BH_3_·SMe_2_ and TfOH than for reactions where TfOH was omitted. This table also includes regioselectivities of hydroborations that were reported previously.^23–25^ Although the ratios are not identical, they are similar considering the differences in reagents, solvents, and reaction temperatures. The new values reported here are for experiments performed under comparable conditions to facilitate comparisons. The results shown in entries 1-4 show that oxygen atoms are not involved in directing the regioselectivity.^13^ The regioselectivity of hydroboration in the case of 2-allylphenol (**6**) is unlikely to be influenced by the presence of the hydroxyl group considering that 2-vinylphenol (**8**) reacted with the same regioselectivity and that the unsubstituted versions of these alkenes (**1** and **4**) reacted with similar regioselectivities with both reagents (as illustrated by comparing entries 1 and 2 to entries 3 and 4).

**Table 2:**
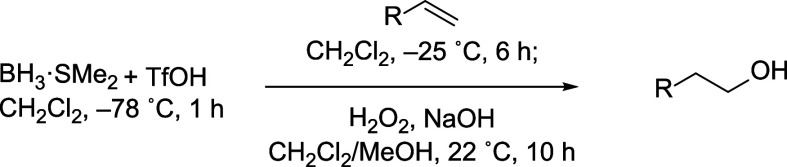

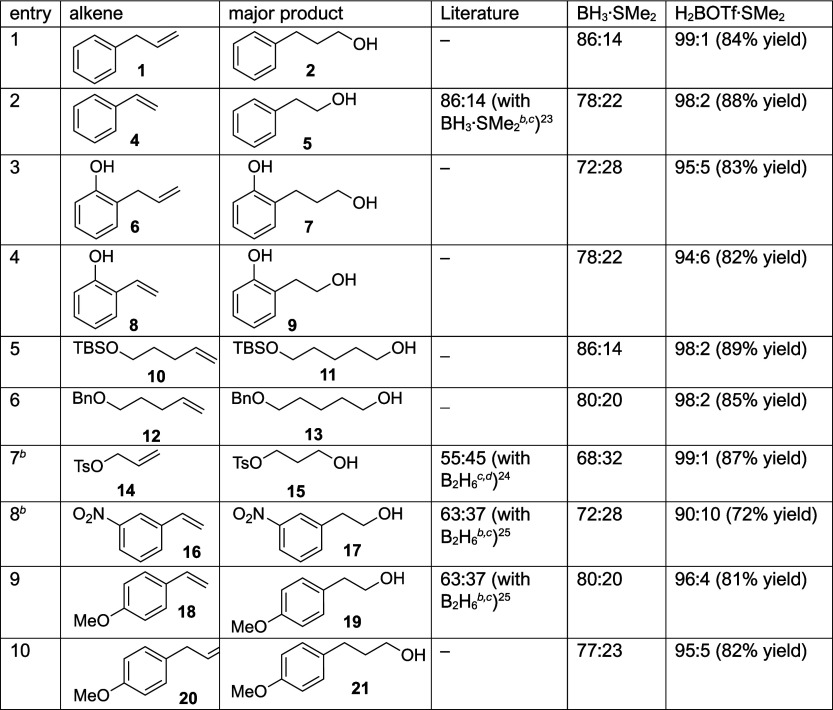
Regioisomeric Ratios of Primary to Secondary Alcohols^a^

a Determined by single-pulse ^1^H NMR spectroscopy^14^ and inverse-gated ^13^C­{^1^H} spectroscopy^15^ unless otherwise indicated.

b Reaction temperature was 20–22 °C.

c Regioselectivity was determined by gas chromatography.

d Reaction temperature was 0–5 °C.

The hydroboration of allyl tosylate (**14**) illustrates another advantage of the use of H_2_BOTf (Table 2, entry 8). This substrate was reported to undergo hydroboration more slowly than other alkenes because of the presence of the electron-withdrawing group, and it was also shown to react with low regioselectivity.^24^ By contrast, the hydroboration of allyl tosylate with H_2_BOTf produced the primary alcohol with only trace amounts of the secondary alcohol and none of the elimination products observed with other boranes.

The modified reaction conditions were also tested on internal alkenes (Table 3). Regioselectivity was high when BH_3_·SMe_2_ was used alone, but regioselectivity was at least as high using H_2_BOTf. The defluorination of a difluoroalkene^26^ could also be achieved in high yields (Table 3, entry 3). The regioselectivity was similar to the regioselectivity reported (entry 2).^27^

**Table 3:**
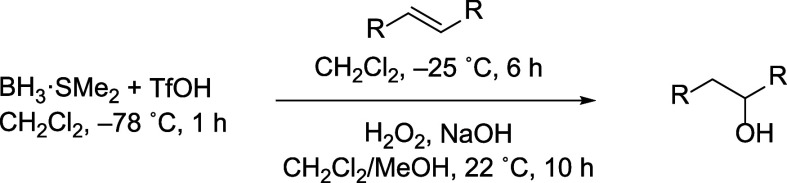

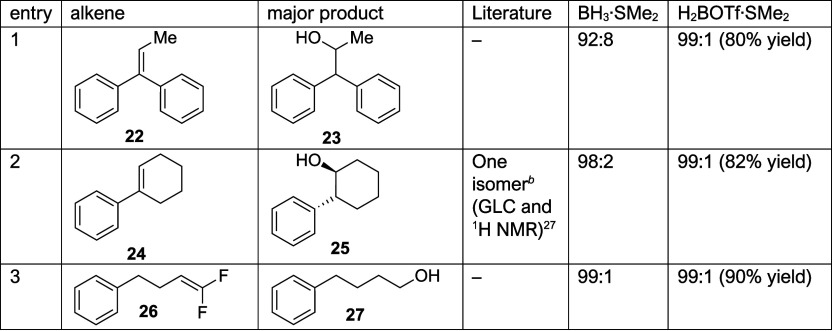
Regioisomeric Ratios of the Hydroboration of Internal Alkenes^a^

a Determined by single-pulse ^1^H NMR spectroscopy^14^ and inverse-gated ^13^C­{^1^H} spectroscopy^15^ unless otherwise indicated.

b Reaction temperature was 0–5 °C and regioselectivity was determined by gas chromatography and ^1^H NMR spectroscopy.

Another issue that we wanted to optimize was the oxidation of hindered boranes. Given that oxidations of boron–carbon bonds proceed through mechanisms analogous to those for the oxidations of silicon–carbon bonds,^28,29^ we explored the use of *t-*BuOOH and CsOH·H_2_O in a polar aprotic solvent.^30^ (−)-β-Pinene (**28**) was reported to undergo oxidation with H_2_O_2_/NaOH overnight following hydroboration to afford the corresponding alcohol in 91% yield.^31^ We repeated this experiment and found that, after exposure to the oxidation conditions for 1 h, (−)-*cis*-myrtanol (**29**) was formed in only 30% yield (Scheme 1). By contrast, oxidation of the borane using *t-*BuOOH and CsOH·H_2_O for 1 h gave 60% yield of **29**. In the case of nonhindered boranes derived from monosubstituted alkenes (Table 2), however, no appreciable difference in yields nor in reaction times were noted with the two oxidation conditions.^25^

**Scheme 1:**
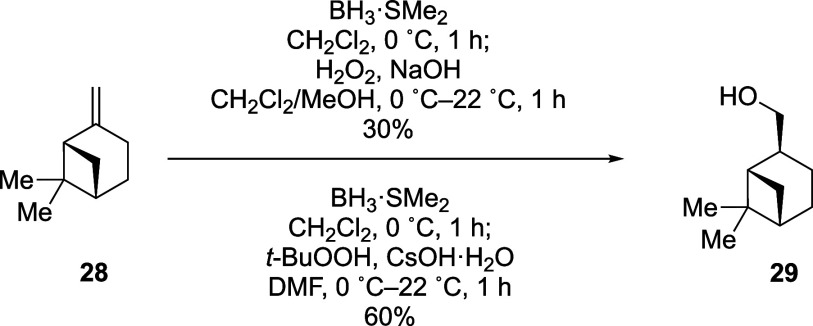
Comparison of Yields of Different Oxidation Reactions

## Conclusion

In conclusion, the regioselectivity of hydroboration of monosubstituted alkenes can be improved by using a mixture of BH_3_·SMe_2_ and TfOH. After oxidation, the primary alcohol product could be formed with up to 99:1 regioselectivity. The use of *t-*BuOOH and CsOH·H_2_O to oxidize a borane following hydroboration occurred more rapidly than oxidation under standard reaction conditions (H_2_O_2_/NaOH).

## Experimental Section

### General Methods

^1^H NMR and ^13^C­{^1^H} NMR spectra were obtained at room temperature using Bruker AVIII-400 (400 and 100 MHz, respectively), AVIIIHD-400 (400 and 100 MHz, respectively), and Avance NEO-500 spectrometers (500 and 125 MHz, respectively). ^11^B NMR spectra were obtained at room temperature using an Avance NEO-500 (160 MHz) spectrometer. All spectroscopic data are reported as follows: chemical shifts are reported in ppm on the δ scale, ^1^H and ^13^C­{^1^H} NMR spectra are internally referenced to residual solvent (^1^H NMR: CDCl_3_, δ 7.26; ^13^C­{^1^H} NMR: CDCl_3_, δ 77.16), ^11^B­{^1^H} spectra are externally referenced to boron trifluoride etherate (^11^B NMR: δ 0.00), multiplicity (br = broad, s = singlet, d = doublet, t = triplet, q = quartet, quint = quintet, m = multiplet), coupling constants (Hz), and integration. Ratios of products were derived from one-pulse ^1^H NMR spectra and confirmed with ^13^C­{^1^H} NMR integrations^15^ using diagnostic peaks in the unpurified reaction mixture and HPLC analysis. Multiplicities of carbon peaks were defined using HSQC or ^13^C DEPT experiments. High-resolution mass spectra were acquired on an Agilent 6224 Accurate-Mass time-of-flight spectrometer and were obtained using peak matching. The ionization source used was electrospray ionization (ESI). Liquid chromatography was performed using forced flow (flash chromatography) of the indicated solvent system on silica gel (SiO_2_) 60 (230–400 mesh). Dichloromethane and dimethylformamide were dried and degassed using a solvent purification system before use. All dry reactions were run under a nitrogen atmosphere in glassware that had been flame-dried under reduced pressure. Unless otherwise noted, all reagents and substrates were commercially available.

### General Procedure for the Synthesis of Primary Alcohols with BH_3_·SMe_2_ and TfOH

To a cooled (−78 °C) solution of BH_3_·SMe_2_ (0.300 mL, 3.03 mmol, 3 equiv) in CH_2_Cl_2_ (16.0 mL, 0.19 M) was added TfOH (0.270 mL, 3.04 mmol, 3 equiv) dropwise. After 1 h, the solution was warmed to −25 °C and the alkene (1.00 mmol, 1 equiv) in CH_2_Cl_2_ (5.00 mL, 0.20 M) was added dropwise. After 6 h, 30% H_2_O_2_ (0.400 mL, 3.5 mmol, 3 equiv), 2.5 M aqueous NaOH (0.600 mL, 1.5 mmol, 1.5 equiv), and MeOH (4.0 mL, 0.25 M) were added. The reaction mixture was allowed to warm to room temperature. After 10 h, the reaction mixture was concentrated in vacuo to remove MeOH. The reaction mixture was diluted with brine (5.0 mL) and the aqueous layer was extracted with Et_2_O (3 × 20 mL). The combined organic phases were dried over anhydrous MgSO_4_, filtered, and concentrated in vacuo. Product ratios of the crude residues were determined by integrating single-pulse ^1^H NMR spectra using diagnostic peaks in the unpurified reaction mixture. The ratios were confirmed using ^13^C­{^1^H} NMR integrations of inverse gated spectra and HPLC analysis.

### General Procedure for the Synthesis of Primary Alcohols with BH_3_·SMe_2_

To a cooled (−25 °C) solution of BH_3_·SMe_2_ (0.300 mL, 3.03 mmol, 3 equiv) in CH_2_Cl_2_ (16.0 mL, 0.19 M) was added alkene (1.00 mmol, 1 equiv) in CH_2_Cl_2_ (5.00 mL, 0.20 M) dropwise. After 6 h, 30% H_2_O_2_ (0.400 mL, 3.5 mmol, 3.5 equiv), 2.5 M aqueous NaOH (0.600 mL, 1.5 mmol, 1.5 equiv), and MeOH (4.0 mL, 0.25 M) were added. The reaction mixture was allowed to warm to room temperature. After 10 h, the reaction mixture was concentrated in vacuo to remove MeOH. The reaction mixture was diluted with brine (5.0 mL) and the aqueous layer was extracted with Et_2_O (3 × 20 mL). The combined organic phases were dried over anhydrous MgSO_4_, filtered, and concentrated in vacuo. Product ratios of the crude residues were determined by integrating single-pulse ^1^H NMR spectra using diagnostic peaks in the unpurified reaction mixture. The ratios were confirmed using ^13^C­{^1^H} NMR integrations of inverse gated spectra and HPLC analysis.

### 3-Phenylpropan-1-ol **(2)**

The general procedure for the synthesis of primary alcohols with BH_3_·SMe_2_ and TfOH was followed. ^1^H NMR and ^13^C­{^1^H} NMR spectroscopic, and HPLC chromatographic analysis of the unpurified reaction mixture revealed that primary and secondary alcohols were formed as a 99:1 mixture of regioisomers. Purification by flash column chromatography (20:80 EtOAc/hexanes) afforded the major regioisomer **2** as a clear oil (0.114 g, 84%). The spectroscopic data are consistent with the data reported in the literature:^32^ ^1^H NMR (400 MHz, CDCl_3_) δ 7.31–7.28 (m, 2H), 7.22–7.20 (m, 3H), 3.68 (t, *J* = 6.4 Hz, 2H), 2.72 (t, *J* = 7.6 Hz, 2H), 1.94–1.87 (m, 2H); ^13^C­{^1^H} NMR (100 MHz, CDCl_3_) δ 141.8 (C), 128.45 (CH), 128.42 (CH), 125.8 (CH), 62.2 (CH_2_), 34.2 (CH_2_), 32.0 (CH_2_); HRMS (ESI-TOF) *m*/*z*: [M + H]^+^ Calcd for C_9_H_13_O, 137.0961; Found, 137.0957.

### 2-Phenylethan-1-ol **(5)**

The general procedure for the synthesis of primary alcohols with BH_3_·SMe_2_ and TfOH was followed. ^1^H NMR and ^13^C­{^1^H} NMR spectroscopic, and HPLC chromatographic analysis of the unpurified reaction mixture revealed that primary and secondary alcohols were formed as a 98:2 mixture of regioisomers. Purification by flash column chromatography (20:80 EtOAc/hexanes) afforded the major regiosiomer **5** as a clear oil (0.108 g, 88%). The spectroscopic data are consistent with the data reported in the literature:^33^ ^1^H NMR (400 MHz, CDCl_3_) δ 7.37–7.35 (m, 2H), 7.28–7.25 (m, 3H), 3.86 (t, *J* = 6.6 Hz, 2H), 2.89 (t, *J* = 6.6 Hz, 2H), 1.63 (s, 1H); ^13^C­{^1^H} NMR (100 MHz, CDCl_3_) δ 138.5 (C), 129.0 (CH), 128.5 (CH), 126.4 (CH), 63.6 (CH_2_), 39.2 (CH_2_); HRMS (ESI-TOF) *m*/*z*: [M + H]^+^ Calcd for C_8_H_11_O, 123.0804; Found, 123.0805.

### 2-(3-Hydroxypropyl)­phenol **(7)**

The general procedure for the synthesis of primary alcohols with BH_3_·SMe_2_ and TfOH was followed. ^1^H NMR and ^13^C­{^1^H} NMR spectroscopic, and HPLC chromatographic analysis of the unpurified reaction mixture revealed that primary and secondary alcohols were formed as a 95:5 mixture of regioisomers. Purification by flash column chromatography (20:80 EtOAc/hexanes) afforded the major regioisomer **7** as a yellow oil (0.126 g, 83%). The spectroscopic data are consistent with the data reported in the literature:^34^ ^1^H NMR (400 MHz, CDCl_3_) δ 7.11–7.07 (m, 2H), 6.88–6.82 (m, 2H), 3.64 (t, *J* = 5.9 Hz, 2H), 2.77 (t, *J* = 6.9 Hz, 2H), 1.87 (quint, *J* = 6.0 Hz, 2H); ^13^C­{^1^H} NMR (100 MHz, CDCl_3_) δ 154.4 (C), 130.6 (CH), 127.5 (C), 127.3 (CH), 120.8 (CH), 116.0 (CH), 60.8 (CH_2_), 32.2 (CH_2_), 25.2 (CH_2_); HRMS (ESI-TOF) *m*/*z*: [M* – H_2_O]^+^ Calcd for C_9_H_10_O, 134.0726; Found, 134.0723.

### 2-(2-Hydroxyethyl)­phenol **(9)**

The general procedure for the synthesis of primary alcohols with BH_3_·SMe_2_ and TfOH was followed. ^1^H NMR and ^13^C­{^1^H} NMR spectroscopic, and HPLC chromatographic analysis of the unpurified reaction mixture revealed that primary and secondary alcohols were formed as a 94:6 mixture of regioisomers. Purification by flash column chromatography (20:80 EtOAc/hexanes) afforded the major regioisomer **9** as a clear oil (0.113 g, 82% yield). The spectroscopic data are consistent with the data reported in the literature:^35^ ^1^H NMR (400 MHz, CDCl_3_) δ 7.16–7.11 (dt, 1H), 7.08–7.04 (dd, *J* = 7.8, 1.6 Hz), 6.89–6.83 (m, 2H), 3.92, (t, *J* = 5.5 Hz, 2H), 2.87 (t, *J* = 5.4 Hz, 2H); ^13^C­{^1^H} NMR (100 MHz, CDCl_3_) δ 155.1 (C), 131.1 (CH), 128.3 (CH), 126.7 (C), 120.7 (CH), 116.8 (CH), 64.4 (CH_2_), 34.6 (CH_2_); HRMS (ESI-TOF) *m*/*z*: [(M + H) −H_2_O]^+^ Calcd for C_8_H_9_O, 121.0654; Found, 121.0657.

### 5-((*tert*-Butyldimethylsilyl)­oxy)­pentan-1-ol **(11)**

The general procedure for the synthesis of primary alcohols with BH_3_·SMe_2_ and TfOH was followed. ^1^H NMR and ^13^C­{^1^H} NMR spectroscopic, and HPLC chromatographic analysis of the unpurified reaction mixture revealed that primary and secondary alcohols were formed as a 98:2 mixture of regioisomers Purification by flash column chromatography (20:80 EtOAc/hexanes) afforded the major regiosiomer **11** as a clear oil (0.194 g, 89%). The spectroscopic data are consistent with the data reported in the literature:^36^ ^1^H NMR (400 MHz, CDCl_3_) δ 3.65–3.59 (m, 4H), 1.61–1.51 (m, 5H), 1.43–1.37 (m, 2H), 0.88 (s, 9H), 0.04 (s, 6H); ^13^C­{^1^H} NMR (100 MHz, CDCl_3_) δ 63.2 (CH_2_), 63.0 (CH_2_), 32.6 (CH_2_), 26.1 (CH_3_), 22.1 (CH_2_), 18.5 (CH_2_), −5.1 (CH_3_); HRMS (ESI-TOF) *m*/*z*: [(M + H) −H_2_O]^+^ Calcd for C_11_H_25_OSi, 201.1669; Found, 201.1672.

### 5-(Benzyloxy)­pentan-1-ol **(13)**

The general procedure for the synthesis of primary alcohols with BH_3_·SMe_2_ and TfOH was followed. ^1^H NMR and ^13^C­{^1^H} NMR spectroscopic, and HPLC chromatographic analysis of the unpurified reaction mixture revealed that primary and secondary alcohols were formed as a 98:2 mixture of regioisomers. Purification by flash column chromatography (20:80 EtOAc/hexanes) afforded the major regiosiomer **13** as a clear oil (0.165 g, 85%). The spectroscopic data are consistent with the data reported in the literature:^37^ ^1^H NMR (400 MHz, CDCl_3_) δ 7.27–7.19 (m, 5H), 4.43 (s, 2H), 3.57 (t, *J* = 6.5 Hz, 2H), 3.41 (t, *J* = 6.5 Hz, 2H), 2.10 (s, 1H), 1.61–1.48 (m, 4H), 1.42–1.34 (m, 2H); ^13^C­{^1^H} NMR (100 MHz, CDCl_3_) δ 138.5 (C), 128.3 (CH), 127.6 (CH), 127.5 (CH), 72.9 (CH_2_), 70.2 (CH_2_), 62.8 (CH_2_), 32.5 (CH_2_), 29.4 (CH_2_), 22.4 (CH_2_); HRMS (ESI-TOF) *m*/*z*: [M + Na]^+^ Calcd for C_12_H_18_NaO_2_, 217.1199; Found, 217.1209.

### 3-Hydroxypropyl 4-Methylbenzenesulfonate **(15)**

The general procedure for the synthesis of primary alcohols with BH_3_·SMe_2_ and TfOH was followed. ^1^H NMR and ^13^C­{^1^H} NMR spectroscopic, and HPLC chromatographic analysis of the unpurified reaction mixture revealed that primary and secondary alcohols were formed as a 99:1 mixture of regioisomers. Purification by flash column chromatography (40:60 EtOAc/hexanes) afforded the major regioisomer **15** as a clear oil (0.200 g, 87%). The spectroscopic data are consistent with the data reported in the literature:^38^ ^1^H NMR (400 MHz, CDCl_3_) δ 7.82 (d, *J* = 8.8 Hz, 2H), 7.39 (d, *J* = 8.5 Hz, 2H), 4.18 (t, *J* = 6.1 Hz, 2H), 3.72 (t, *J* = 6.0 Hz, 2H), 2.45 (s, 3H), 1.89 (quint, *J* = 6.0 Hz, 2H); ^13^C­{^1^H} NMR (100 MHz, CDCl_3_) δ 145.0, 133.1, 130.0, 128.0, 67.5, 58.5, 31.7, 21.7; HRMS (ESI-TOF) *m*/*z*: [M + Na]^+^ Calcd for C_10_H_14_O_4_NaS, 253.0506; Found, 253.0510.

### 2-(3-Nitrophenyl)­ethan-1-ol **(17)**

The general procedure for the synthesis of primary alcohols with BH_3_·SMe_2_ and TfOH was followed, but hydroboration was warmed from −25 °C to 22 °C due to poor conversion of starting material, as determined by TLC. ^1^H NMR and ^13^C­{^1^H} NMR spectroscopic, and HPLC chromatographic analysis of the unpurified reaction mixture revealed that primary and secondary alcohols were formed as a 90:10 mixture of regioisomers. Purification by flash column chromatography (20:80 EtOAc/hexanes) afforded the major regioisomer **17** as a clear oil (0.120 g, 72%). The spectroscopic data are consistent with the data reported in the literature:^18^ ^1^H NMR (400 MHz, CDCl_3_) δ 8.10–8.02 (m, 2H), 7.58–7.52 (m, 2H), 3.92 (t, *J* = 6.5 Hz, 2H), 2.97 (t, *J* = 6.5 Hz, 2H), 1.70 (s, 1H); ^13^C­{^1^H} NMR (100 MHz, CDCl_3_) δ 148.3 (C), 140.9 (C), 135.4 (CH), 129.3 (CH), 123.8 (CH), 121.5 (CH), 62.9 (CH_2_), 38.5 (CH_2_); HRMS (ESI-TOF) *m*/*z*: [M + Na]^+^ Calcd for C_8_H_9_NNaO_3_, 190.0475; Found, 190.0468.

### 2-(4-Methoxyphenyl)­ethan-1-ol **(19)**

The general procedure for the synthesis of primary alcohols with BH_3_·SMe_2_ and TfOH was followed. ^1^H NMR and ^13^C­{^1^H} NMR spectroscopic, and HPLC chromatographic analysis of the unpurified reaction mixture revealed that primary and secondary alcohols were formed as a 96:4 mixture of regioisomers. Purification by flash column chromatography (20:80 EtOAc/hexanes) afforded the major regioisomer **19** as a yellow oil (0.123 g, 81%). The spectroscopic data are consistent with the data reported in the literature:^33^ ^1^H NMR (400 MHz, CDCl_3_) δ 7.16 (d, *J* = 8.6 Hz, 2H), 6.88 (d, *J* = 8.6 Hz, 2H), 3.84–3.80 (m, 5H), 2.81 (t, *J* = 6.7 Hz, 2H), 1.76 (s, 1H); ^13^C­{^1^H} NMR (100 MHz, CDCl_3_) δ 158.2 (C), 130.5 (C), 129.9 (CH), 114.0 (CH), 63.8 (CH_2_), 55.2 (CH_3_), 38.2 (CH_2_); HRMS (ESI-TOF) *m*/*z*: [(M + H) −H_2_O]^+^ Calcd for C_9_H_11_O, 135.0810; Found, 135.0813.

### 3-(4-Methoxyphenyl)­propan-1-ol **(21)**

The general procedure for the synthesis of primary alcohols with BH_3_·SMe_2_ and TfOH was followed. ^1^H NMR and ^13^C­{^1^H} NMR spectroscopic, and HPLC chromatographic analysis of the unpurified reaction mixture revealed that primary and secondary alcohols were formed as a 95:5 mixture of regioisomers. Purification by flash column chromatography (20:80 EtOAc/hexanes) afforded the major regioisomer **21** as a clear oil (0.136 g, 82%). The spectroscopic data are consistent with the data reported in the literature:^39^ ^1^H NMR (400 MHz, CDCl_3_) δ 7.05 (d, *J* = 8.6 Hz, 2H), 6.76 (d, *J* = 8.6 Hz, 2H), 3.71 (s, 3H), 3.59 (t, *J* = 6.4 Hz, 2H), 2.58 (t, *J* = 7.7 Hz, 2H), 1.79 (quint, *J* = 6.6 Hz, 2H), 1.37 (br s, 1H); ^13^C­{^1^H} NMR (100 MHz, CDCl_3_) δ 157.8 (C), 133.9 (C), 129.3 (CH), 113.8 (CH), 62.3 (CH_2_), 55.2 (CH_3_), 34.4 (CH_2_), 31.1 (CH_2_); HRMS (ESI-TOF) *m*/*z*: [(M + H) −H_2_O]^+^ Calcd for C_10_H_13_O, 149.0961; Found, 149.0965.

### 1,1-Diphenylpropan-2-ol **(23)**

The general procedure for the synthesis of primary alcohols with BH_3_·SMe_2_ and TfOH was followed. ^1^H NMR and ^13^C­{^1^H} NMR spectroscopic, and HPLC chromatographic analysis of the unpurified reaction mixture revealed that primary and secondary alcohols were formed as a 99:1 mixture of regioisomers. Purification by flash column chromatography (20:80 EtOAc/hexanes) afforded the major regioisomer **23** as a white solid (0.170 g, 80%). The spectroscopic data are consistent with the data reported in the literature:^40^ mp 61–63 °C; ^1^H NMR (400 MHz, CDCl_3_) δ 7.43–7.18 (m, 10H), 4.62–4.54 (m, 1H), 3.83 (d, *J* = 7.7 Hz, 1H), 1.66 (d, *J* = 4.1 Hz, 1H), 1.20 (d, *J* = 6.7 Hz, 3H); ^13^C­{^1^H} NMR (100 MHz, CDCl_3_) δ 142.6 (C), 141.6 (C), 129.0 (CH), 128.7 (CH), 128.6 (CH), 128.3 (CH), 127.0 (CH), 126.6 (CH), 70.2 (CH), 60.7 (CH), 21.5 (CH_3_); HRMS (ESI-TOF) *m*/*z*: [(M + H) −H_2_O]^+^ Calcd for C_15_H_15_, 195.1174; Found, 195.1172.

### (1*S*,2*R*)-2-Phenylcyclohexan-1-ol **(25)**

The general procedure for the synthesis of primary alcohols with BH_3_·SMe_2_ and TfOH was followed. ^1^H NMR and ^13^C­{^1^H} NMR spectroscopic, and HPLC chromatographic analysis of the unpurified reaction mixture revealed that primary and secondary alcohols were formed as a 99:1 mixture of regioisomers. Purification by flash column chromatography (20:80 EtOAc/hexanes) afforded the major regioisomer **25** as a white solid (0.145 g, 82%). The spectroscopic data are consistent with the data reported in the literature:^41^ mp 66–68 °C; ^1^H NMR (400 MHz, CDCl_3_) δ 7.38–7.33 (m, 2H), 7.30–7.24 (m, 3H), 3.72–3.64 (m, 1H), 2.49–2.41 (m, 1H), 2.17–2.11 (m, 1H), 1.93–1.87 (m, 2H), 1.83–1.76 (m, 1H), 1.63–1.33 (m, 5H); ^13^C­{^1^H} NMR (100 MHz, CDCl_3_) δ 143.3 (C), 128.9 (CH), 128.0 (CH), 126.9 (CH), 74.5 (CH), 53.3 (CH), 34.5 (CH_2_), 33.4 (CH_2_), 26.2 (CH_2_), 25.2 (CH_2_); HRMS (ESI-TOF) *m*/*z*: [(M + H) −H_2_O]^+^ Calcd for C_12_H_15_, 159.1172; Found, 129.1168.

### 4-Phenylbutan-1-ol **(27)**

The general procedure for the synthesis of primary alcohols with BH_3_·SMe_2_ and TfOH. ^1^H NMR and ^13^C­{^1^H} NMR spectroscopic, and HPLC chromatographic analysis of the unpurified reaction mixture revealed that primary and secondary alcohols were formed as a 99:1 mixture of regioisomers. Purification by flash column chromatography (20:80 EtOAc/hexanes) afforded the major regioisomer **27** as a clear oil (0.135 g, 90%). The spectroscopic data are consistent with the data reported in the literature:^42^ ^1^H NMR (400 MHz, CDCl_3_) δ 7.33–7.28 (m, 2H), 7.23–7.19 (m, 3H), 3.67 (t, *J* = 6.7 Hz, 2H), 2.68 (t, *J* = 7.6 Hz, 2H), 1.77–1.71 (m, 2H), 1.66–1.61 (m, 2H); ^13^C­{^1^H} NMR (100 MHz, CDCl_3_) δ 142.3 (C), 128.4 (CH), 128.3 (CH), 125.7 (CH), 62.7 (CH_2_), 35.6 (CH_2_), 32.3 (CH_2_), 27.5 (CH_2_); HRMS (ESI-TOF) *m*/*z*: [(M + H) −H_2_O]^+^ Calcd for C_10_H_13_, 133.1012; Found, 133.1007.

### ((1*S*,2*S*,5*S*)-6,6-Dimethylbicyclo­[3.1.1]­heptan-2-yl)­methanol **(29)**

Reported procedures^30,31^ were modified to prepare alcohol **27**. To a cooled (0 °C) solution of alkene **26** (0.429 g, 3.15 mmol, 1 equiv) in CH_2_Cl_2_ (10.0 mL, 0.3 M) was added BH_3_·SMe_2_ (0.324 mL, 3.47 mmol, 1.1 equiv) dropwise. After 1 h, *tert*-butyl hydroperoxide (5.0–6.0 M in decane, 0.800 mL, 4.0 mmol, 1.3 equiv) and CsOH·H_2_O (0.600 g, 4.00 mmol, 1.3 equiv) were added. After 1 h, Na_2_S_2_O_3_ (3.00 g) and H_2_O (10.0 mL) was added to the reaction mixture. The mixture was extracted with *t*-BuOMe (4 × 20 mL). The combined organic phases were washed with H_2_O (4 × 5 mL), 2 M aqueous NaOH (4 × 5 mL), and brine (4 × 10 mL). The combined organic phases were then dried over anhydrous MgSO_4_, filtered, and concentrated in vacuo. Purification by flash column chromatography (20:80 EtOAc/hexanes) afforded alcohol **27** as a clear oil (0.292 g, 60%). The spectroscopic data are consistent with the data reported in the literature:^43^ ^1^H NMR (400 MHz, CDCl_3_) δ 3.61–3.52 (m, 2H), 2.40–2.34 (m, 1H), 2.32–2.19 (m, 1H), 2.01 (ddd, *J* = 6.8, 5.0, 2.2, 1H), 1.98–1.83 (m, 4H), 1.49–1.39 (m, 2H), 1.18 (s, 3H), 0.97 (s, 3H), 0.93 (d, *J* = 9.6 Hz, 1H); ^13^C­{^1^H} NMR (100 MHz, CDCl_3_) δ 67.9 (CH_2_), 44.6 (CH), 43.0 (CH), 41.6 (C), 38.8 (CH), 33.3 (CH_2_), 28.1 (CH_2_), 26.1 (CH_2_), 23.5 (CH_3_), 18.9 (CH_3_); HRMS (ESI-TOF) *m*/*z*: [M + H]^+^ Calcd for C_10_H_18_O, 155.1430; Found, 155.1428.

## Supplementary Material



## Data Availability

The data underlying this study are available in the published article and the Supporting Information.

## Notes

^1^H,^13^C­{^1^H}, and ^11^B spectra for compounds, analysis of unpurified reaction mixtures with and without the addition of TfOH (PDF)
