# Supplementary material for: Regioselective Hydroboration of Terminal Alkenes with the Addition of Triflic Acid to Borane
Source: J Org Chem. 2026 Apr 30;91(19):6792–8. doi: 10.1021/acs.joc.5c03231 (PMC13185095; doi:10.1021/acs.joc.5c03231)

Regioselective Hydroboration of Terminal Alkenes with the Addition of Triflic Acid to Borane

Ilias Fourati[1] and K. A. Woerpel[1]*

[1]Department of Chemistry, New York University
100 Washington Square East, New York, New York 10003
Email: kwoerpel@nyu.edu

Supporting Information

Table of Contents

I. ^{1}H, $^{13}C\{^{1}H\}$, and ^{11}B NMR Spectra of Purified Compounds S2
II. ^{1}H and $^{13}C\{^{1}H\}$ NMR Spectra of Crude Mixtures S31

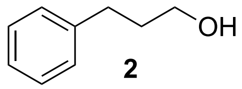

2

(^{1}H NMR, 400 MHz, $CDCl_3$)

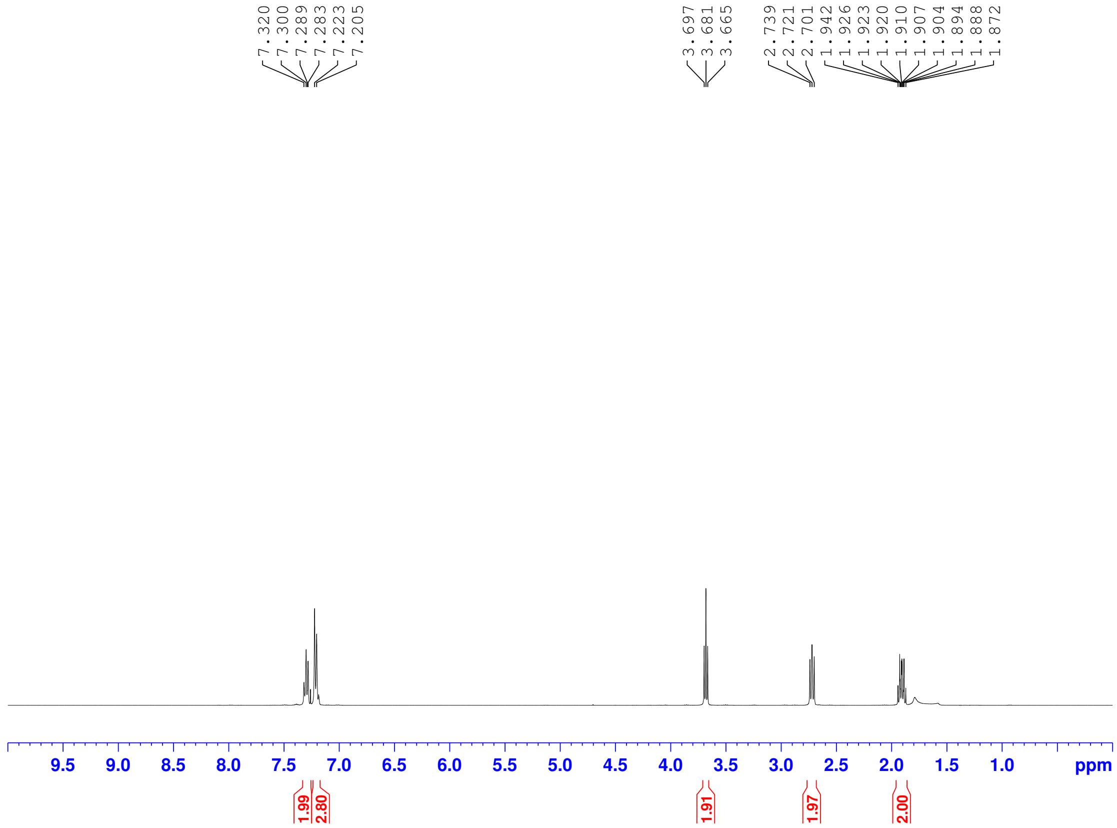

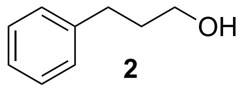

2

($^{13}C\{^1H\}$ NMR, 100 MHz, $CDCl_3$)

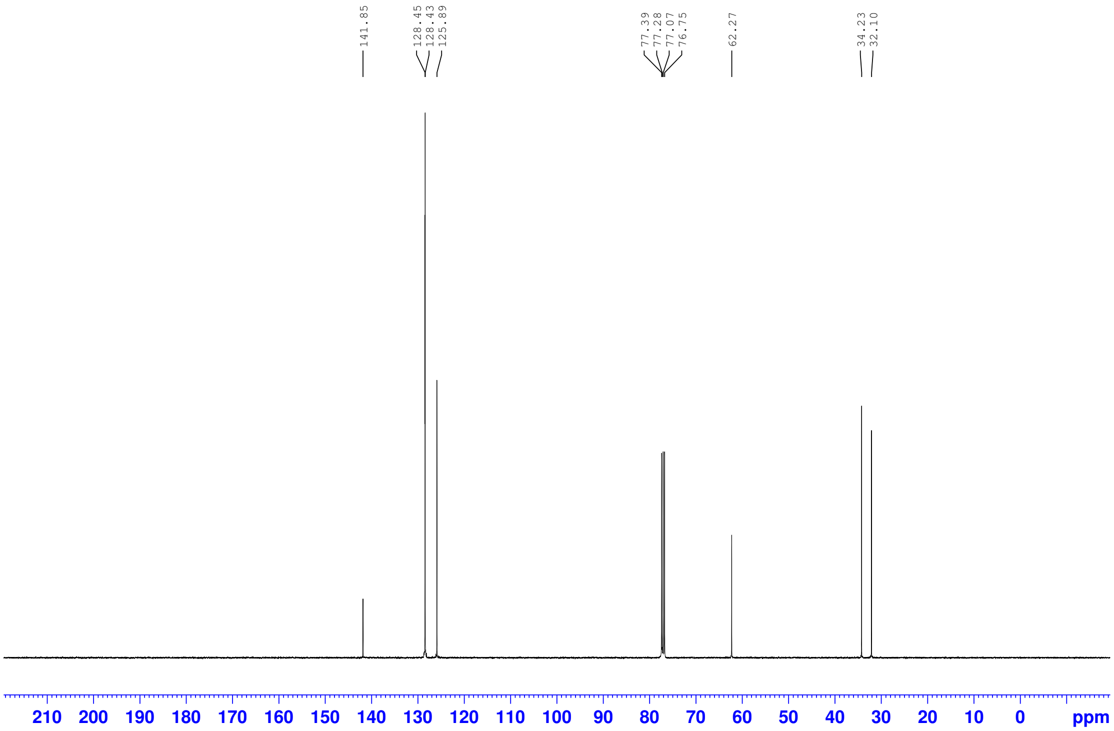

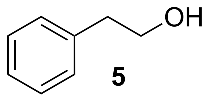

5

(^{1}H NMR, 400 MHz, $CDCl_3$)

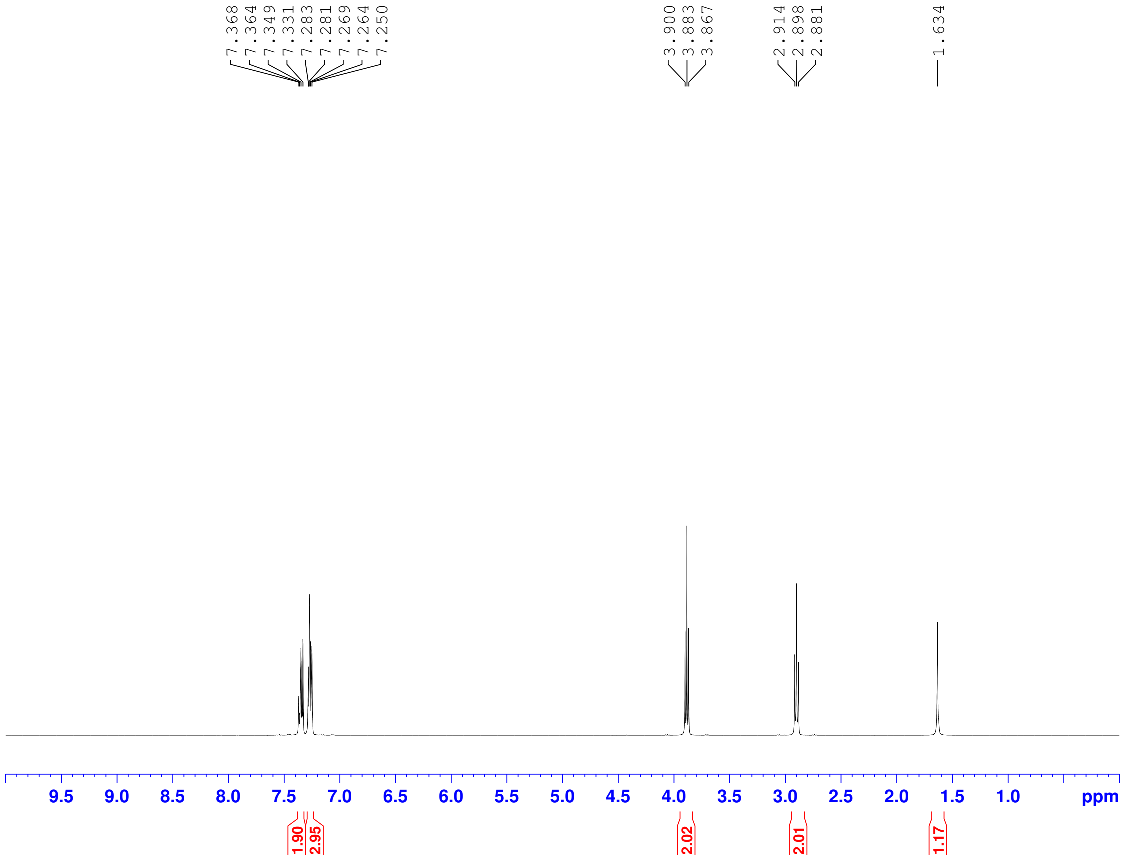

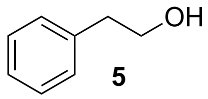

5

($^{13}C\{^{1}H\}$ NMR, 100 MHz, $CDCl_3$)

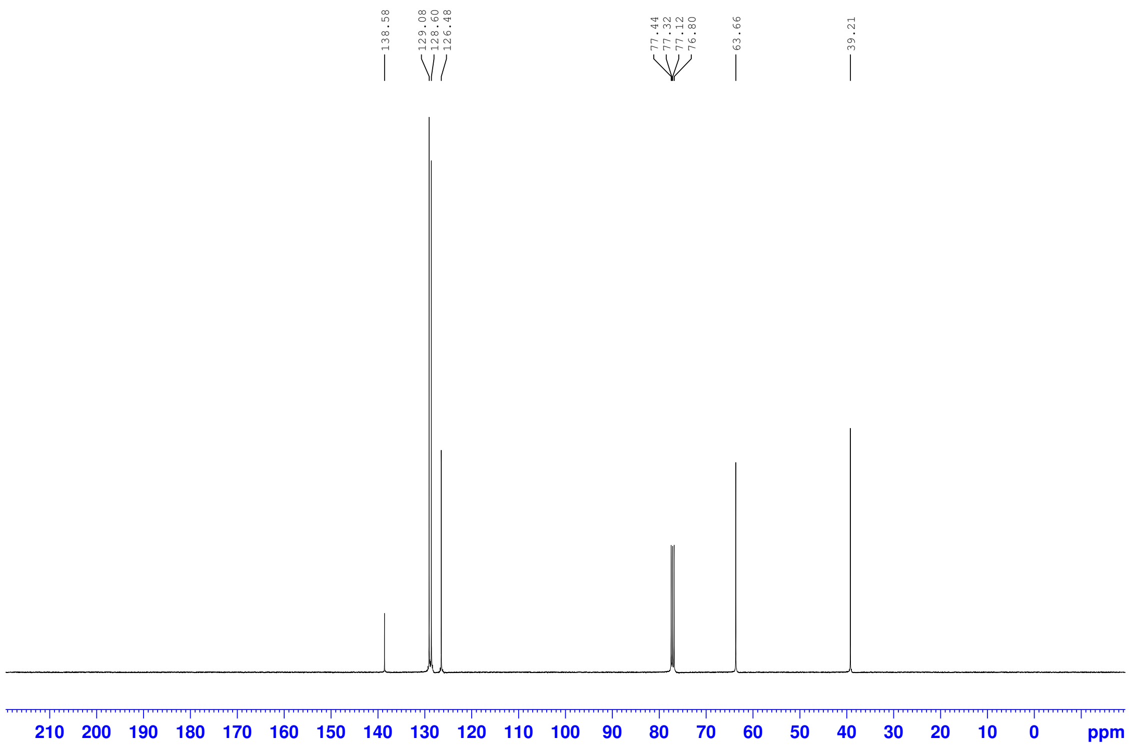

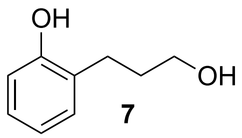

7

(^{1}H NMR, 400 MHz, $CDCl_3$)

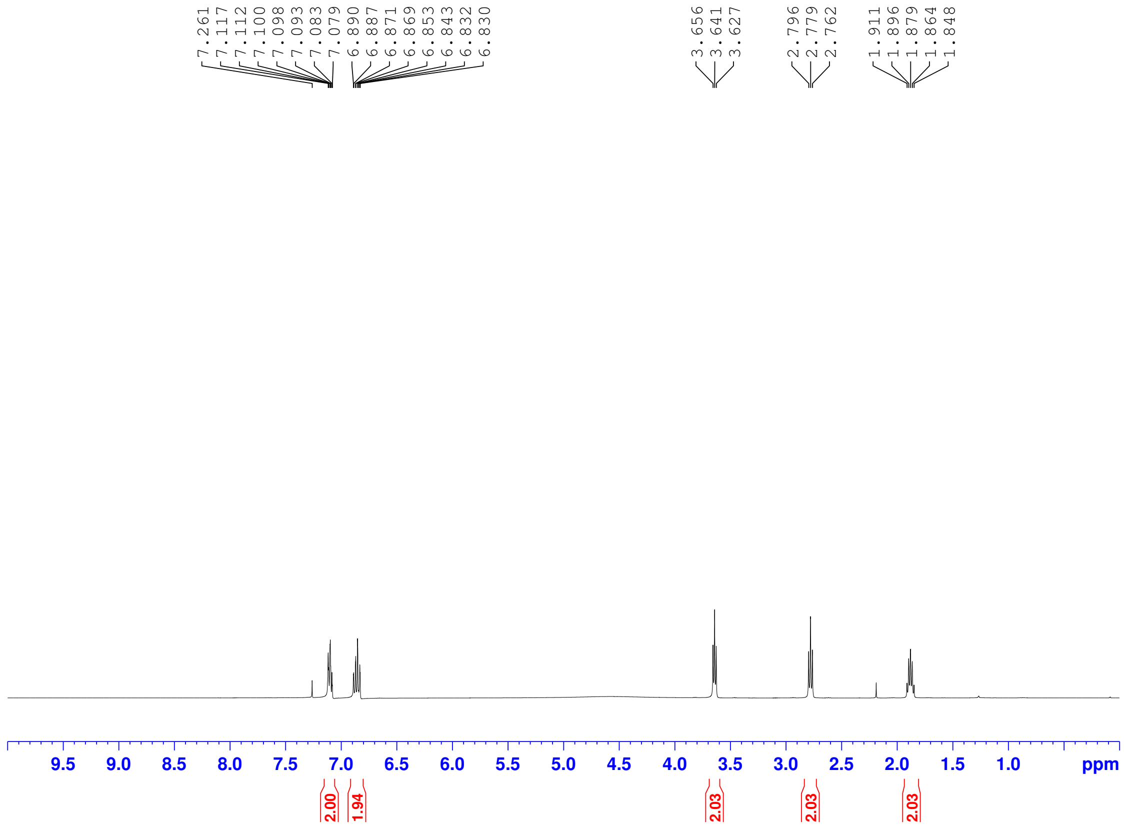

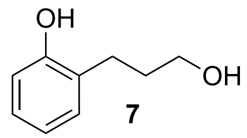

7

(^{13}C{^{1}H} NMR, 100 MHz, $CDCl_3$)

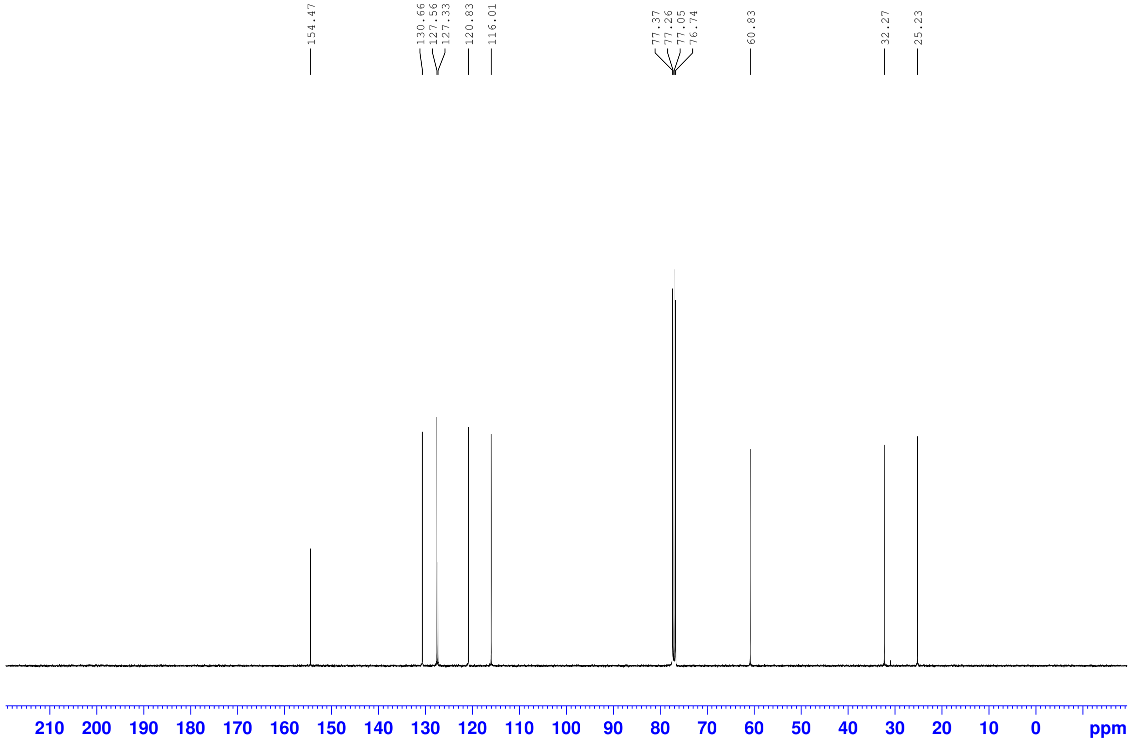

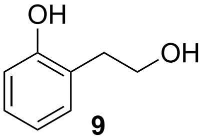

9

(¹H NMR, 400 MHz, $CDCl_3$)

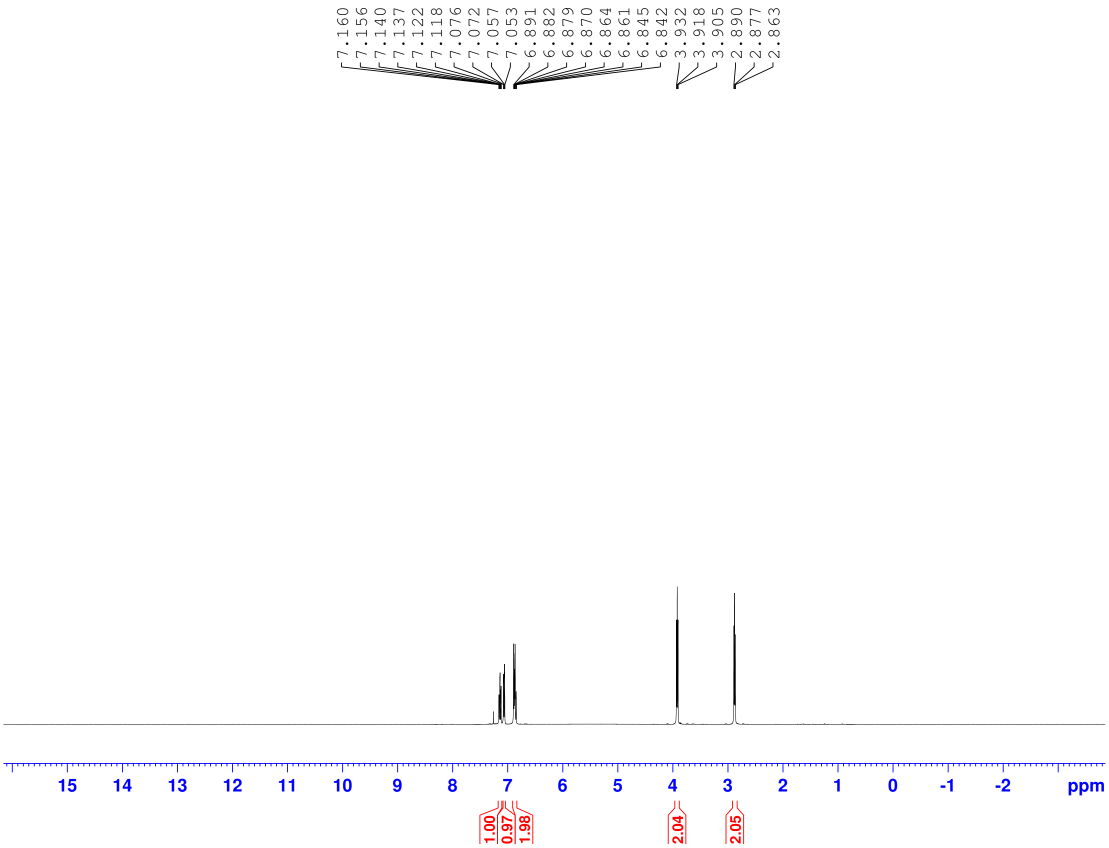

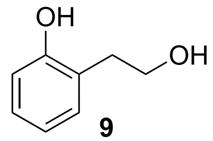

9

($^{13}C\{^{1}H\}$ NMR, 100 MHz, $CDCl_3$)

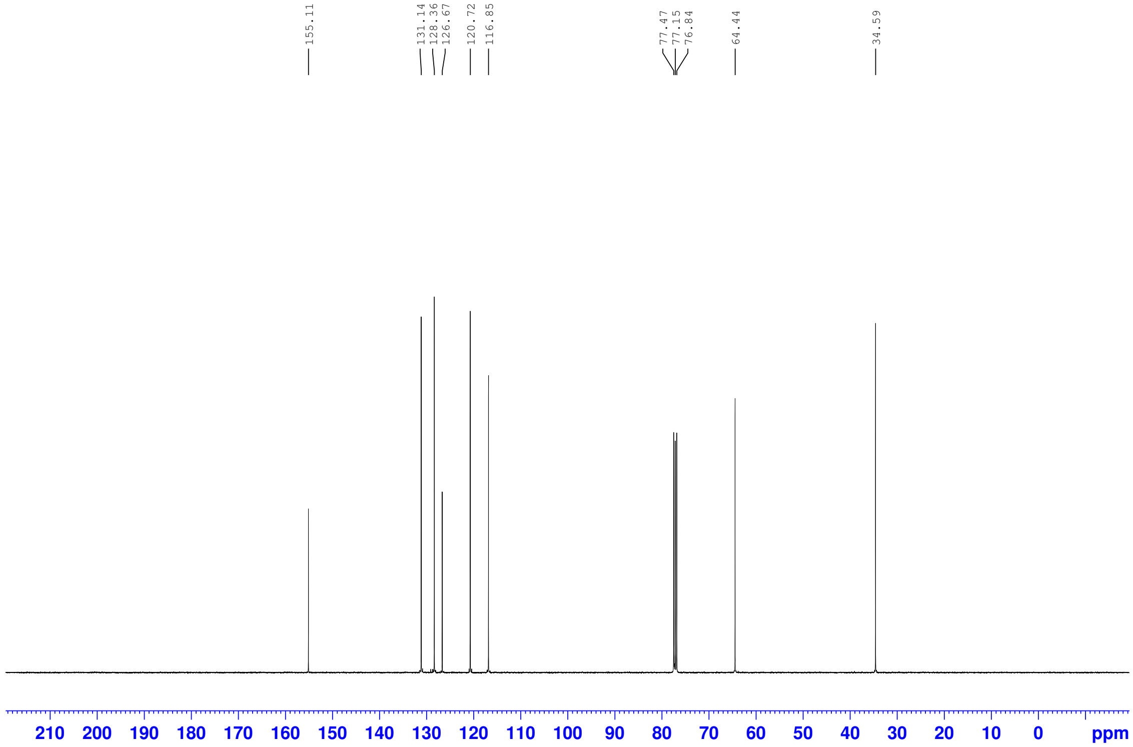

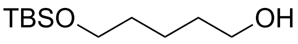

11

(^{1}H NMR, 400 MHz, $CDCl_3$)

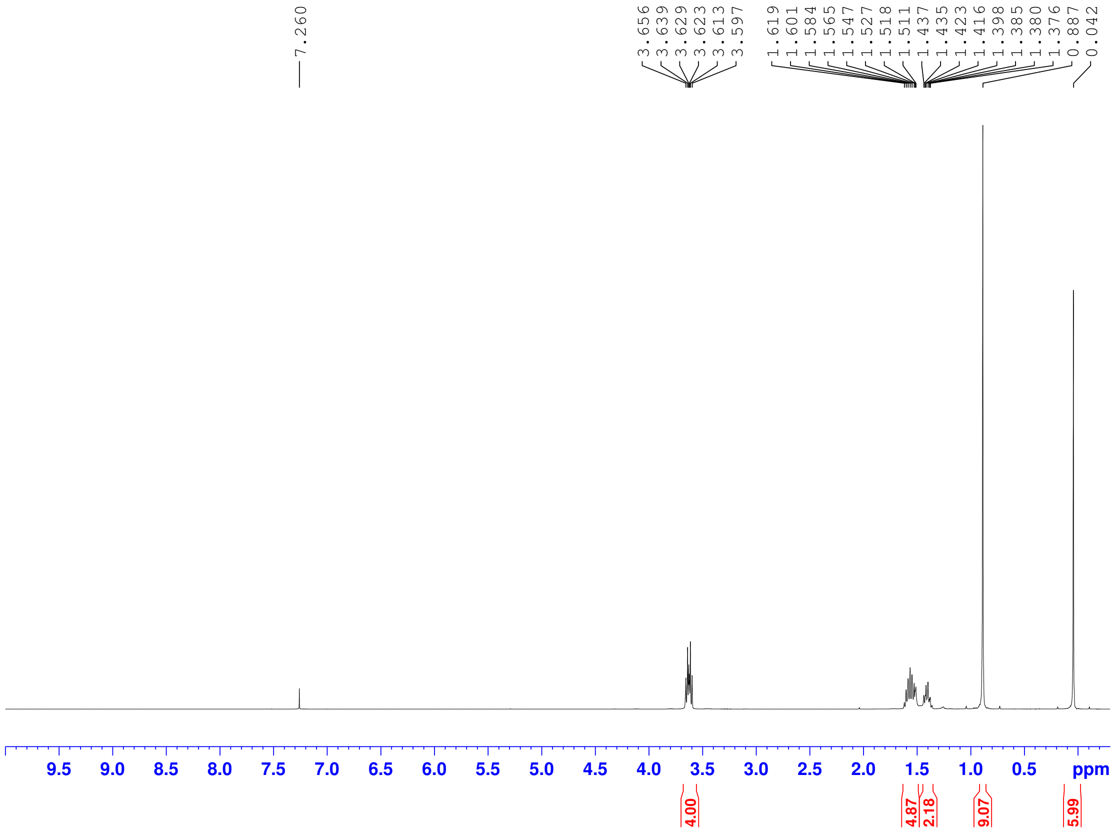

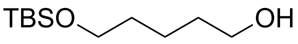

11

($^{13}C\{^{1}H\}$ NMR, 100 MHz, $CDCl_3$)

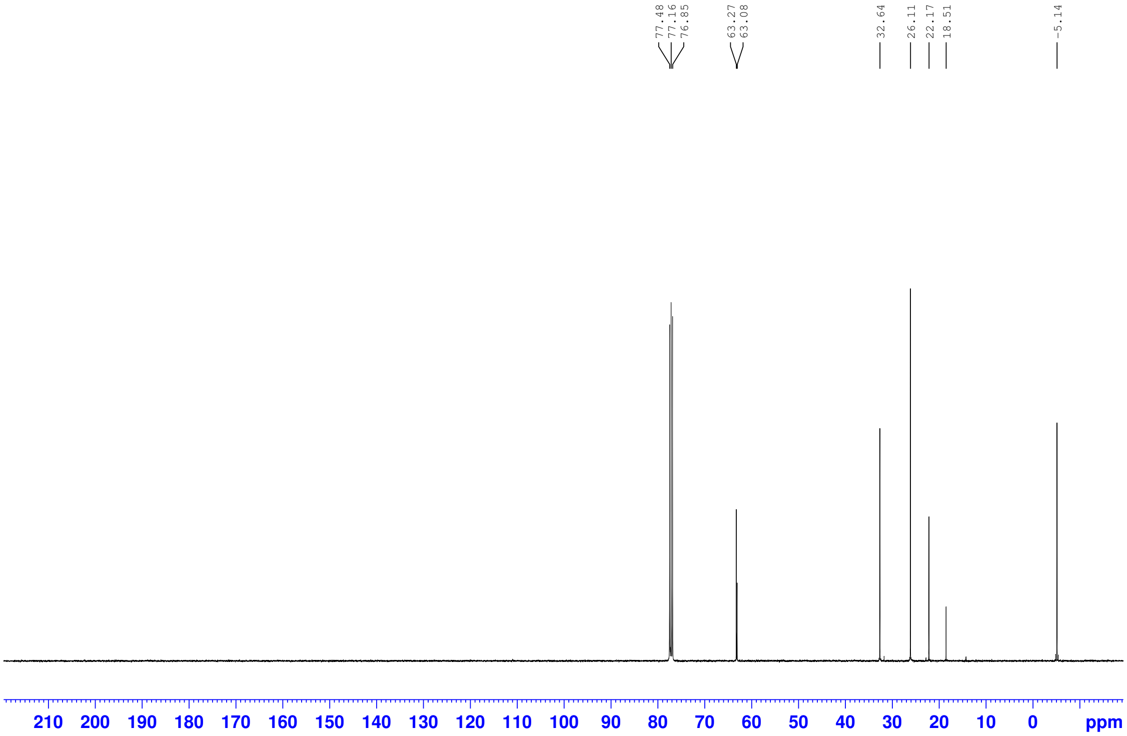

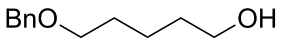

13

(^{1}H NMR, 400 MHz, $CDCl_3$)

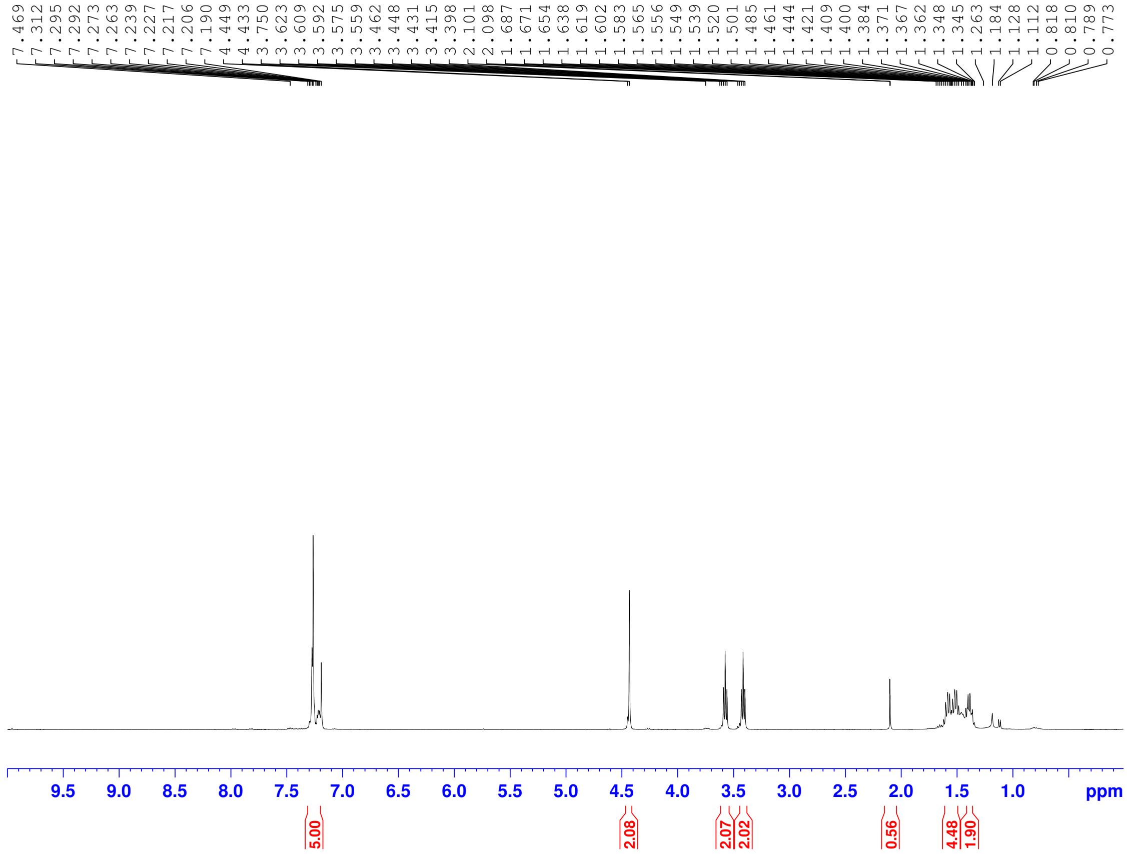

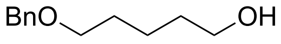

13

($^{13}C\{^{1}H\}$ NMR, 100 MHz, $CDCl_3$)

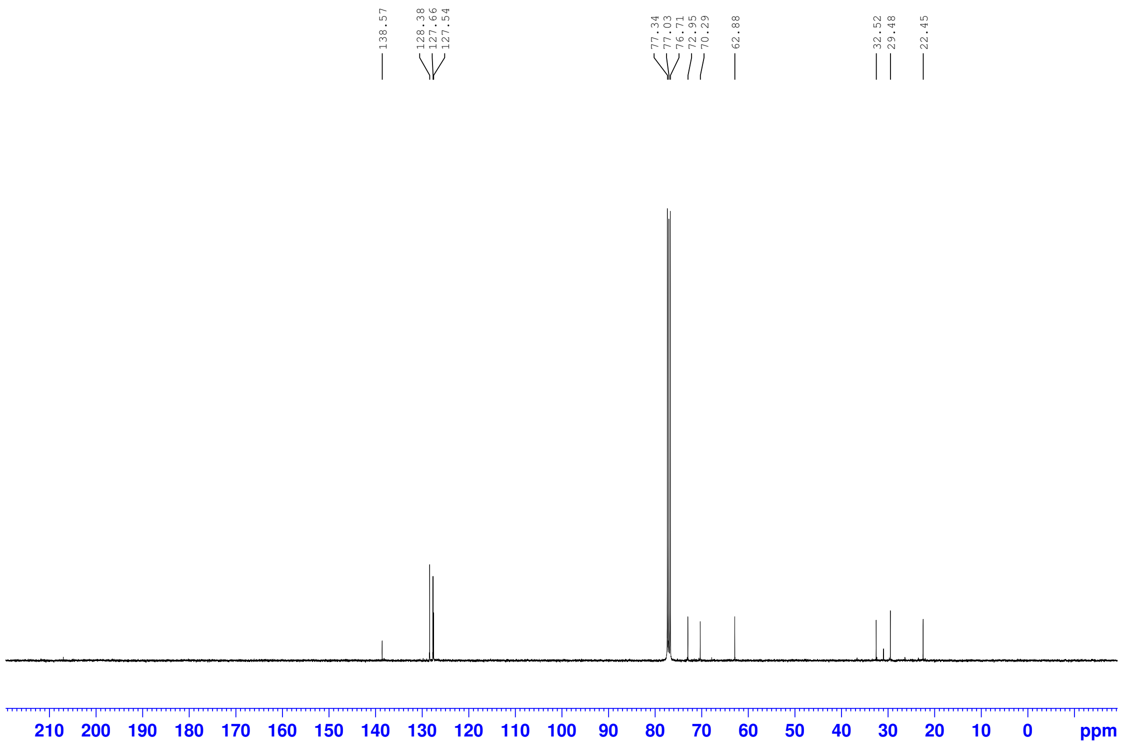

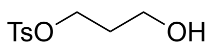

15 (^{1}H NMR, 400 MHz, $CDCl_3$)

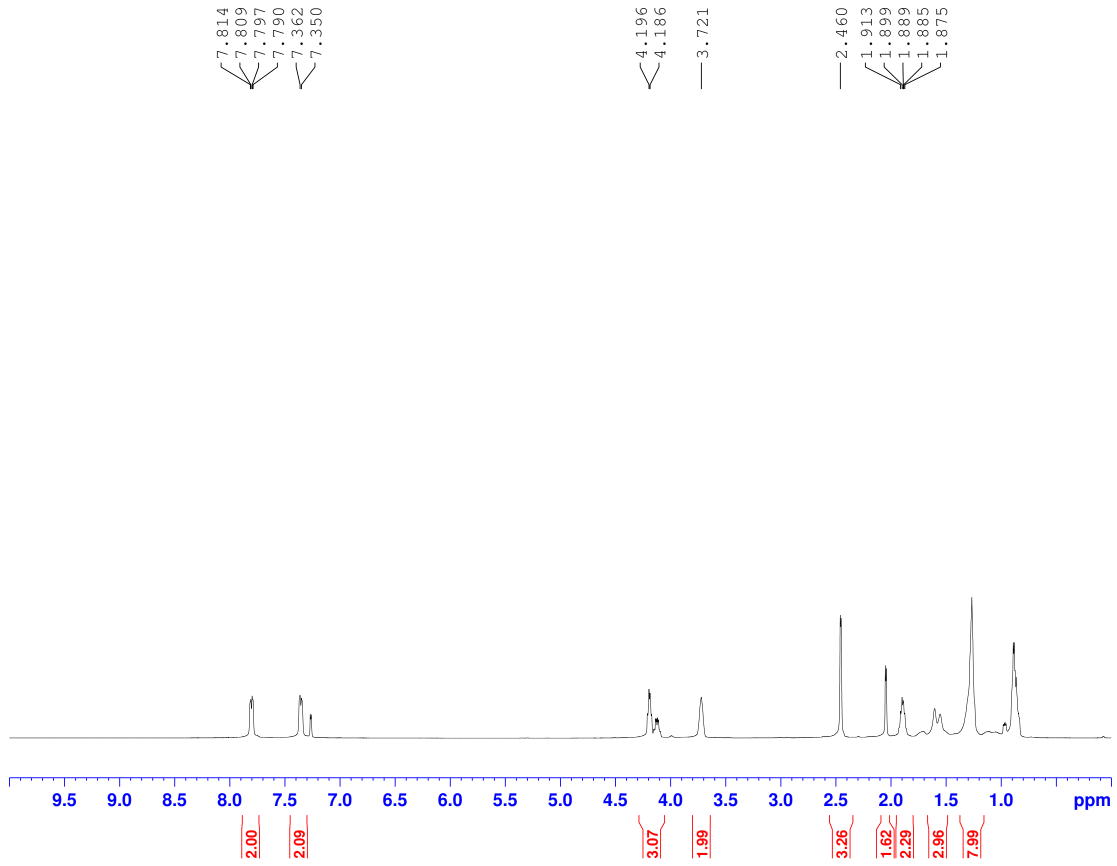

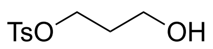

15 ($^{13}C\{^1H\}$ NMR, 100 MHz, $CDCl_3$)

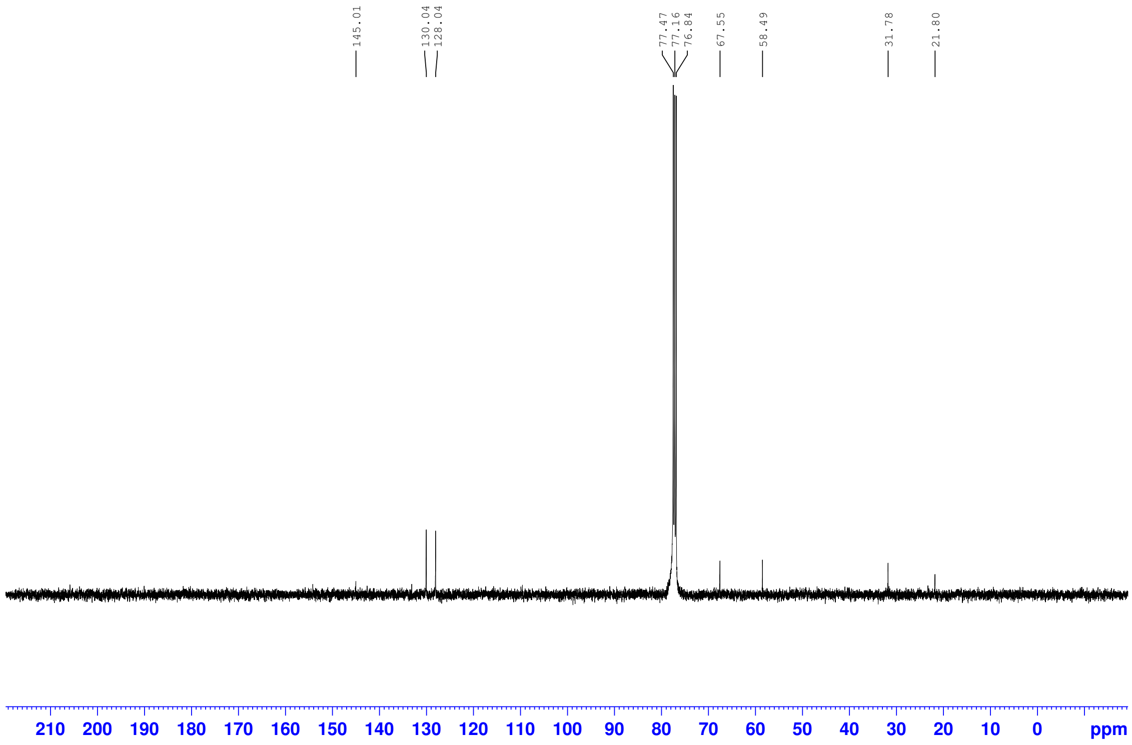

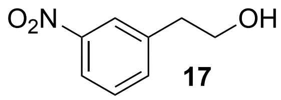

17

(^{1}H NMR, 400 MHz, $CDCl_3$)

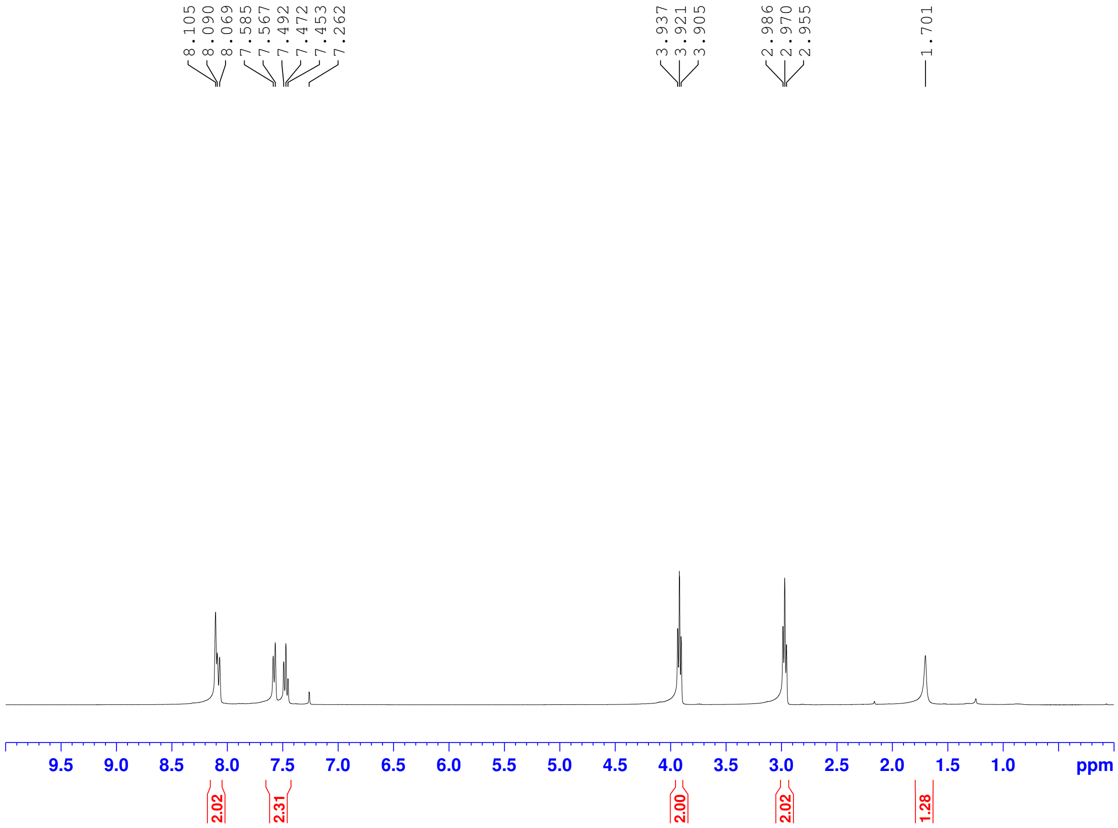

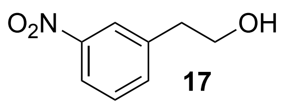

17

($^{13}C\{^{1}H\}$ NMR, 100 MHz, $CDCl_3$)

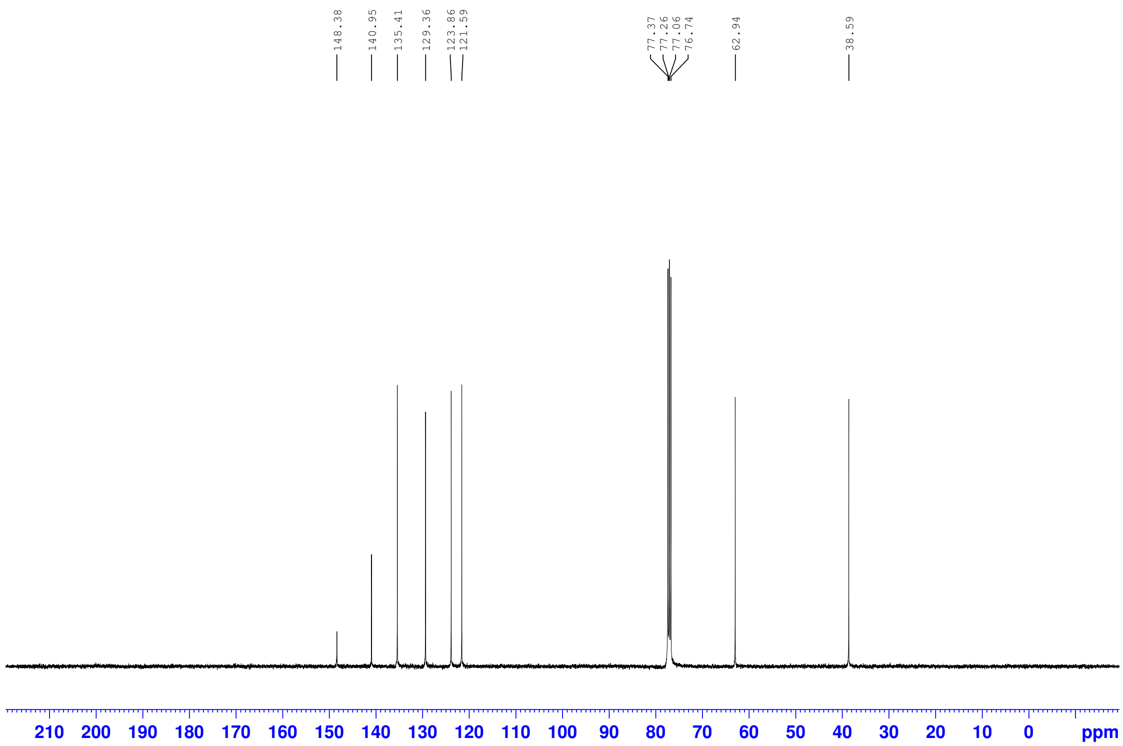

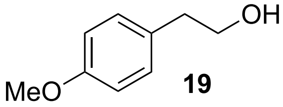

(^{1}H NMR, 400 MHz, $CDCl_3$)

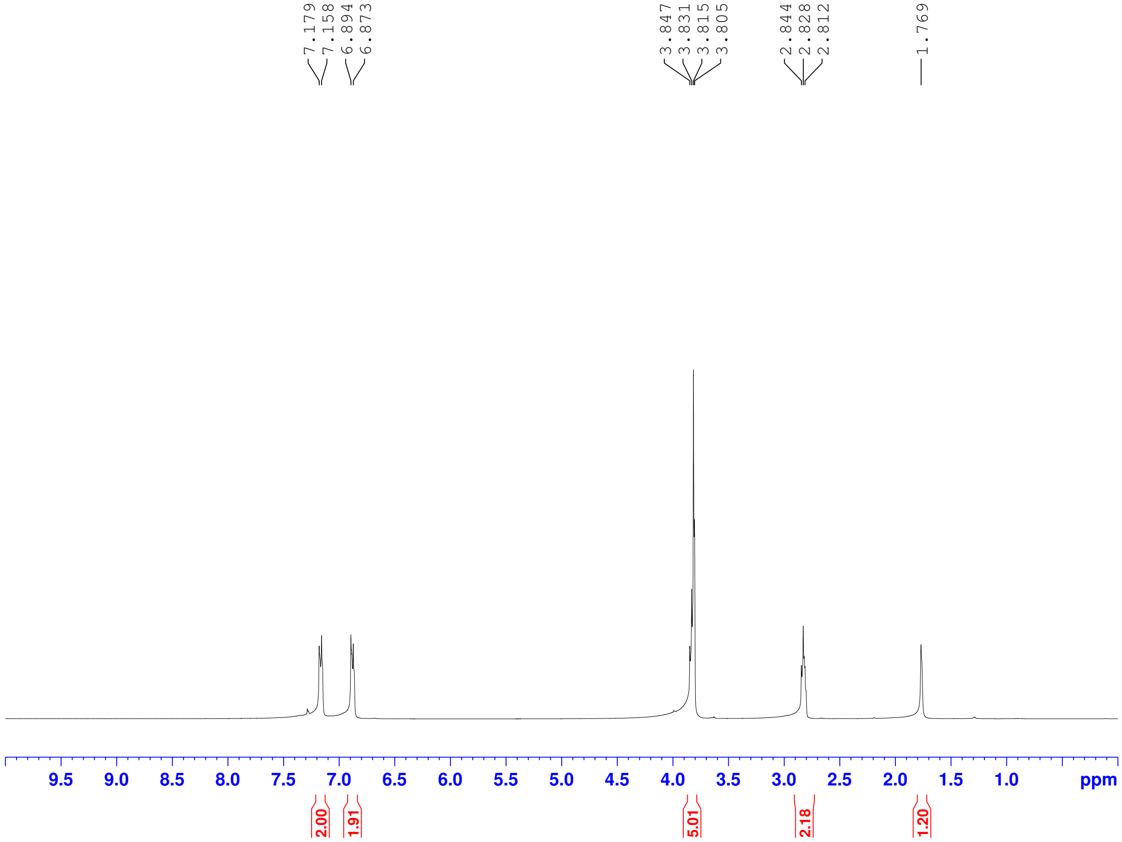

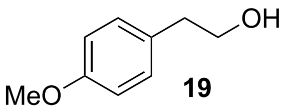

19

($^{13}C\{^{1}H\}$ NMR, 100 MHz, $CDCl_3$)

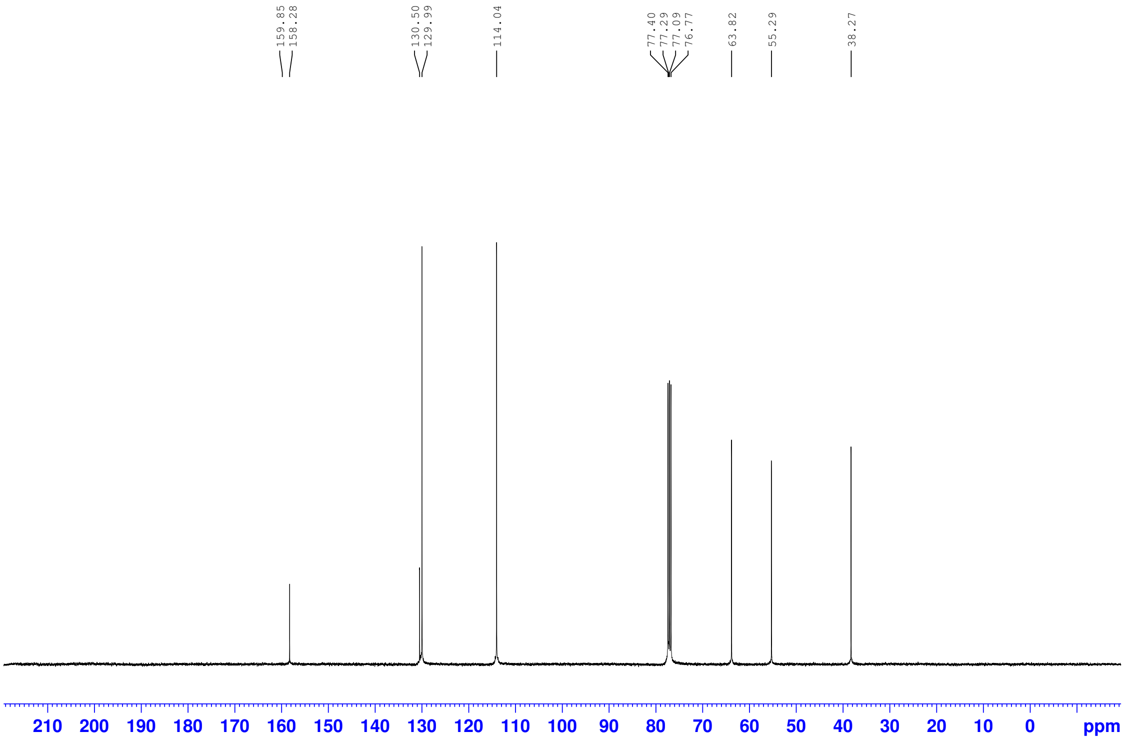

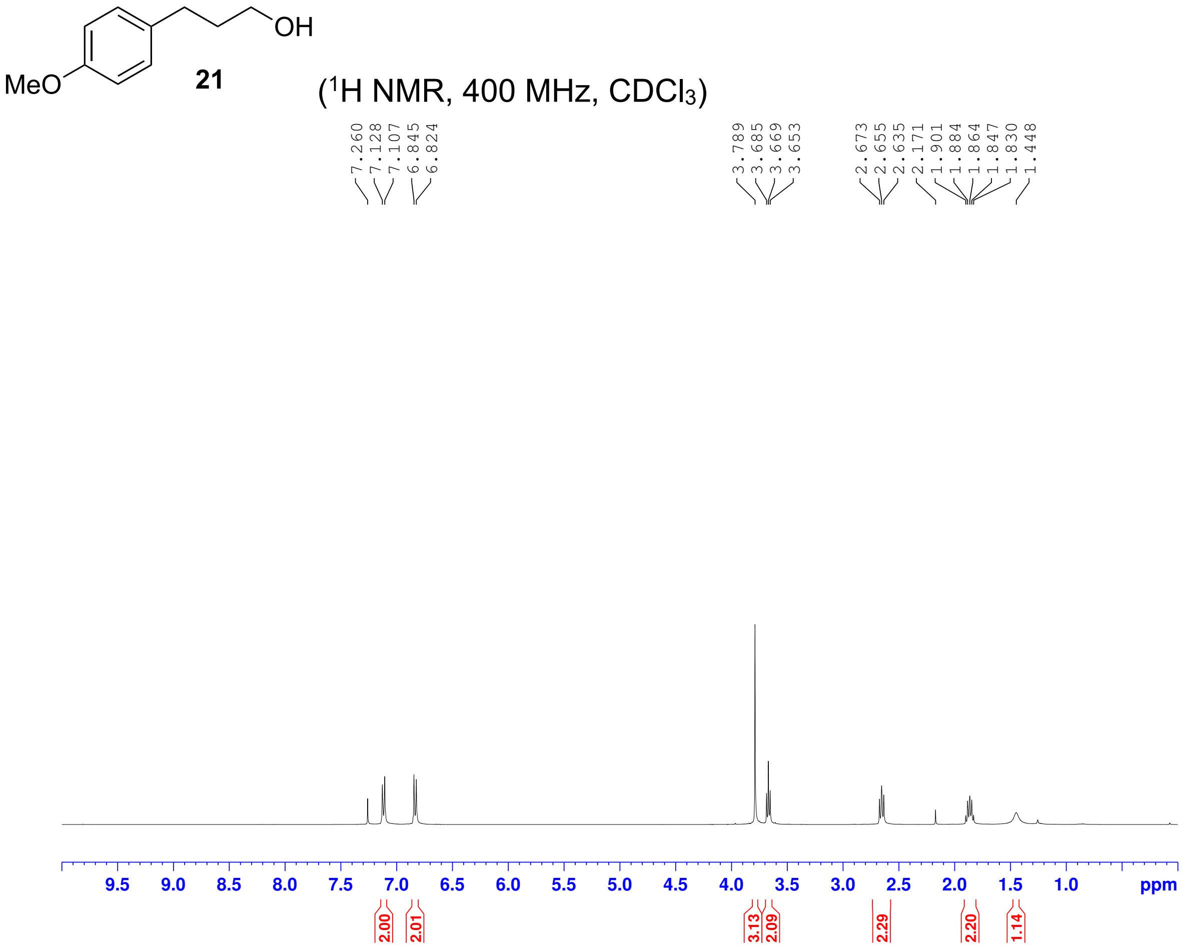
MeO
OH
21
(1H NMR, 400 MHz, CDCl3)
7.260
7.128
7.107
6.845
6.824
3.789
3.685
3.669
3.653
2.673
2.655
2.635
2.171
1.901
1.884
1.864
1.847
1.830
1.448
9.5
9.0
8.5
8.0
7.5
7.0
6.5
6.0
5.5
5.0
4.5
4.0
3.5
3.0
2.5
2.0
1.5
1.0
ppm
2.00
2.01
3.13
2.09
2.29
2.20
1.14

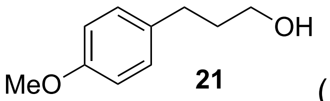

21

($^{13}C\{^{1}H\}$ NMR, 100 MHz, $CDCl_3$)

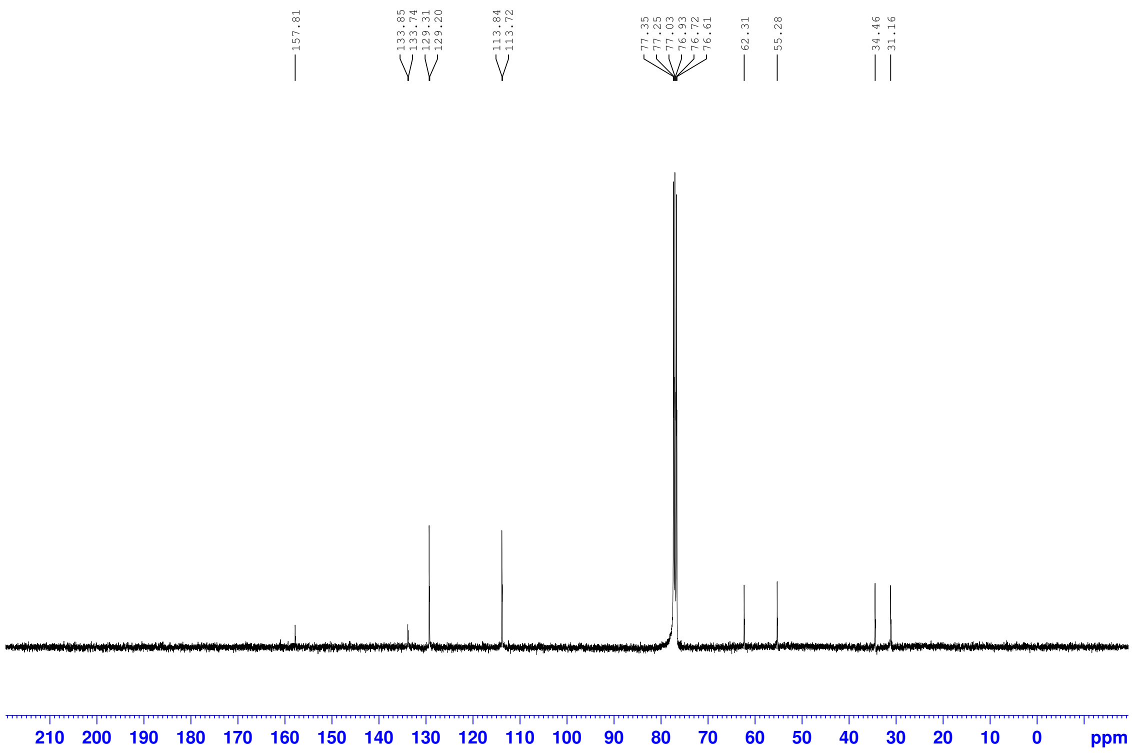

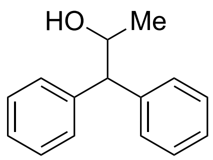

23

(^{1}H NMR, 400 MHz, $CDCl_3$)

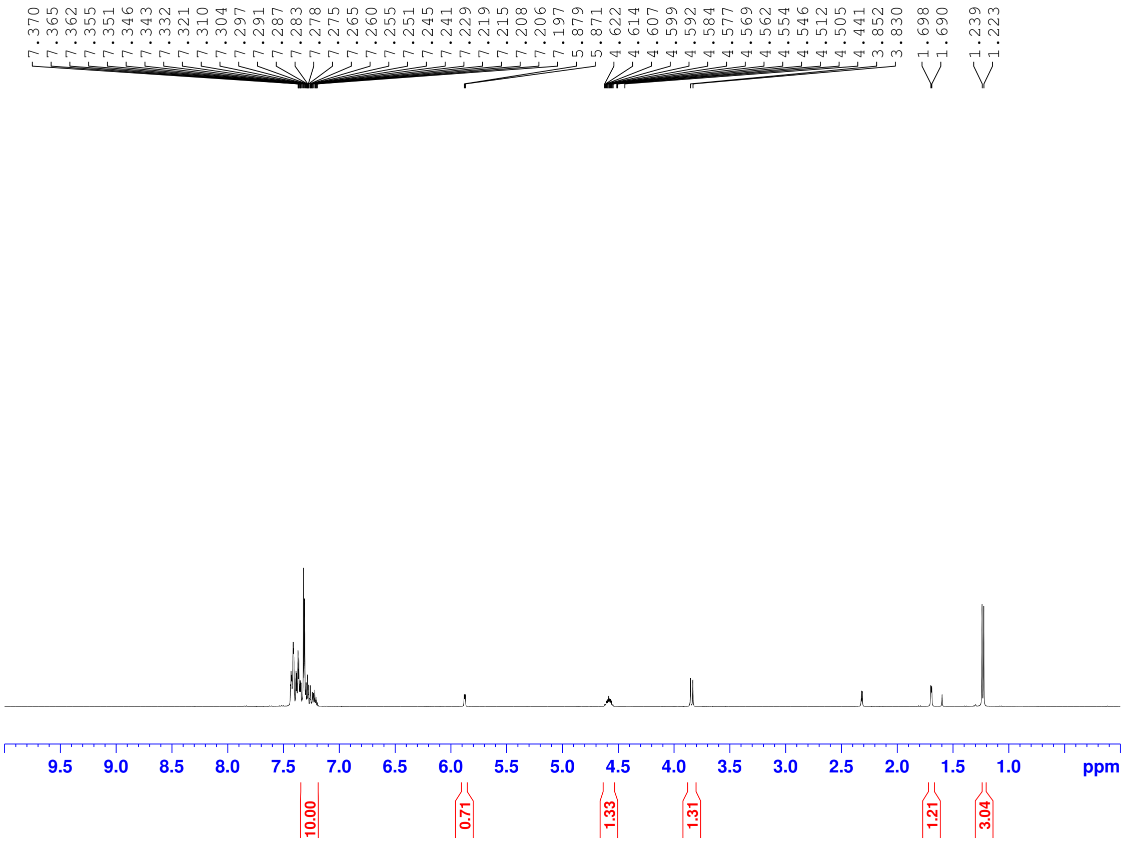

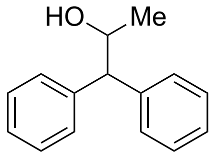

23

($^{13}C\{^{1}H\}$ NMR, 100 MHz, $CDCl_3$)

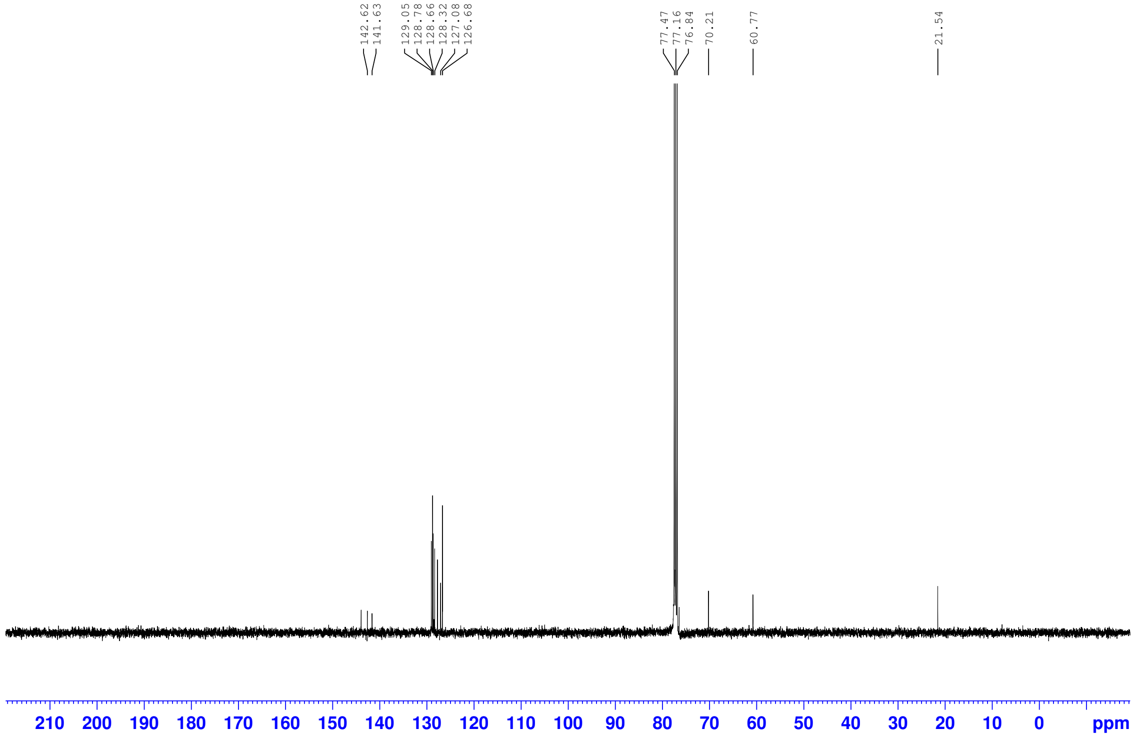

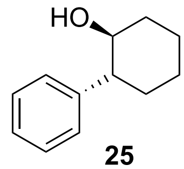

25 (^{1}H NMR, 400 MHz, $CDCl_3$)

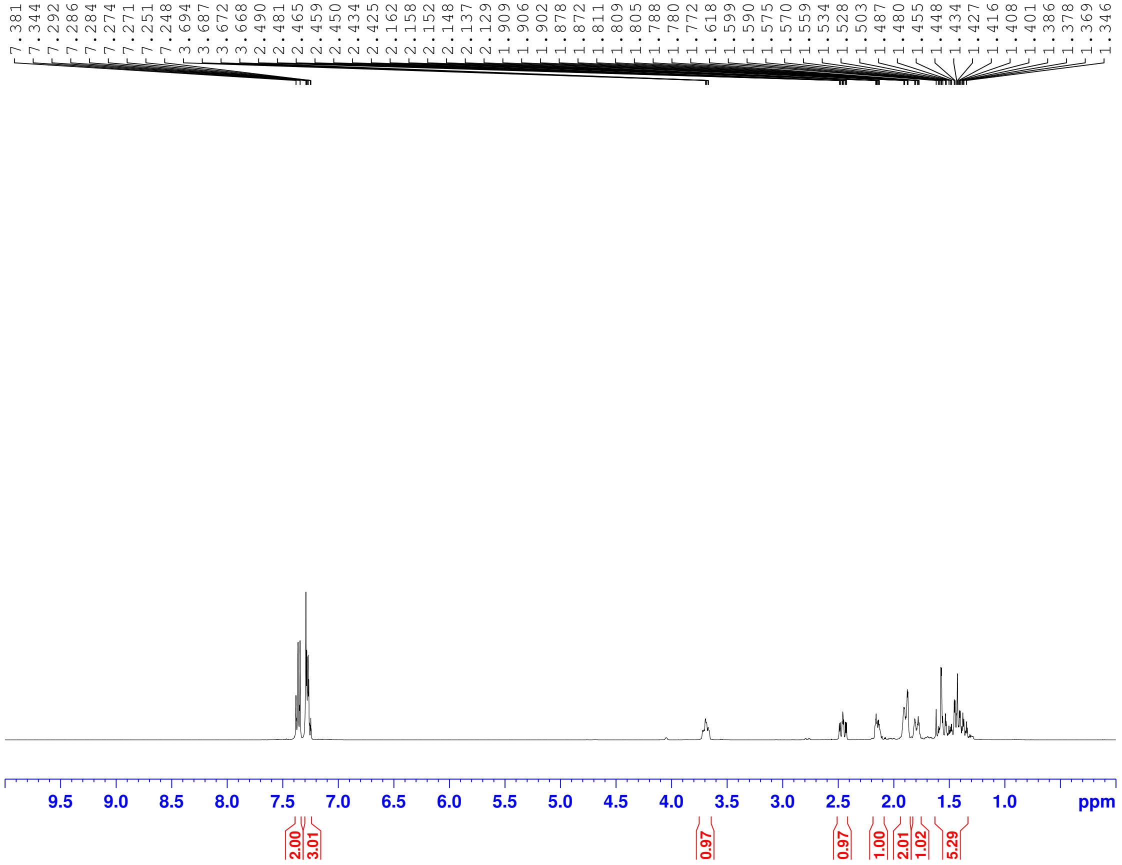

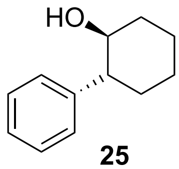

25 ($^{13}C\{^1H\}$ NMR, 100 MHz, $CDCl_3$)

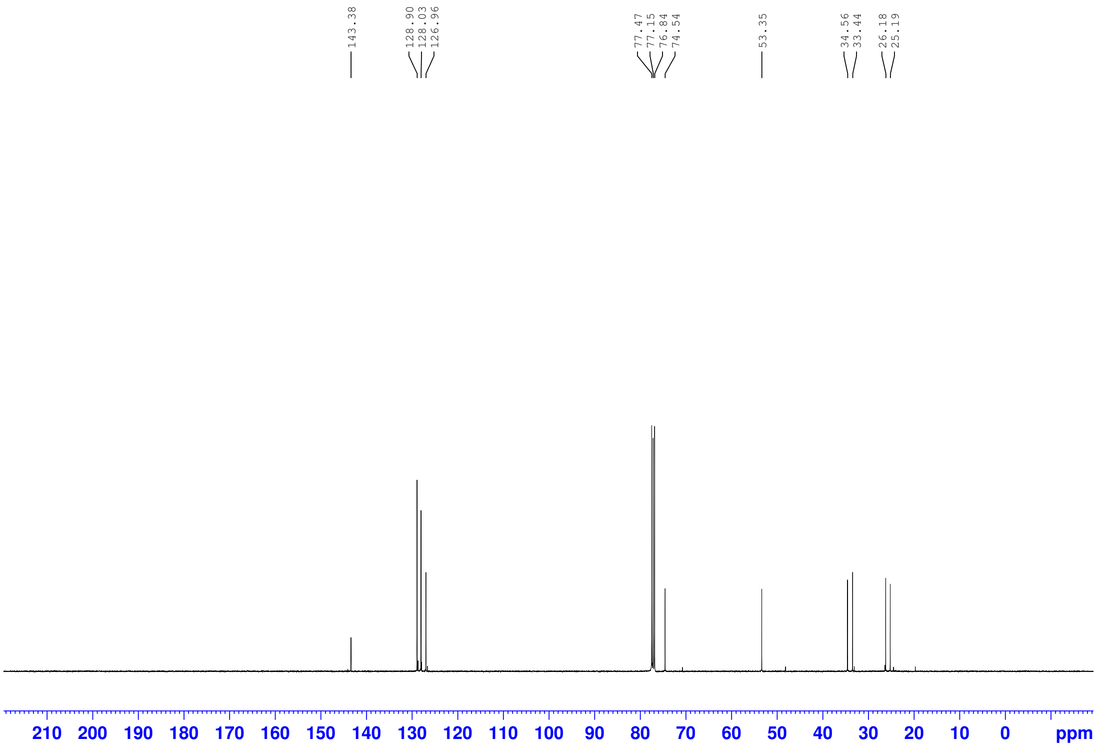

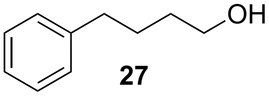

27

(^{1}H NMR, 400 MHz, $CDCl_3$)

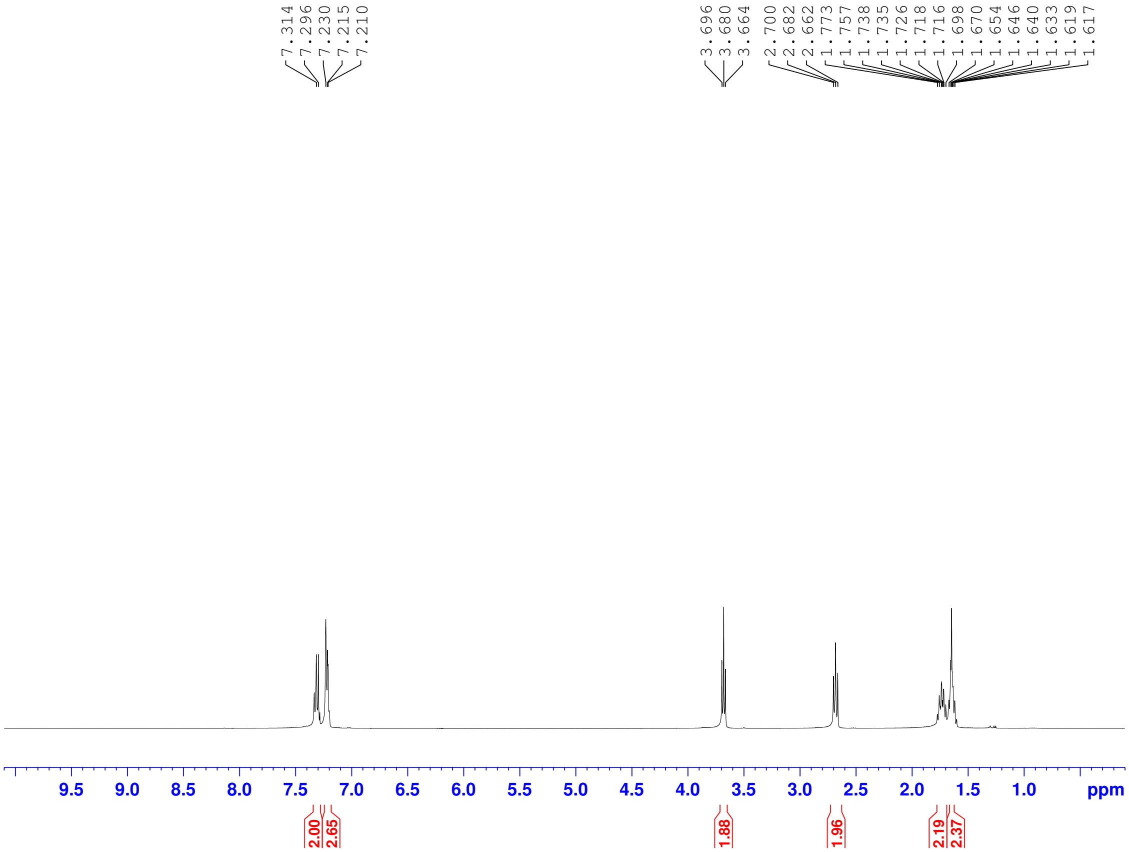

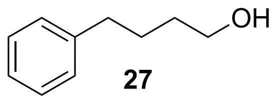

27

($^{13}C\{^{1}H\}$ NMR, 100 MHz, $CDCl_3$)

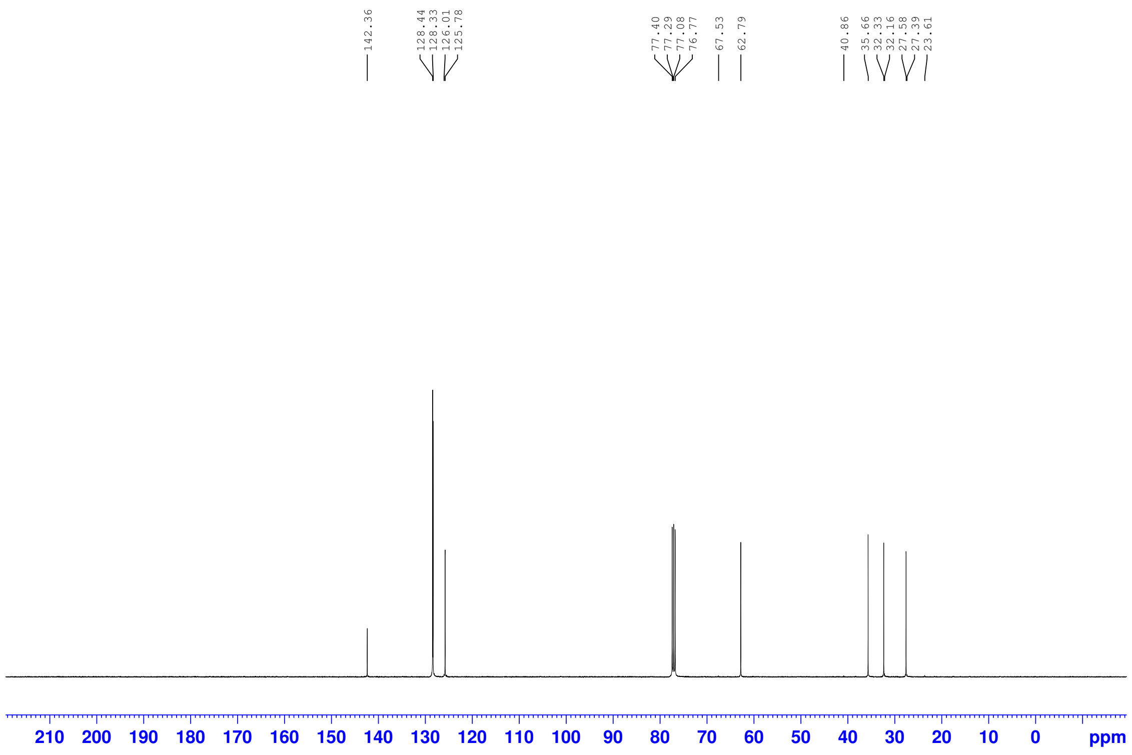

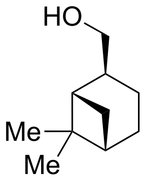

29 (^{1}H NMR, 400 MHz, $CDCl_3$)

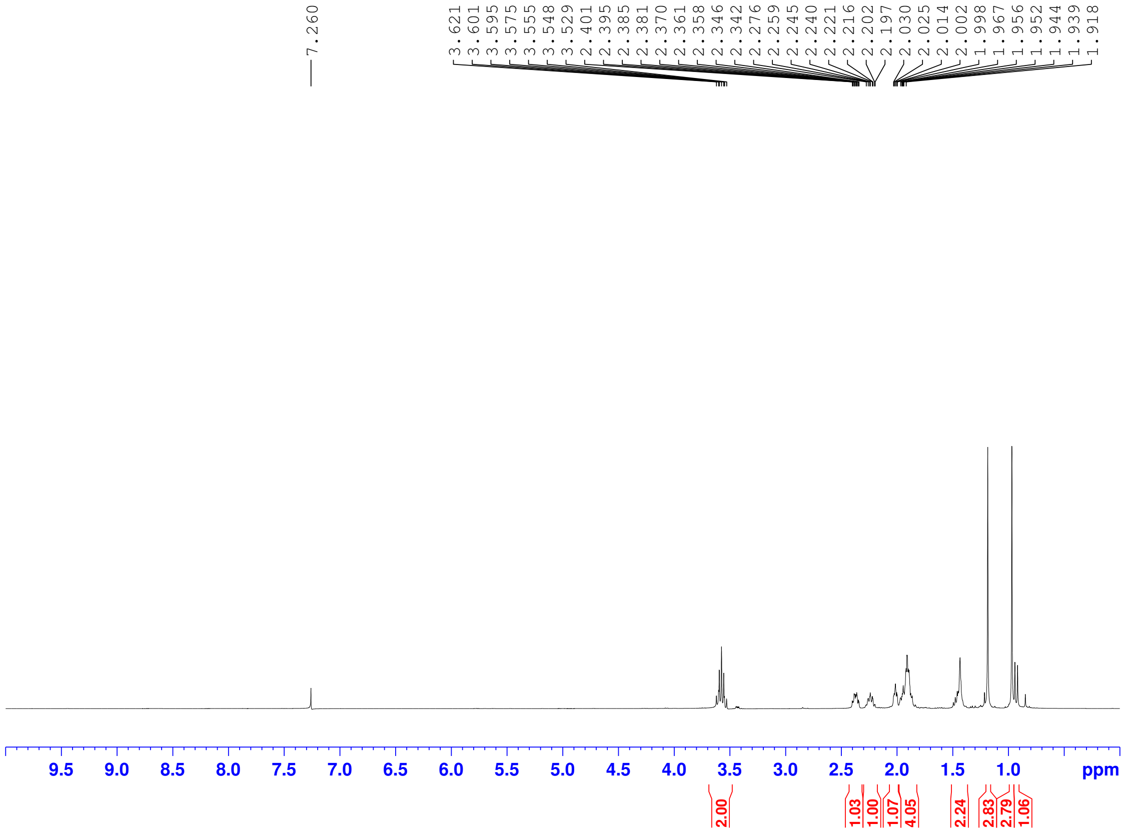

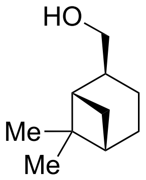

29 ($^{13}C\{^{1}H\}$ NMR, 100 MHz, $CDCl_3$)

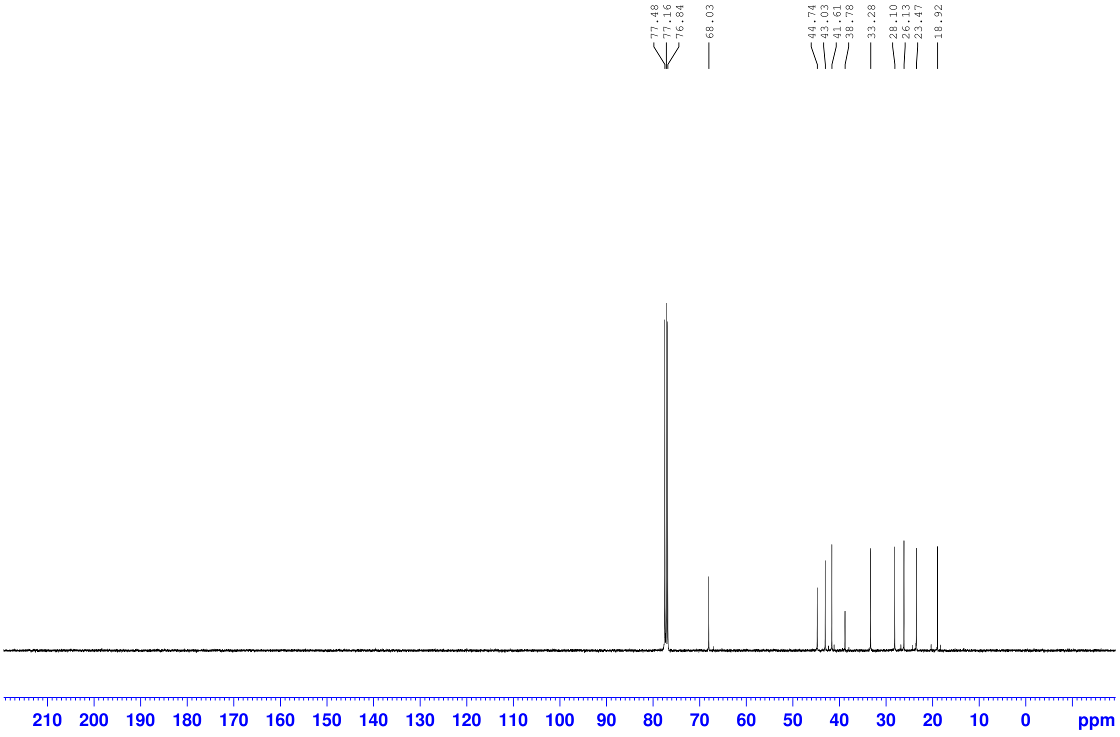

$H_2BOTf{\cdot}SMe_2$ (^{11}B NMR, 160 MHz, CH_2Cl_2)

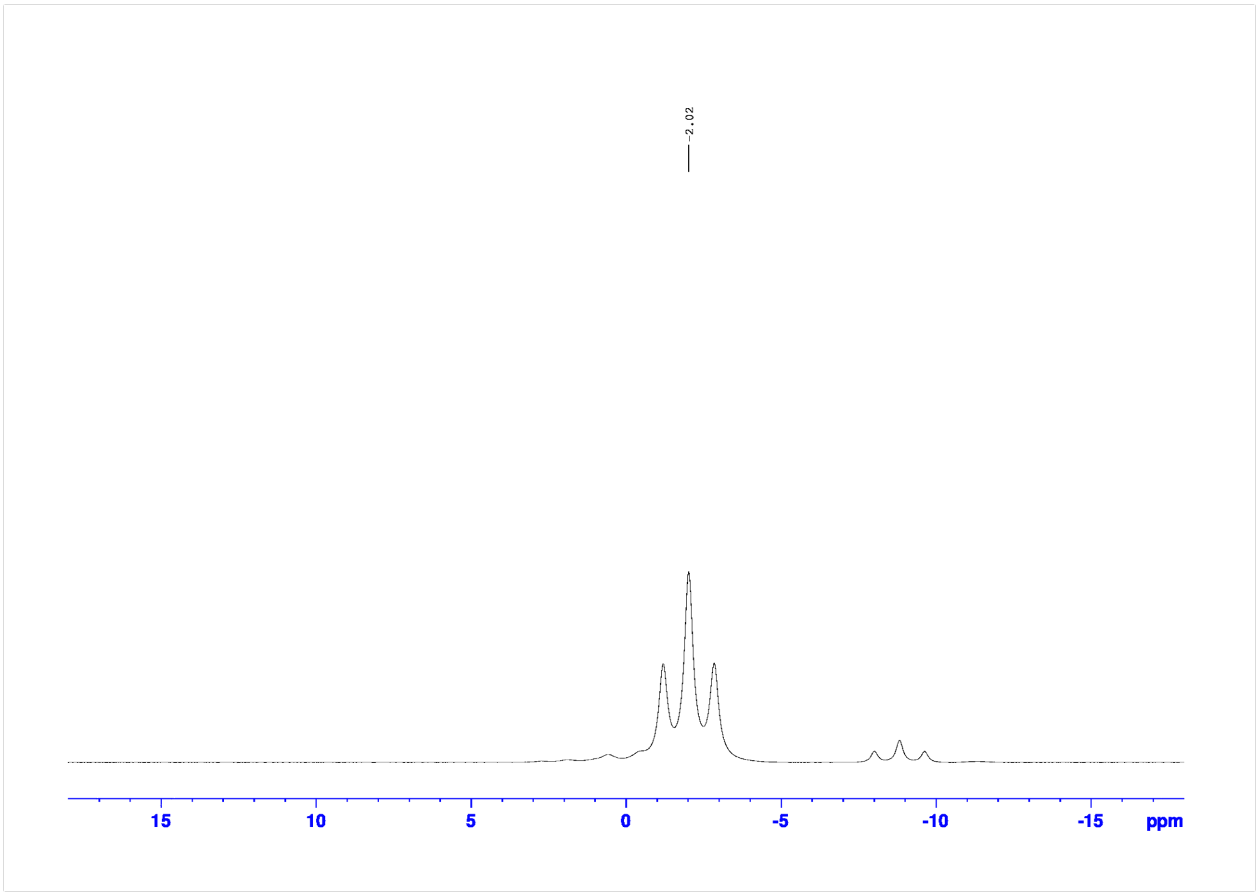

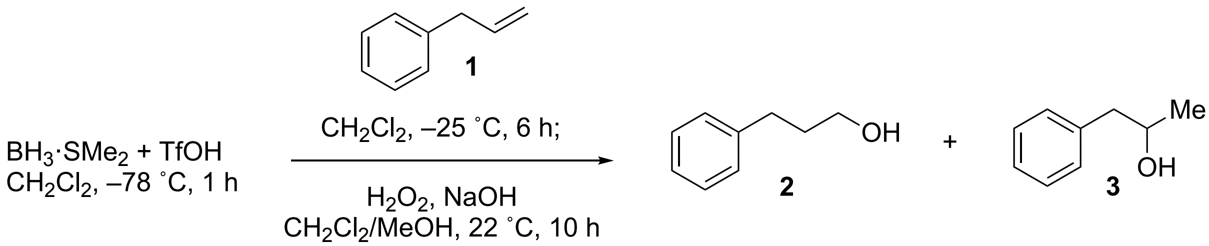

(Single-Pulse ^{1}H NMR, 400 MHz, $CDCl_3$)

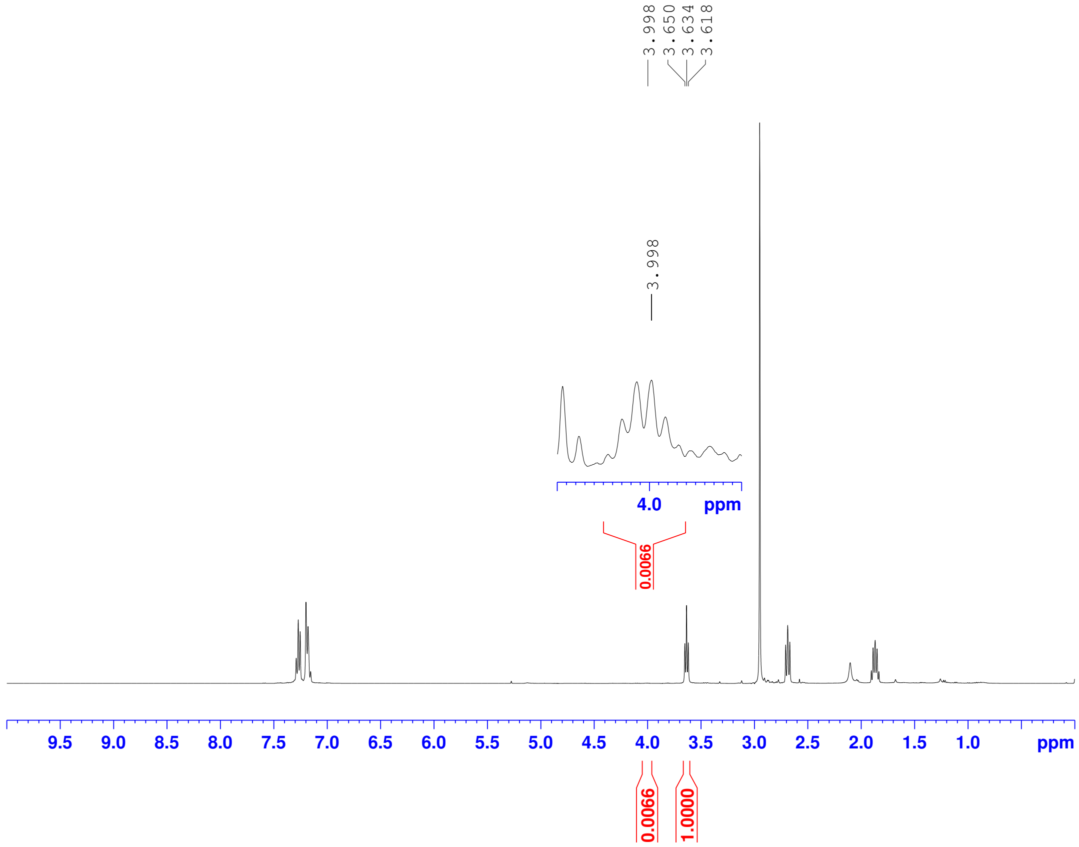

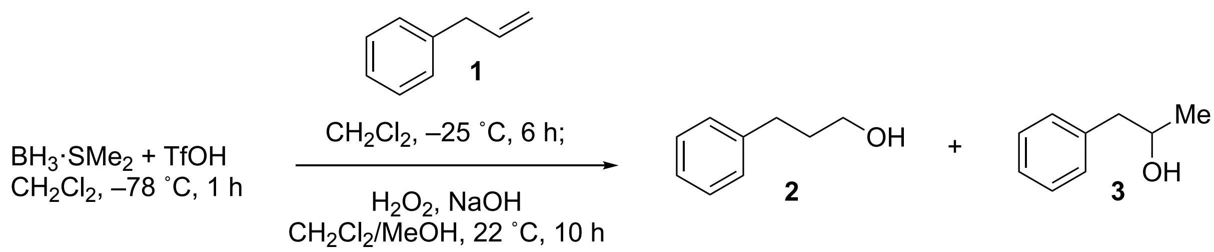

($^{13}C\{^1H\}$ NMR, 100 MHz, $CDCl_3$)

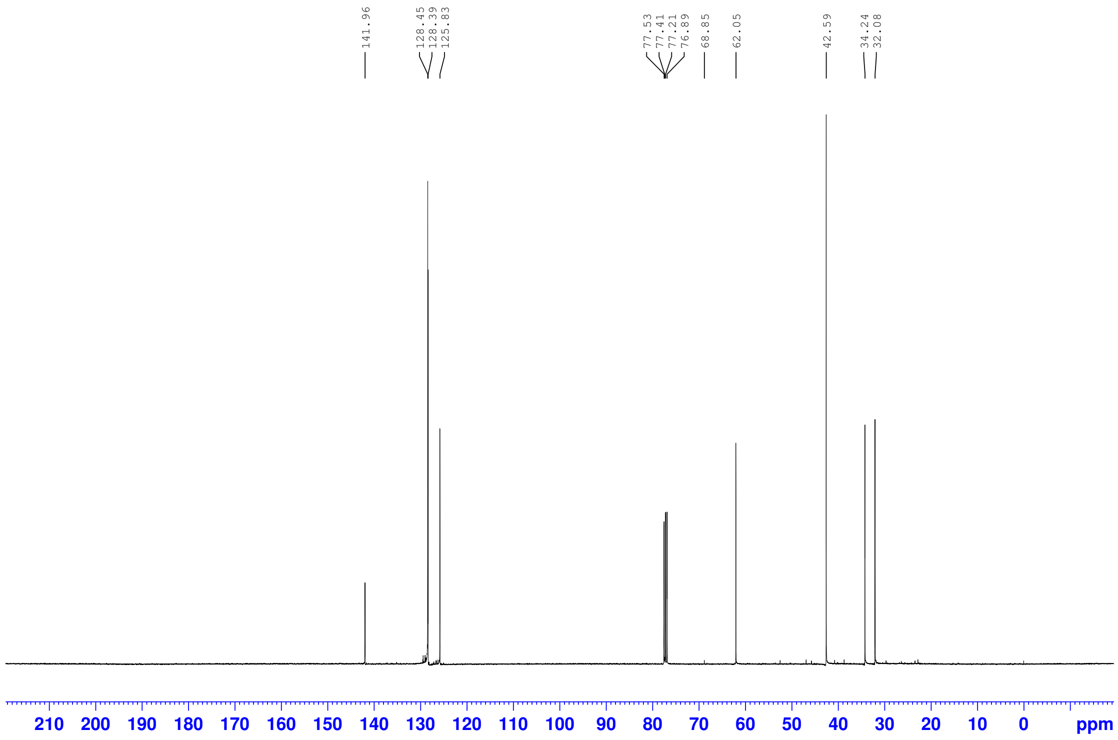

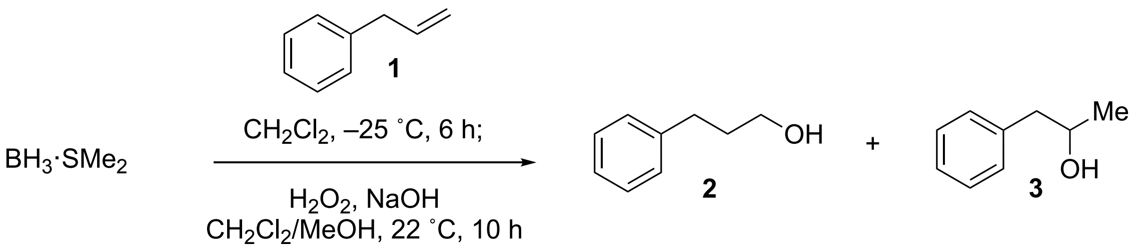

(Single-Pulse ^{1}H NMR, 400 MHz, $CDCl_3$)

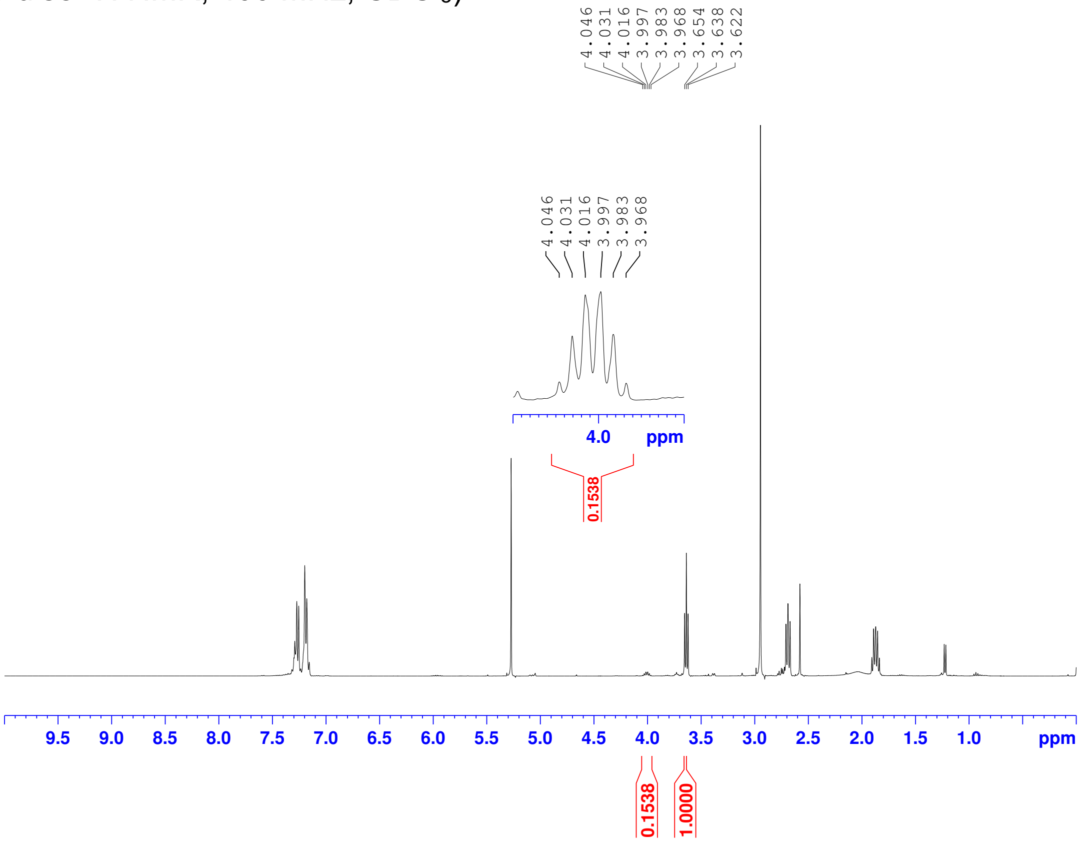

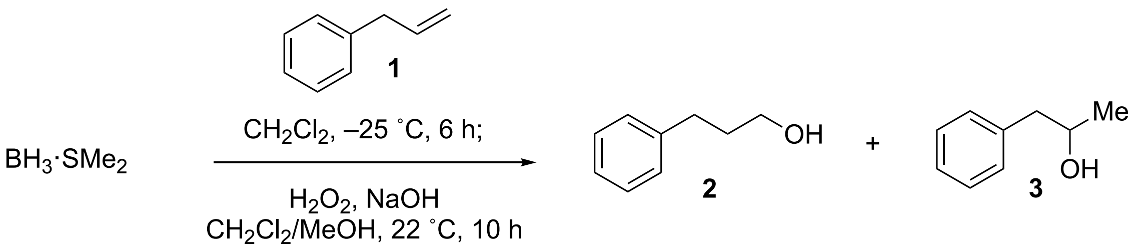

(^{13}C{^{1}H} NMR, 100 MHz, $CDCl_3$)

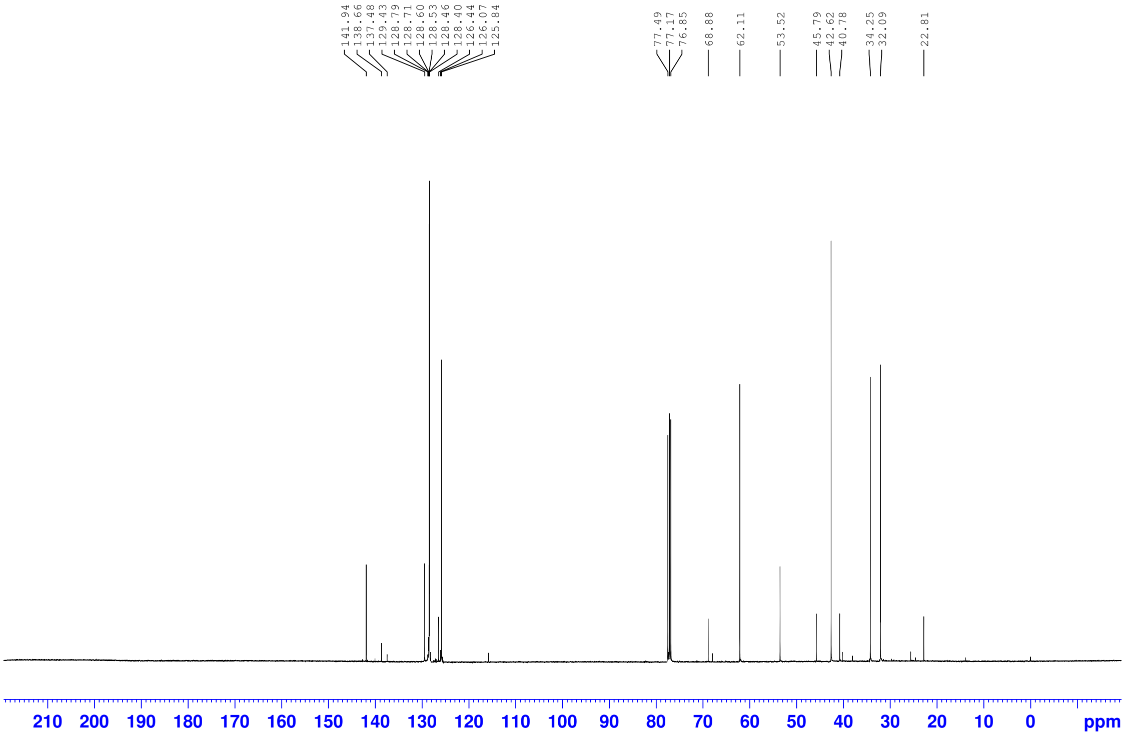

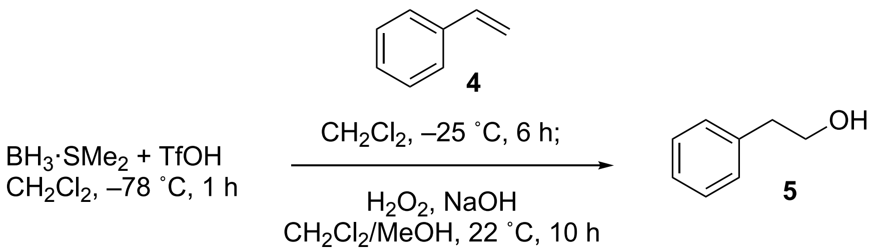

(Single-Pulse ^{1}H NMR, 400 MHz, $CDCl_3$)

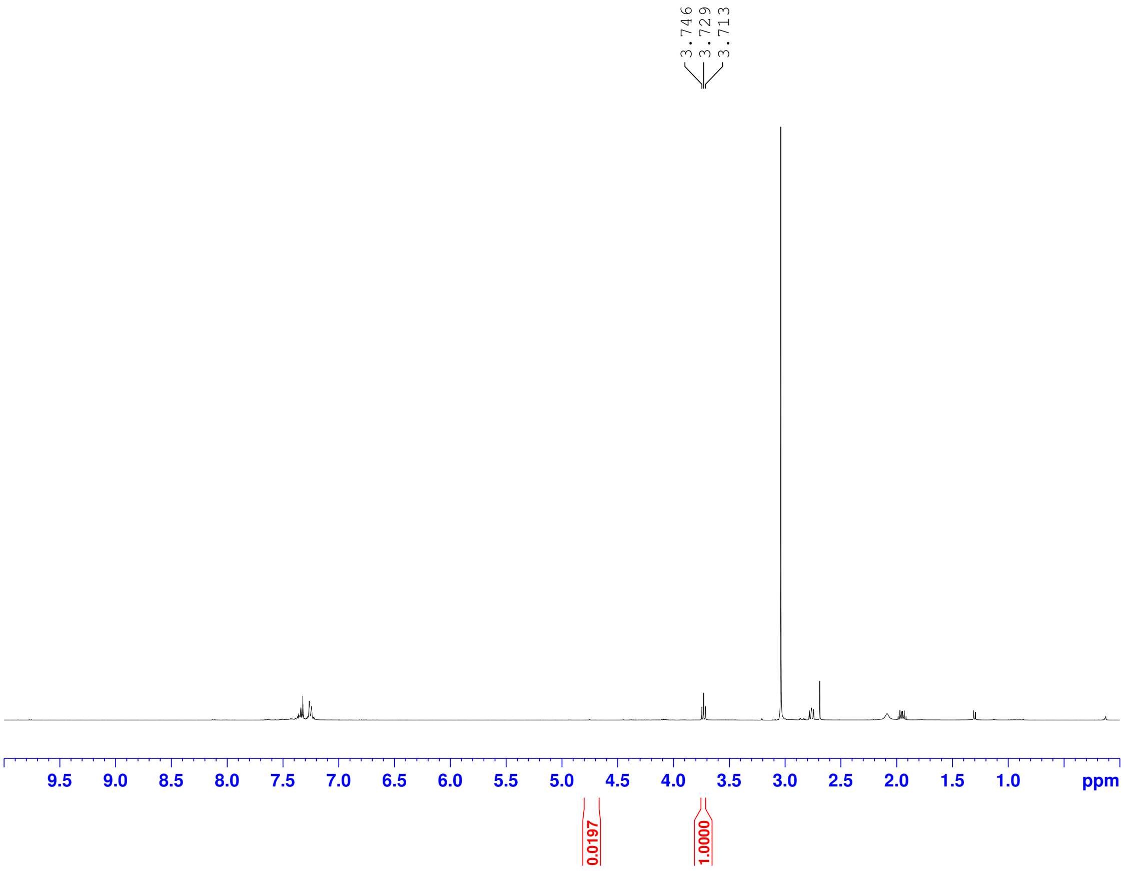

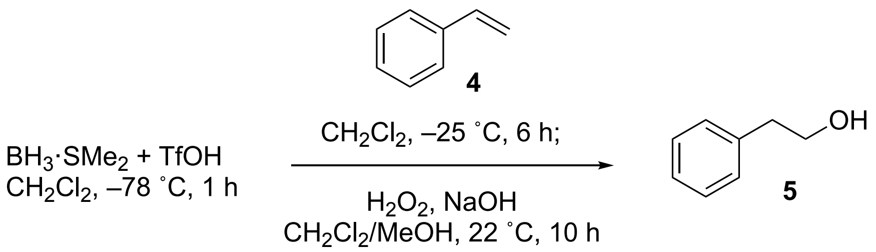

($^{13}C\{^1H\}$ NMR, 100 MHz, $CDCl_3$)

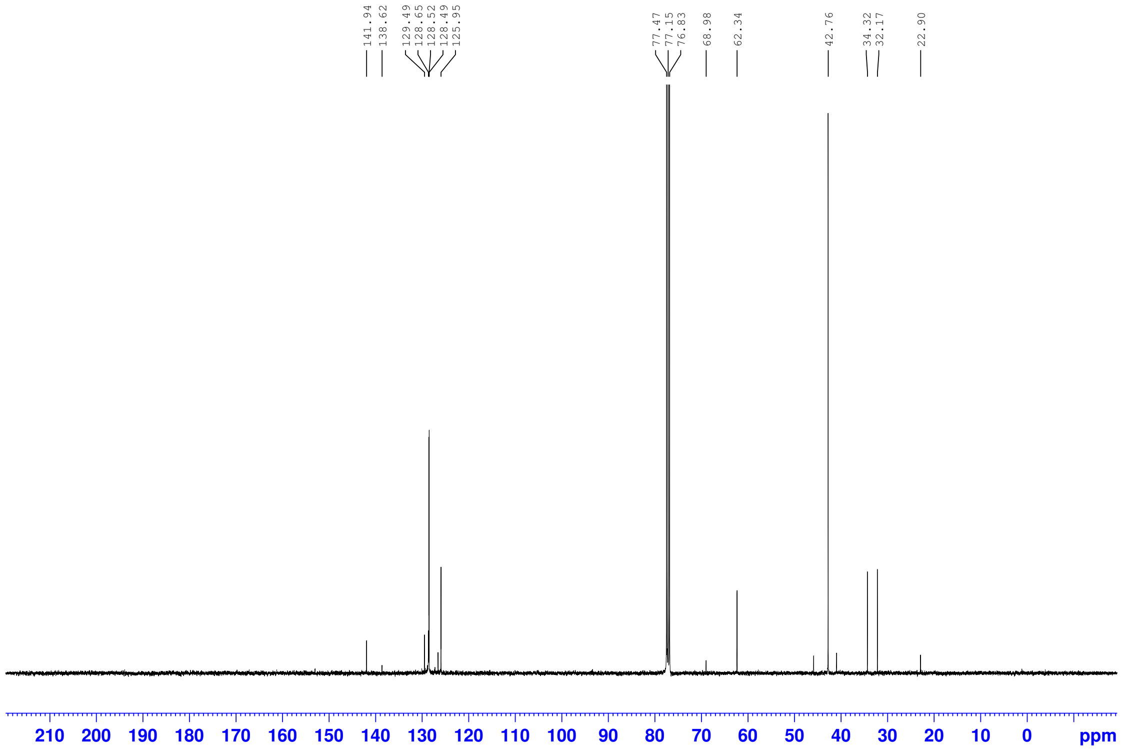

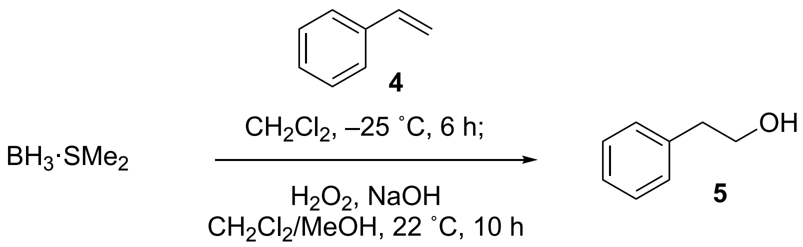

(Single-Pulse ^{1}H NMR, 400 MHz, $CDCl_3$)

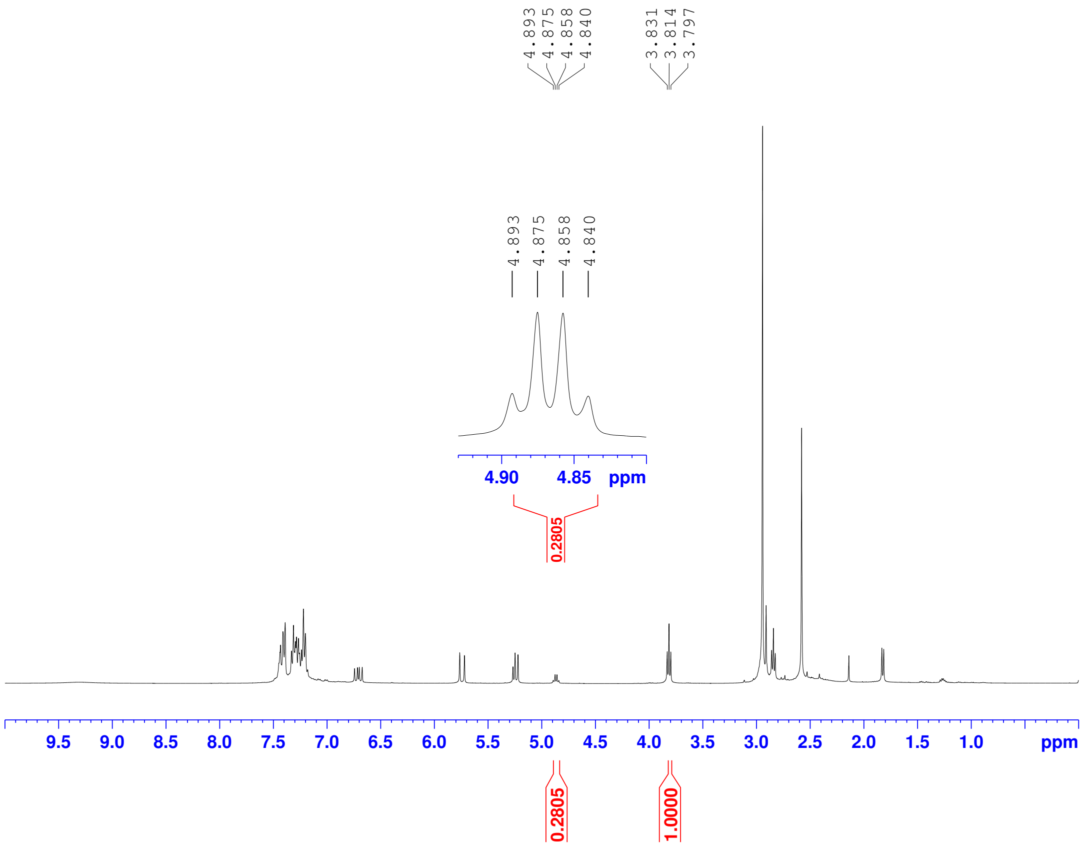

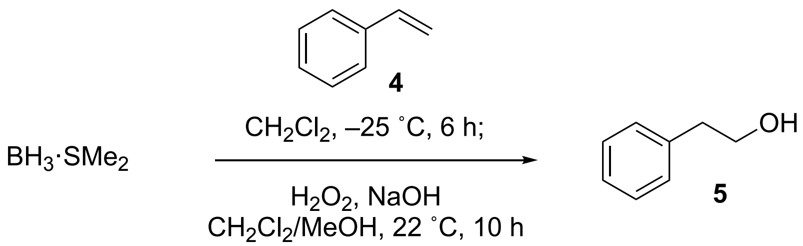

($^{13}C\{^{1}H\}$ NMR, 100 MHz, $CDCl_3$)

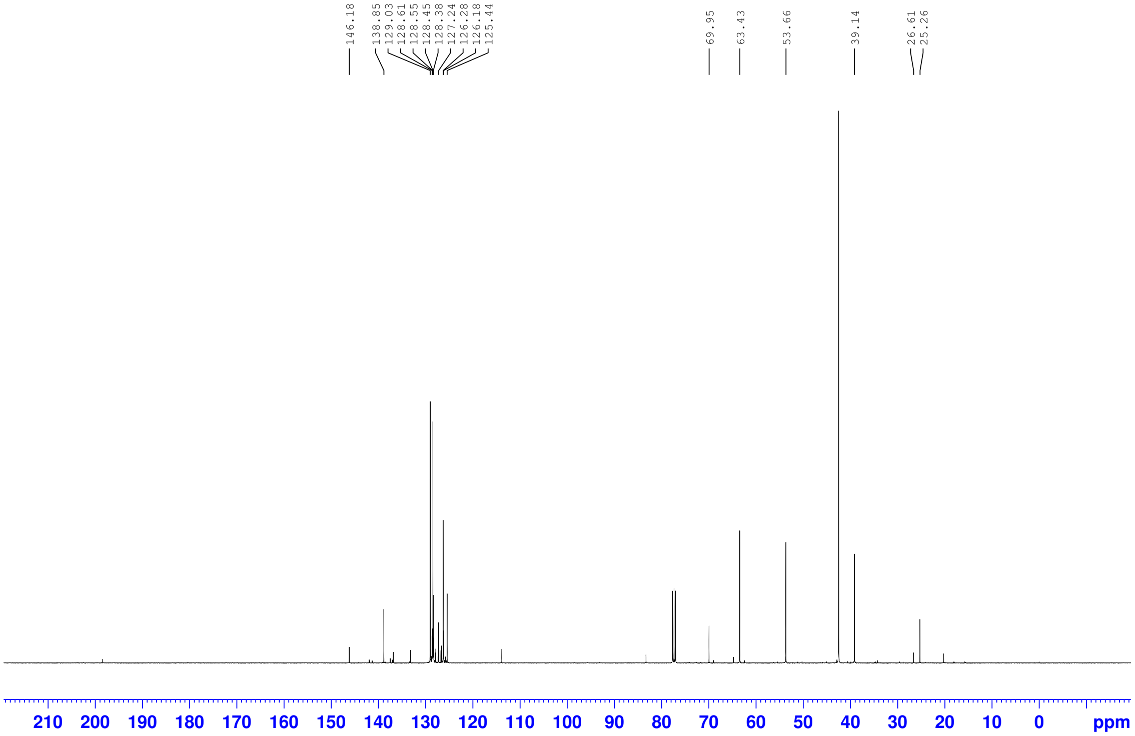

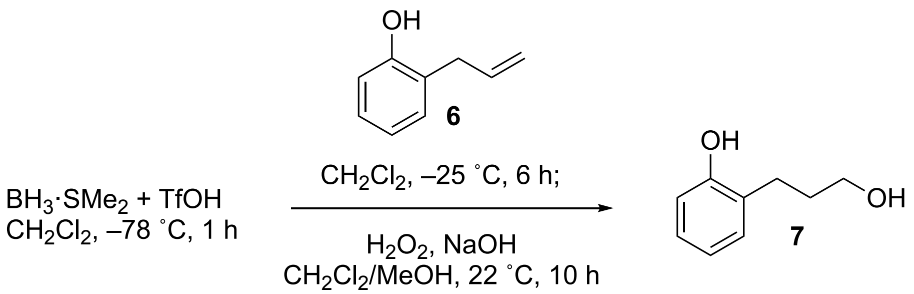

(Single-Pulse ¹H NMR, 400 MHz, CDCl₃)

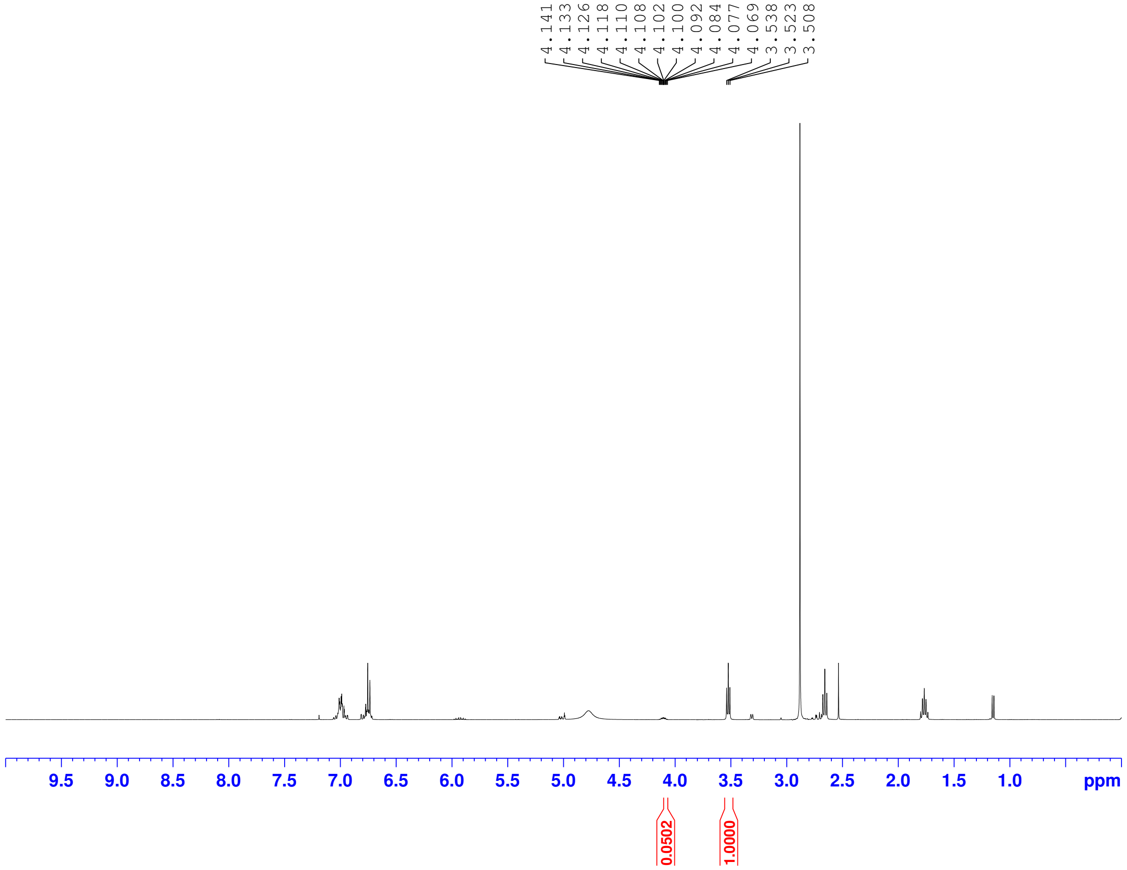

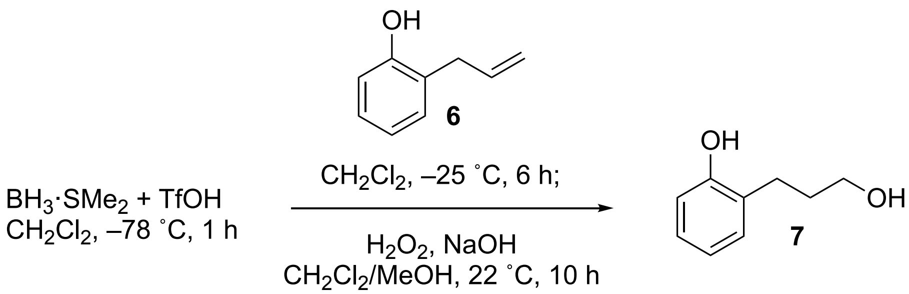

($^{13}C\{^1H\}$ NMR, 100 MHz, $CDCl_3$)

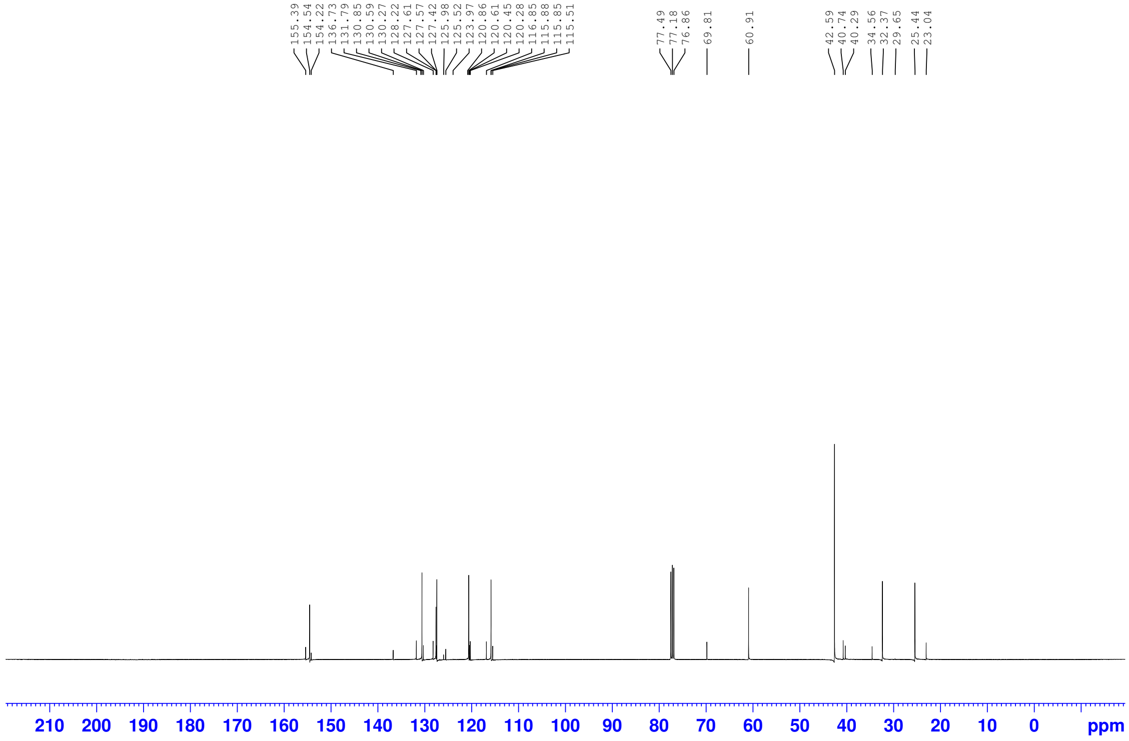

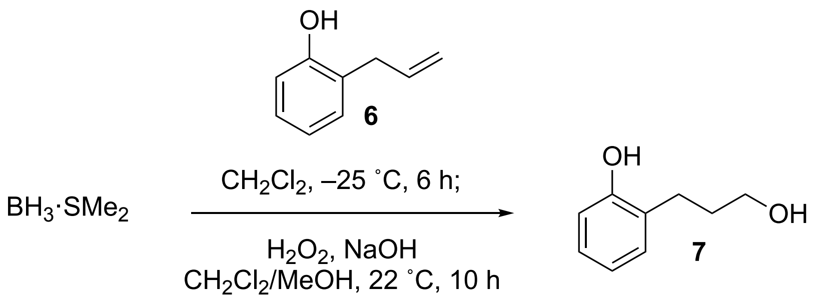

(Single-Pulse ^{1}H NMR, 400 MHz, $CDCl_3$)

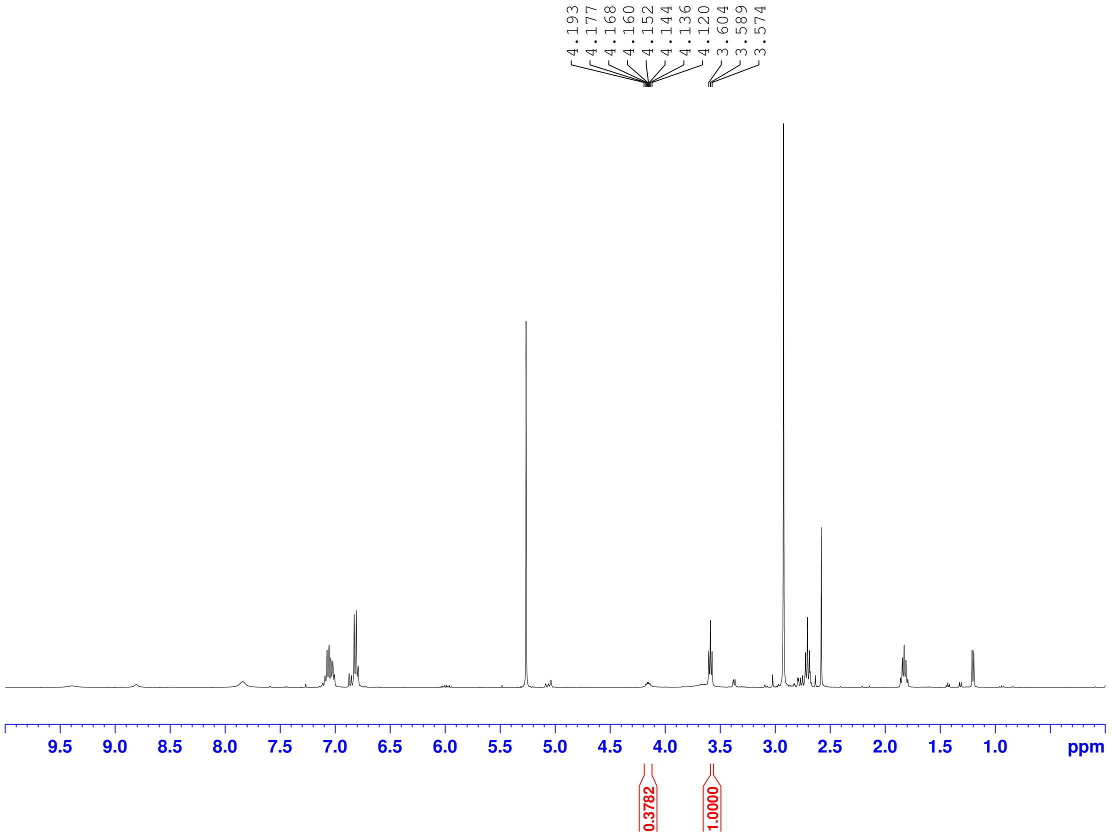

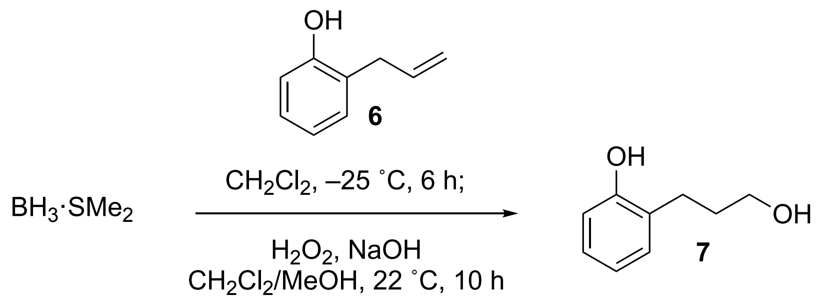

($^{13}C\{^{1}H\}$ NMR, 100 MHz, $CDCl_3$)

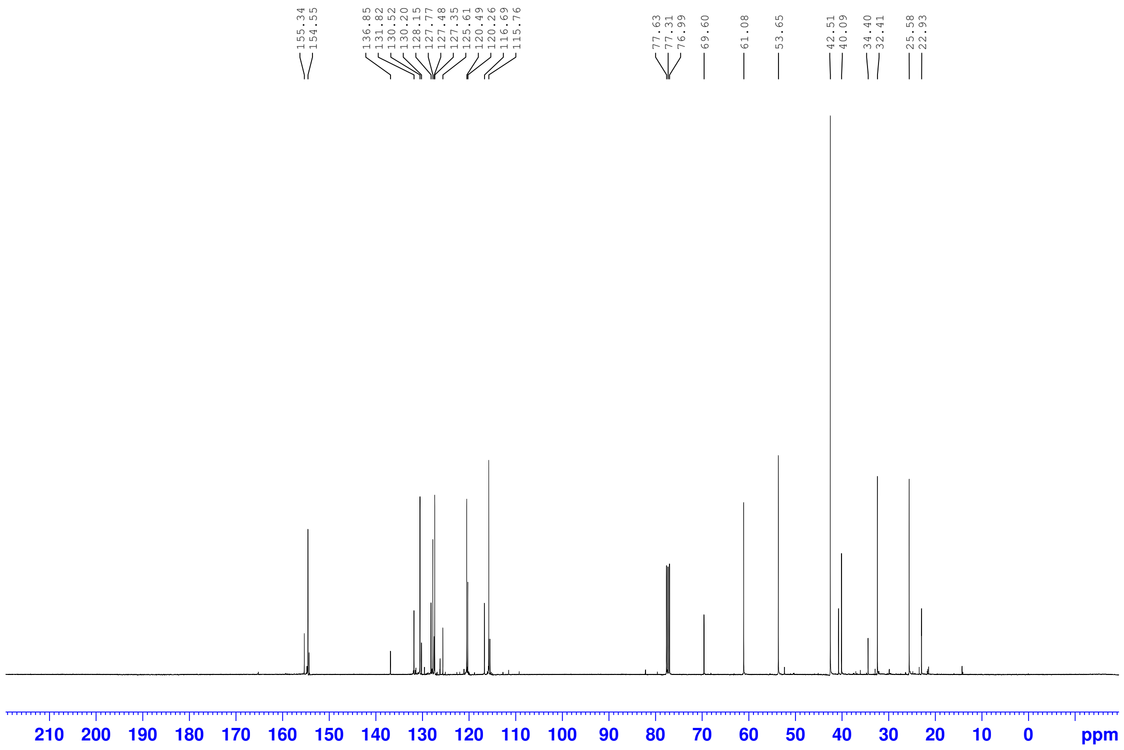

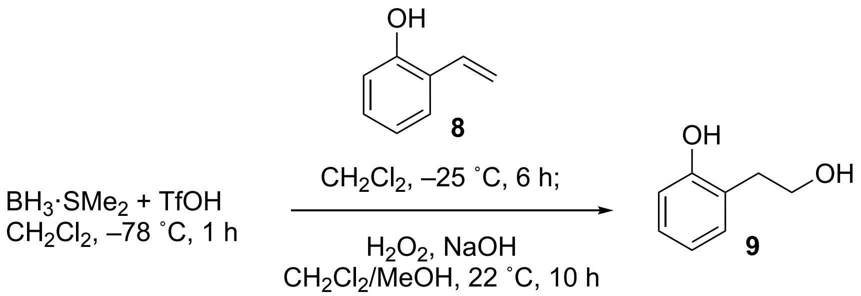

(^{13}C{^{1}H} NMR, 100 MHz, $CDCl_3$)

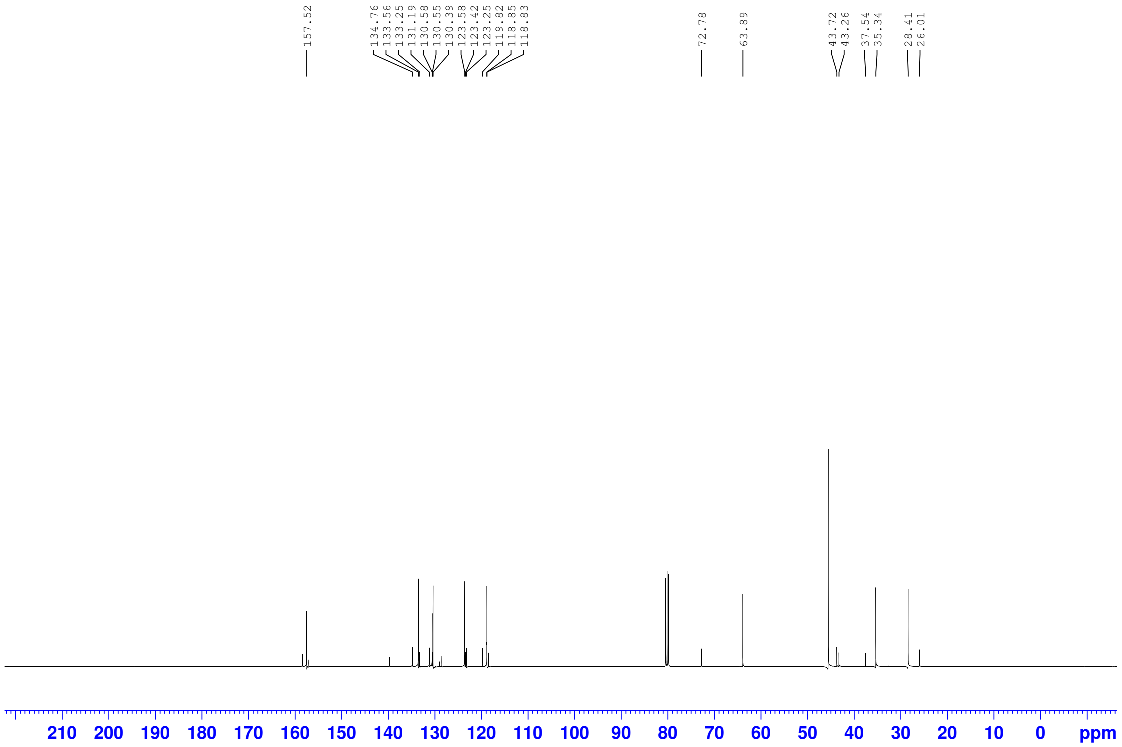

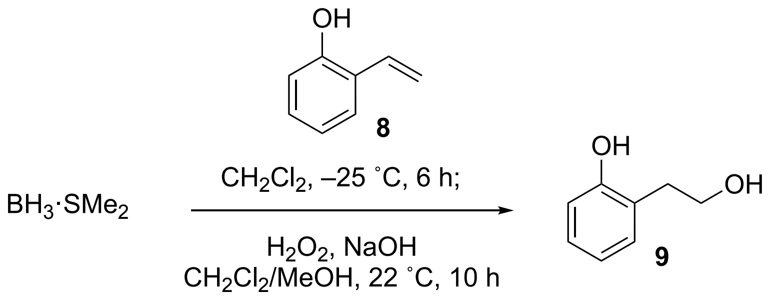

($^{13}C\{^{1}H\}$ NMR, 100 MHz, $CDCl_3$)

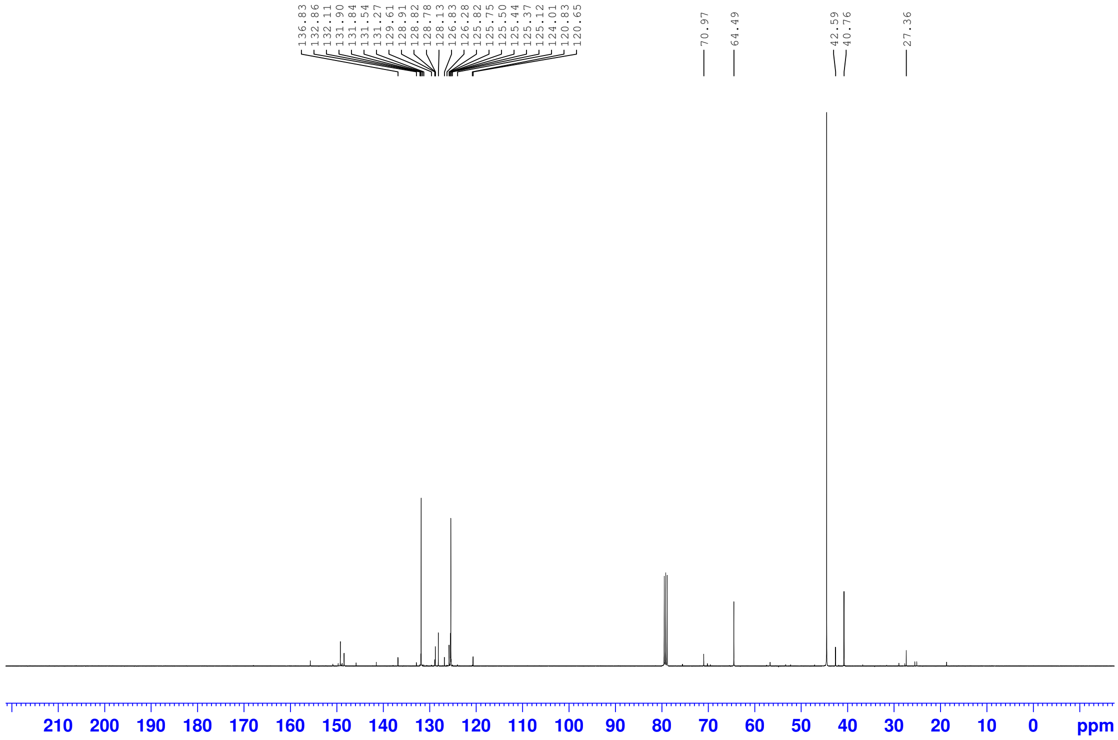

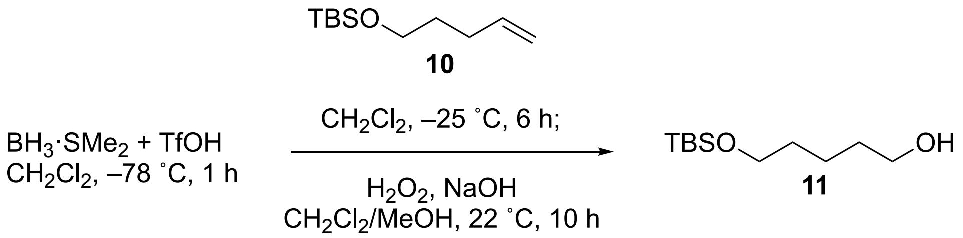

(Single-Pulse ^{1}H NMR, 400 MHz, $CDCl_3$)

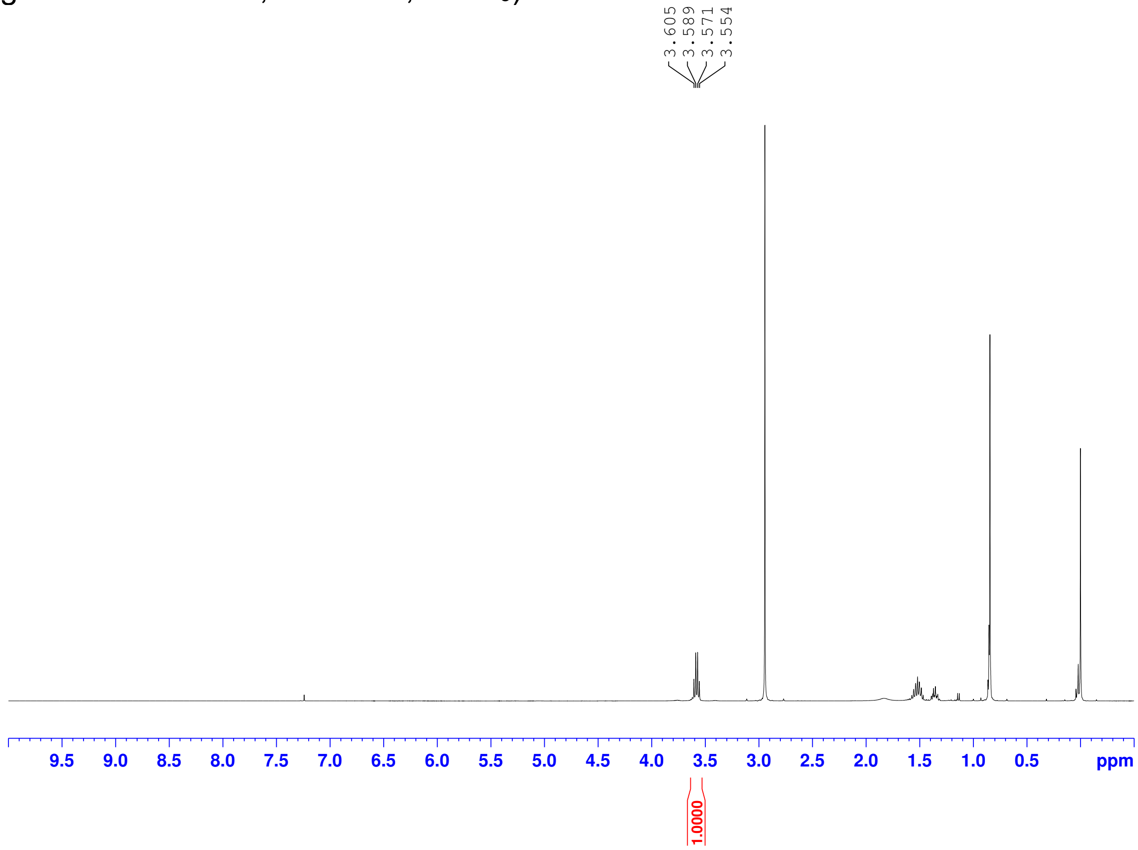

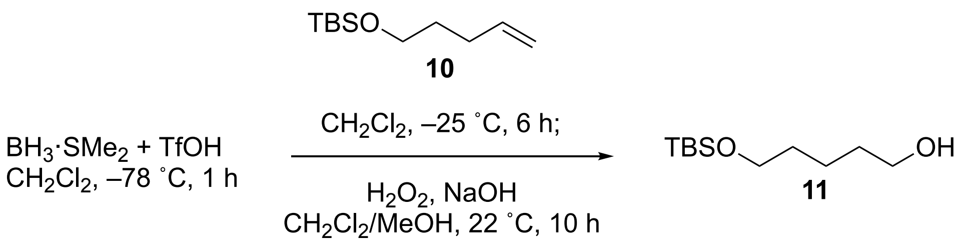

($^{13}C\{^{1}H\}$ NMR, 100 MHz, $CDCl_3$)

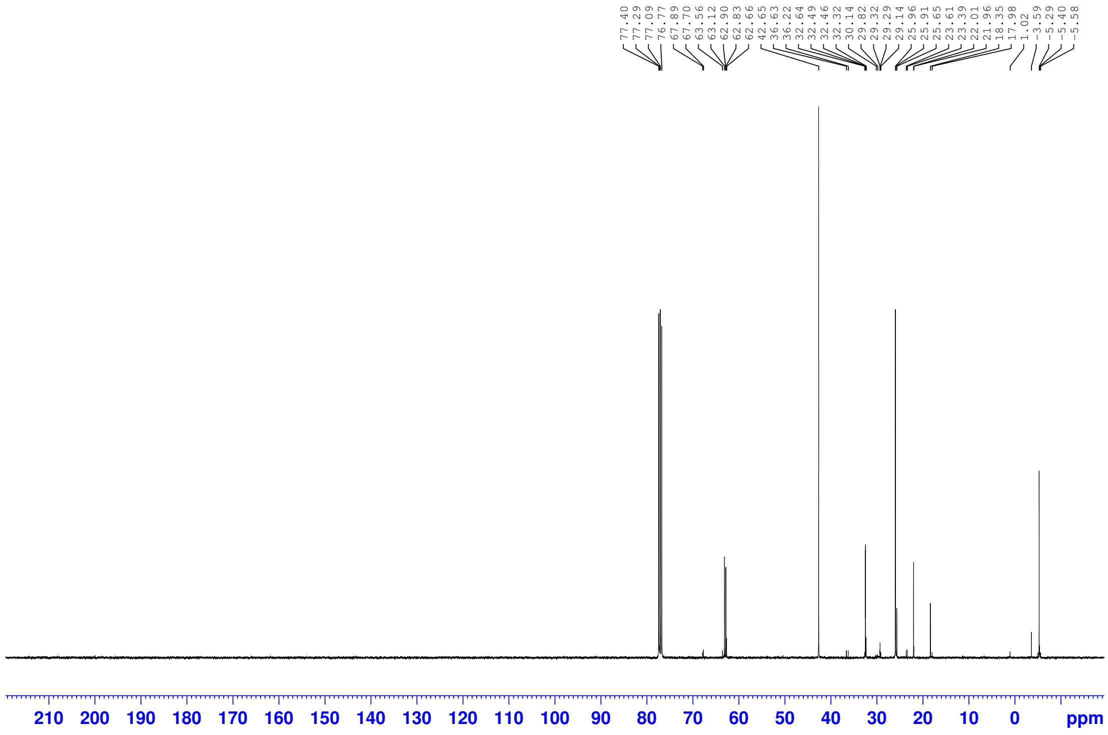

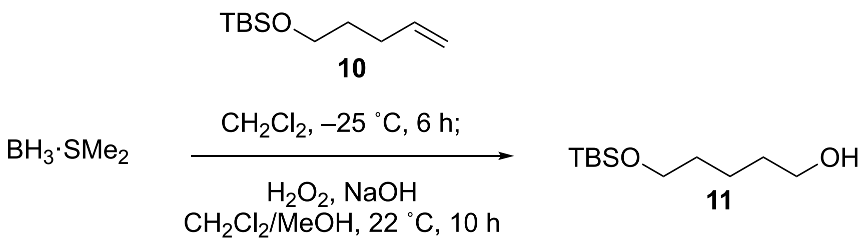

(Single-Pulse ^{1}H NMR, 400 MHz, $CDCl_3$)

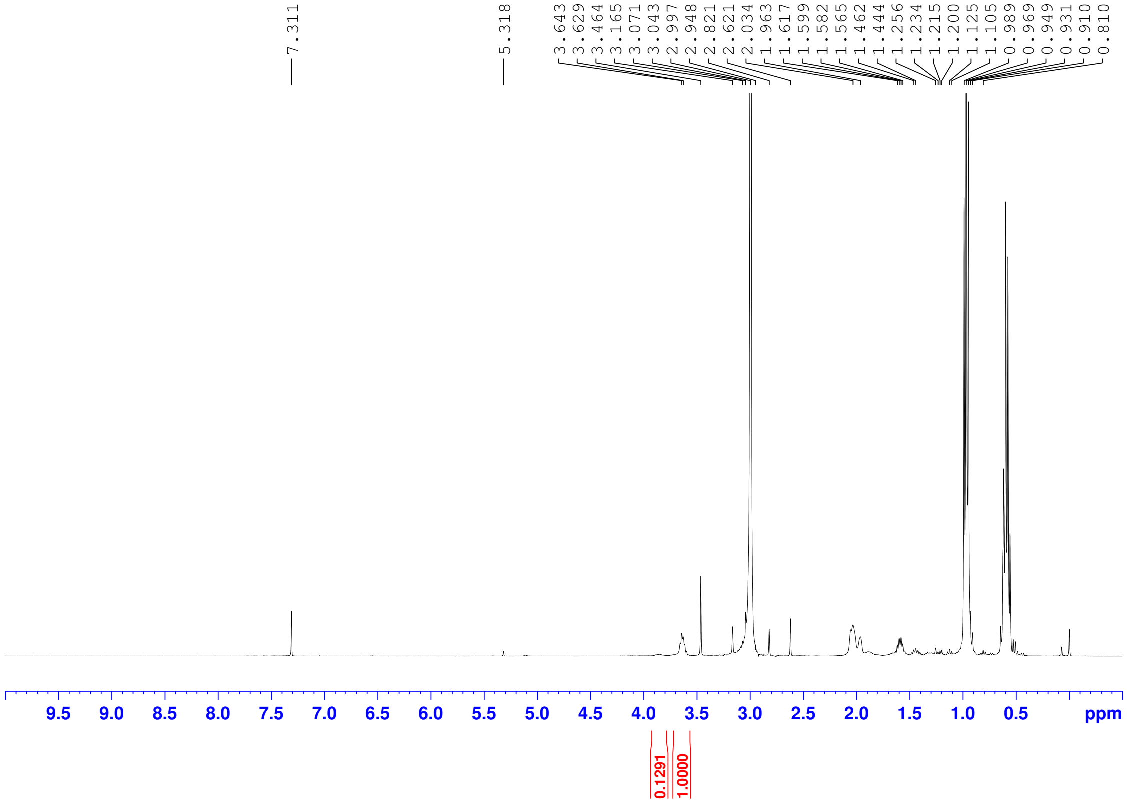

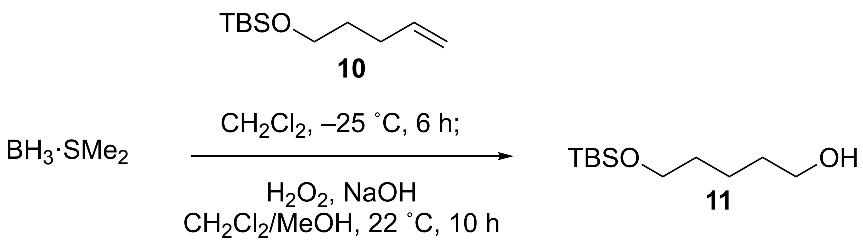

(^{13}C{^{1}H} NMR, 100 MHz, $CDCl_3$)

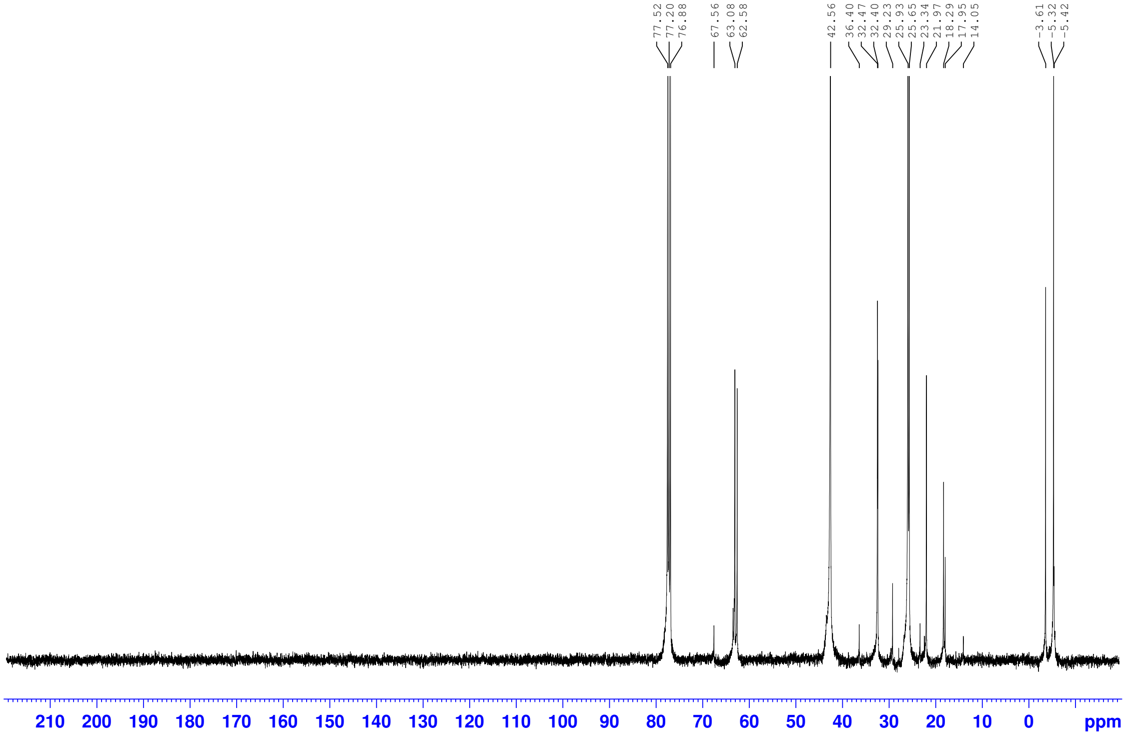

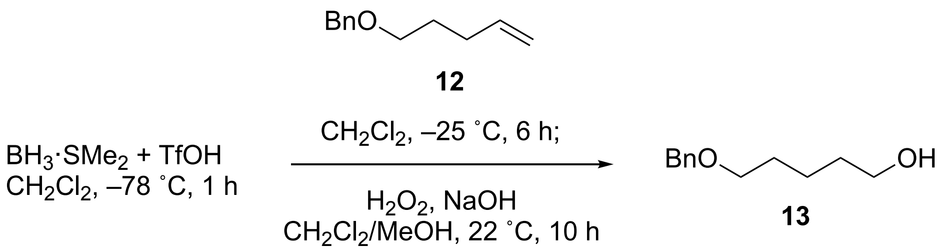

(Single-Pulse ^{1}H NMR, 400 MHz, $CDCl_3$)

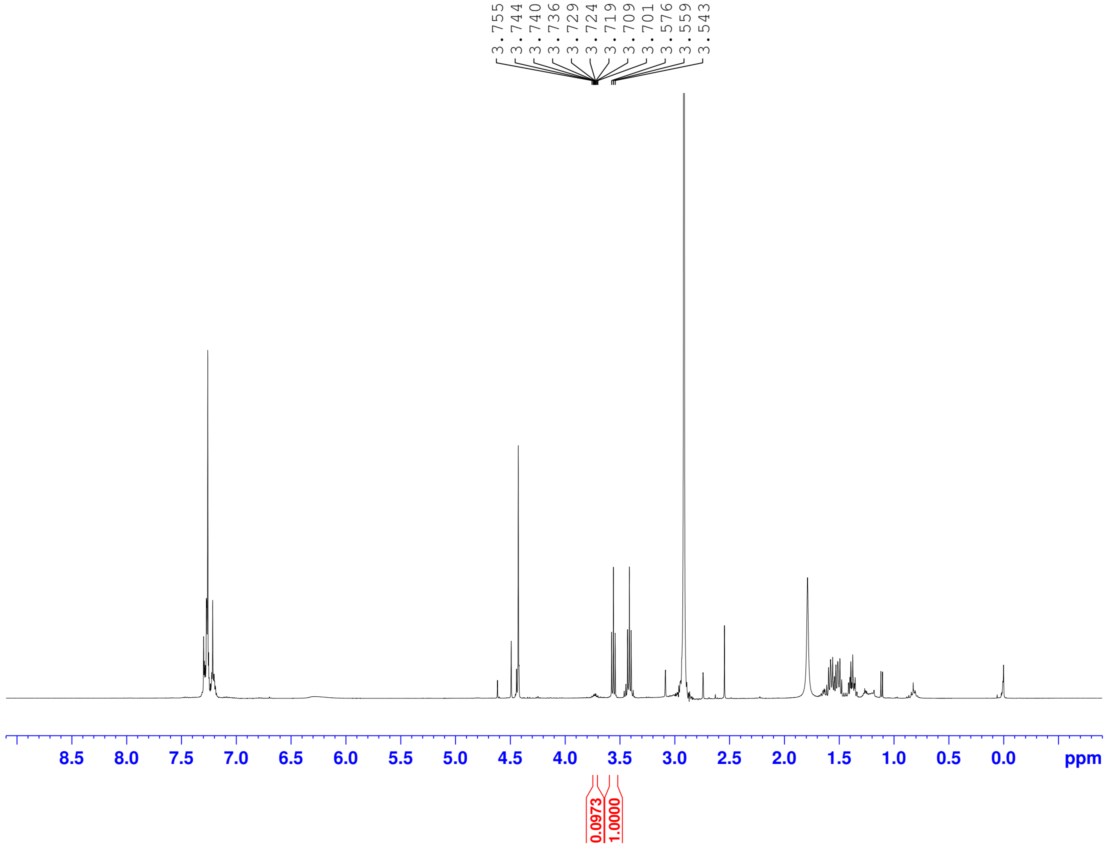

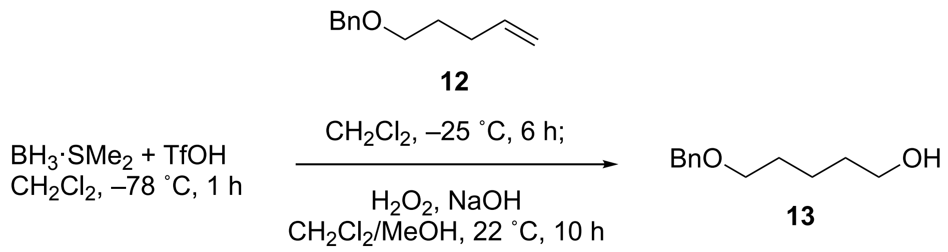

(^{13}C{^{1}H} NMR, 100 MHz, $CDCl_3$)

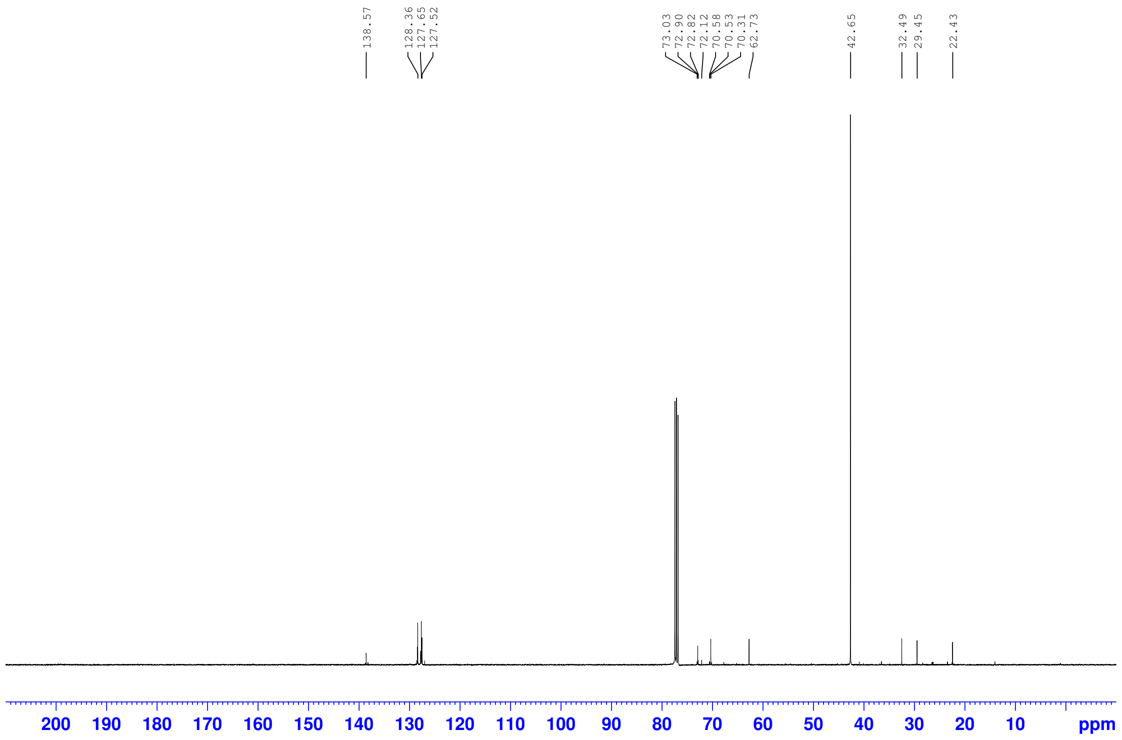

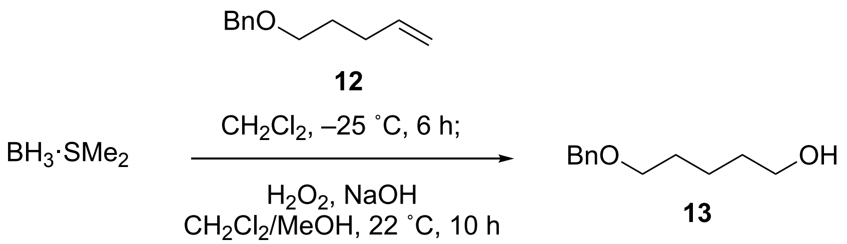

(Single-Pulse ^{1}H NMR, 400 MHz, $CDCl_3$)

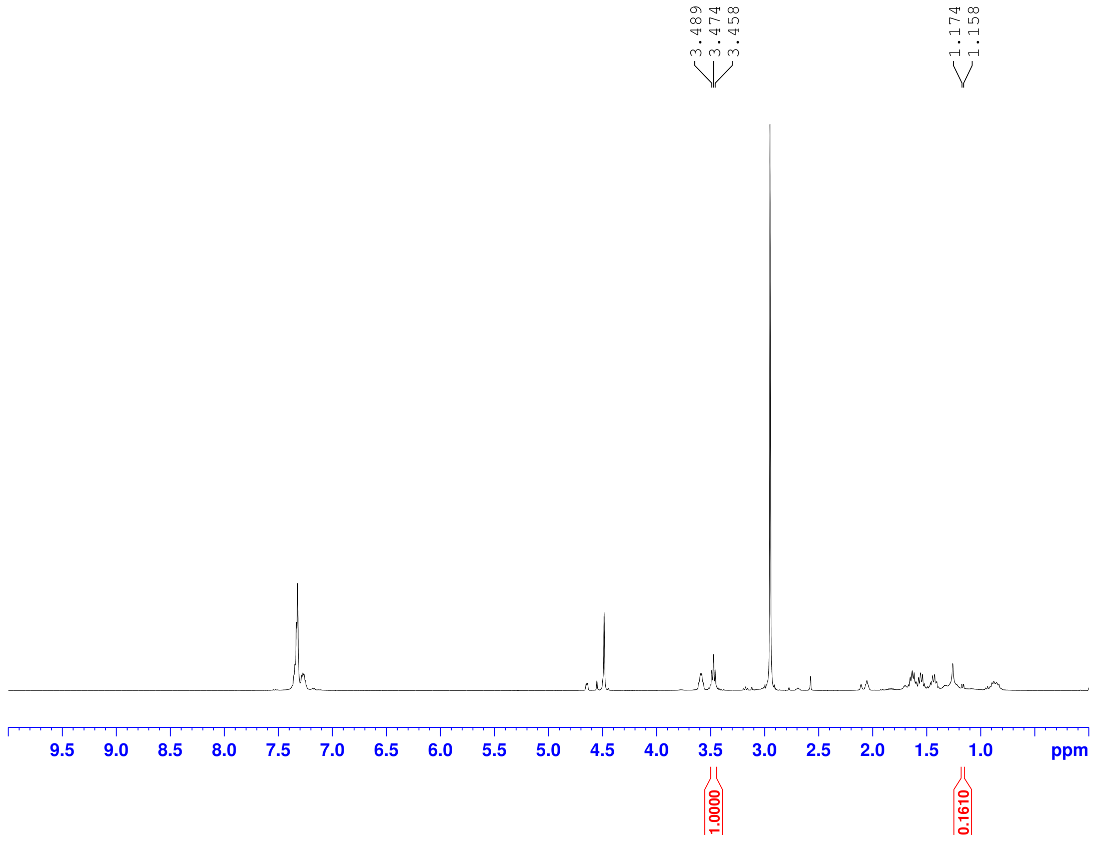

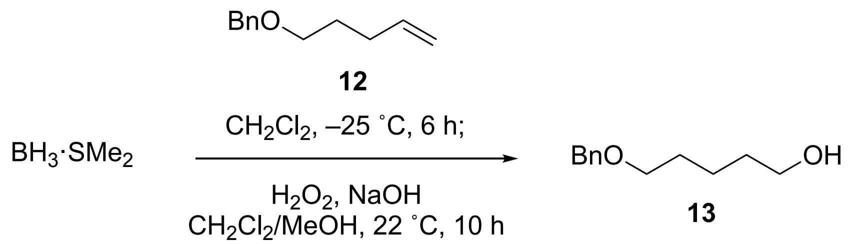

(^{13}C{^{1}H} NMR, 100 MHz, $CDCl_3$)

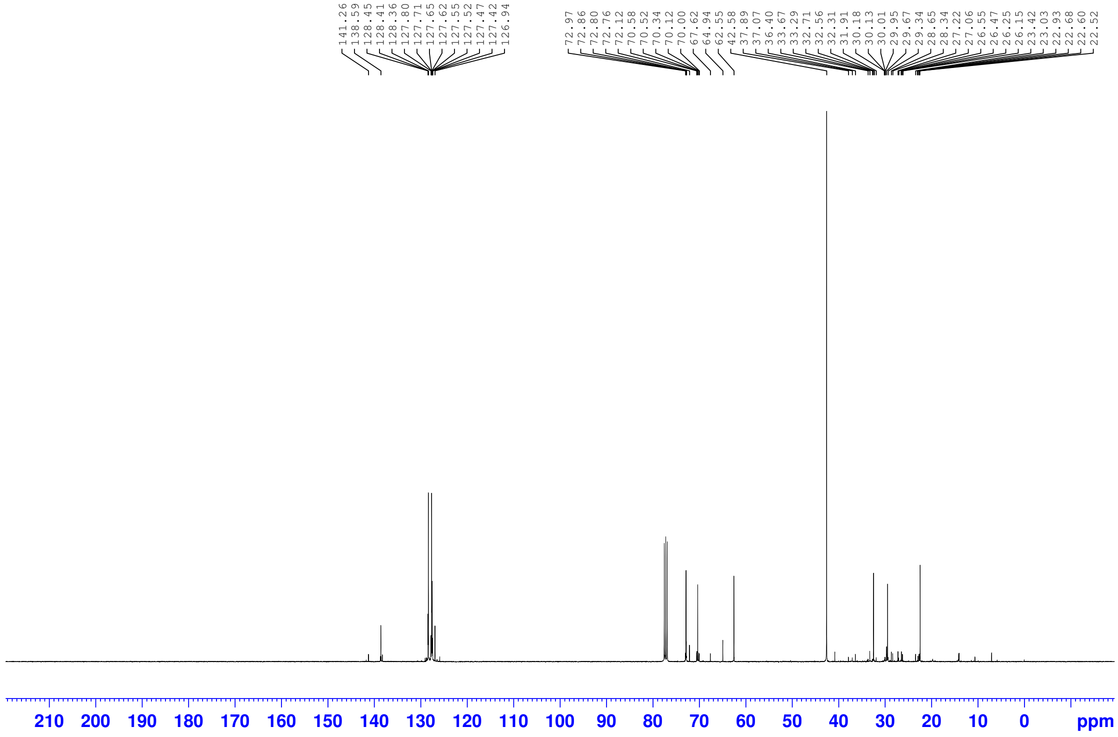

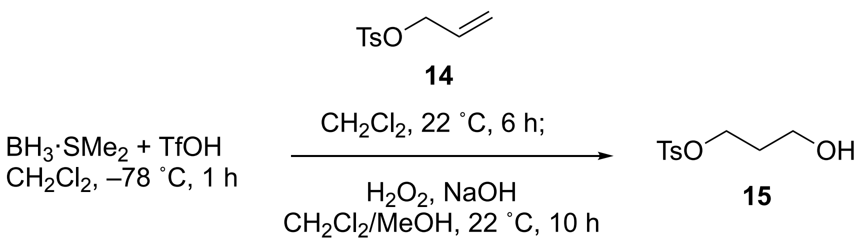

(Single-Pulse ^1H NMR, 400 MHz, $CDCl_3$)

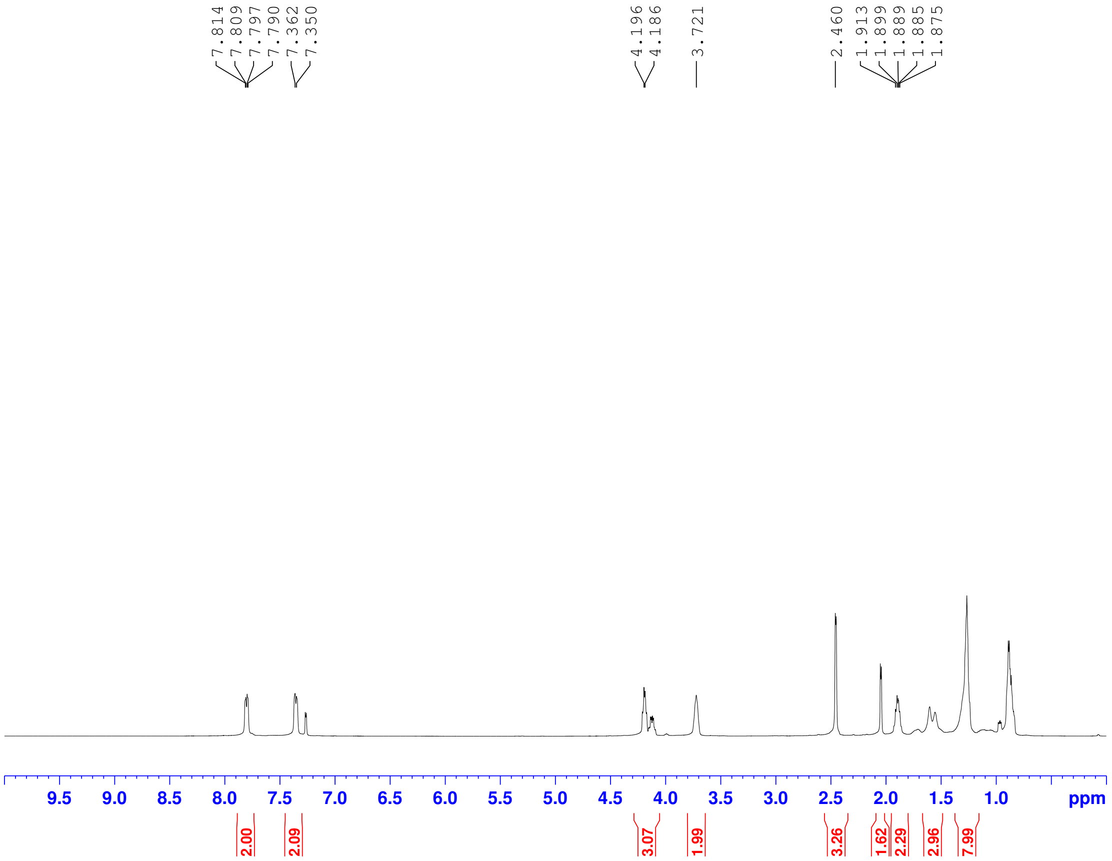

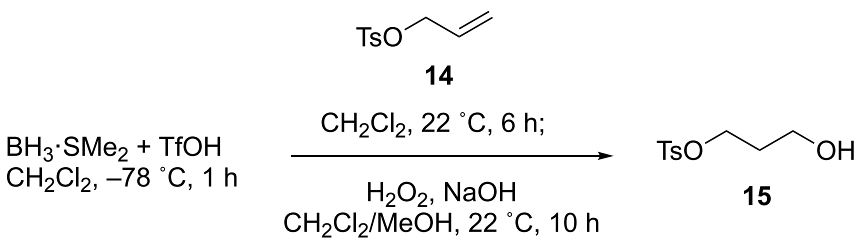

($^{13}C\{^1H\}$ NMR, 100 MHz, $CDCl_3$)

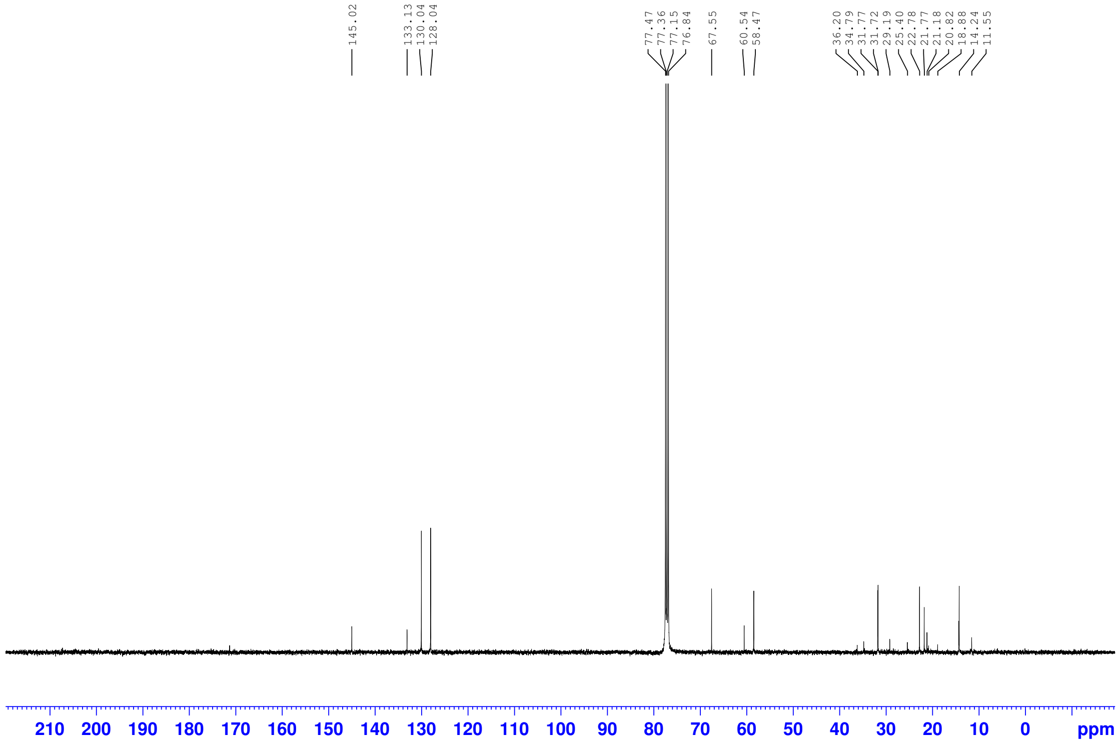

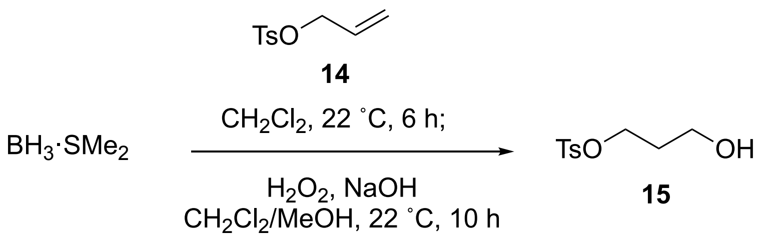

(Single-Pulse ^{1}H NMR, 400 MHz, $CDCl_3$)

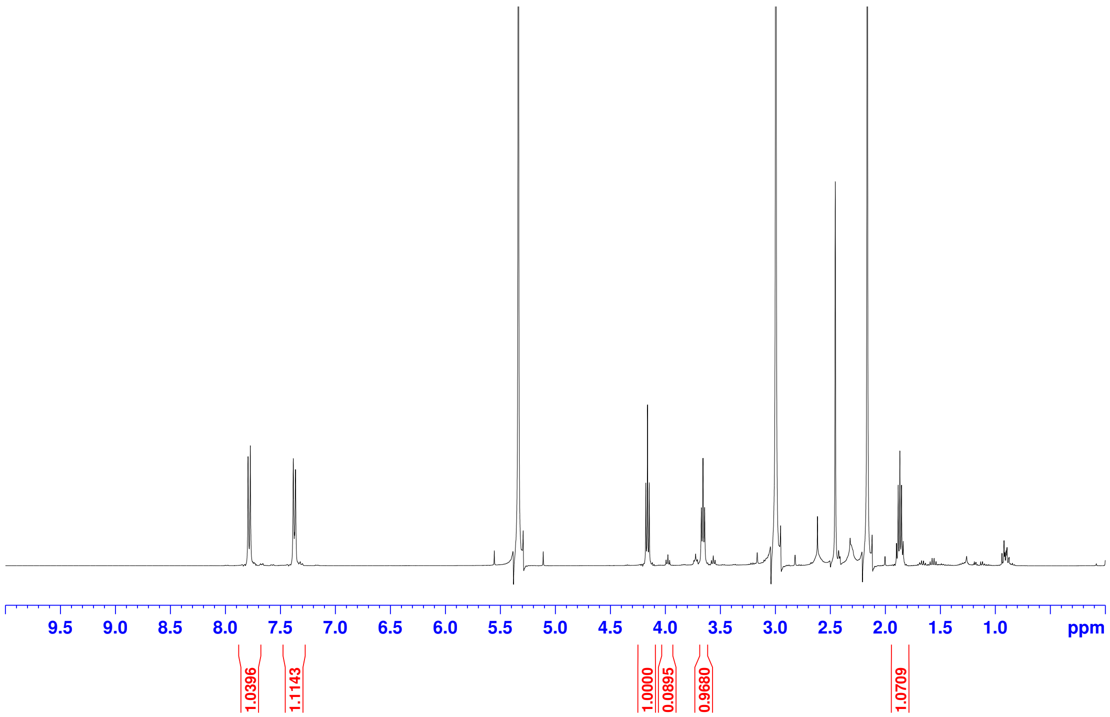

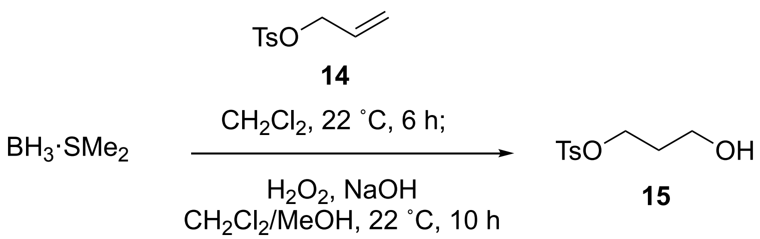

(^{13}C{^{1}H} NMR, 100 MHz, CDCl$_3$)

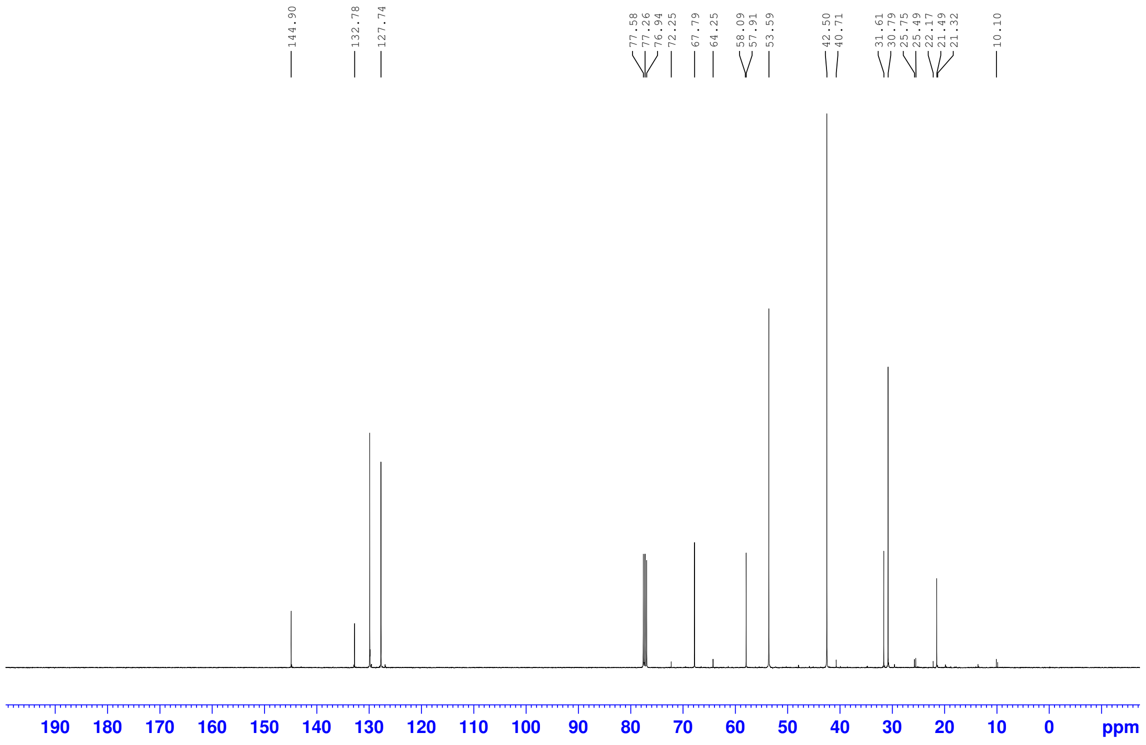

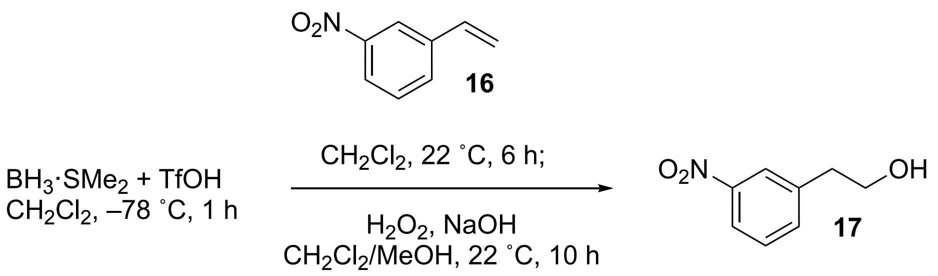

(Single-Pulse ^{1}H NMR, 400 MHz, $CDCl_3$)

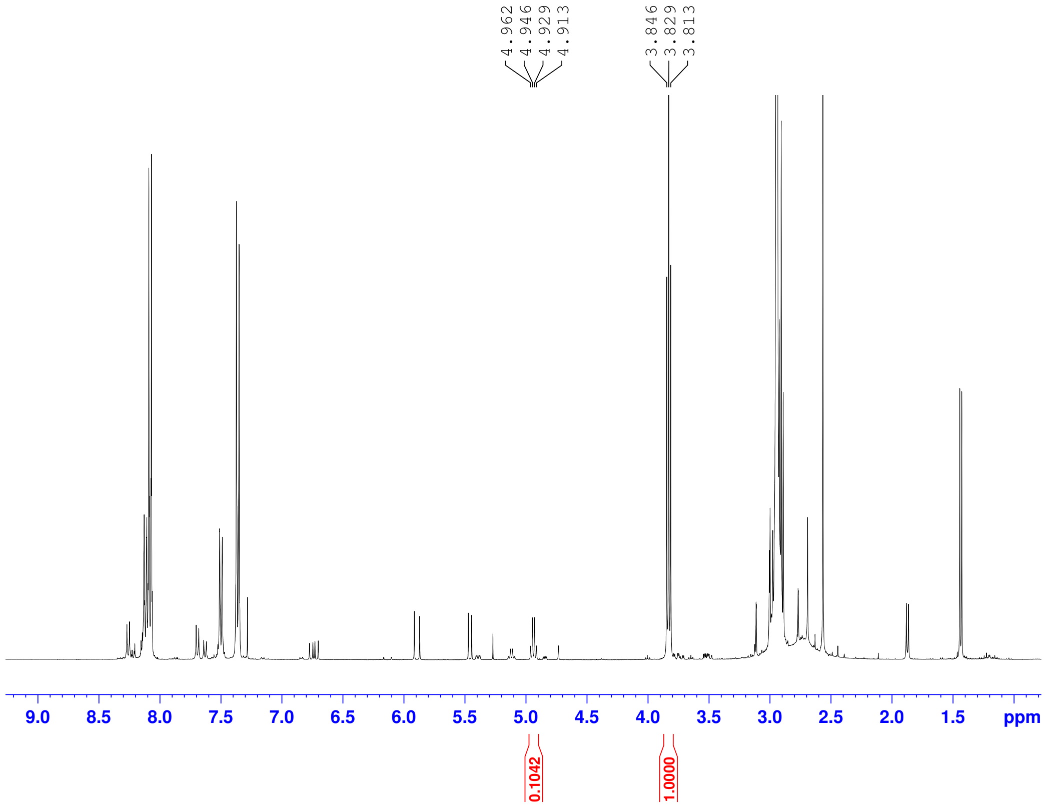

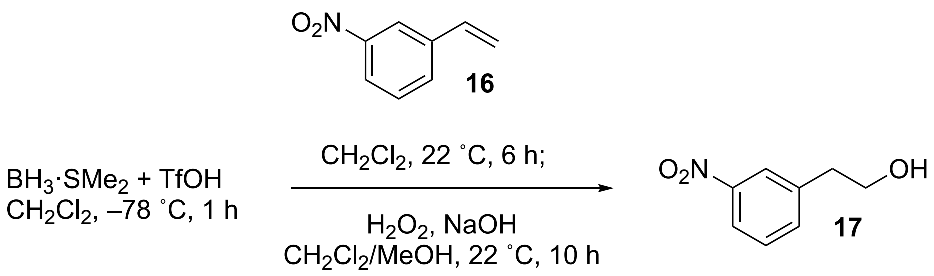

($^{13}C\{^1H\}$ NMR, 100 MHz, $CDCl_3$)

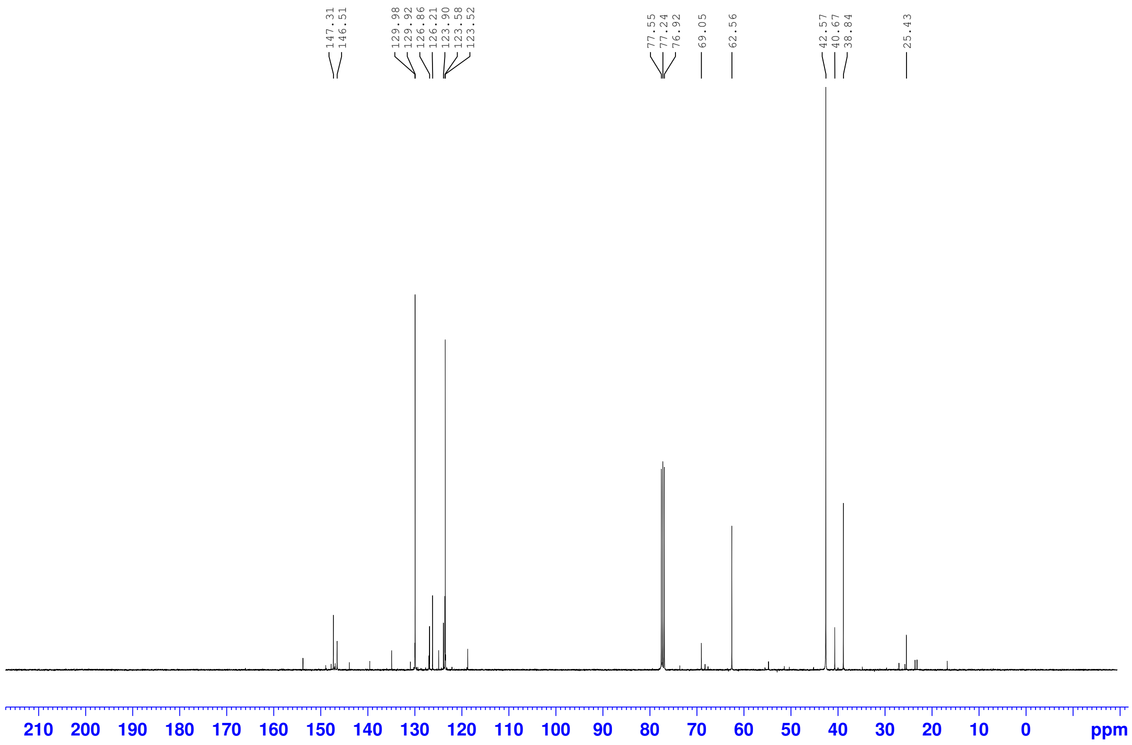

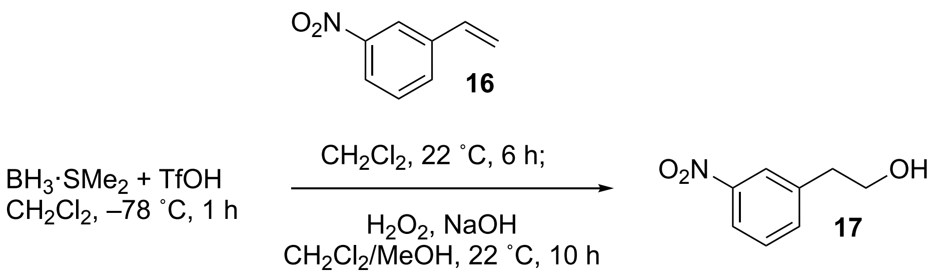

(Single-Pulse ^{1}H NMR, 400 MHz, $CDCl_3$)

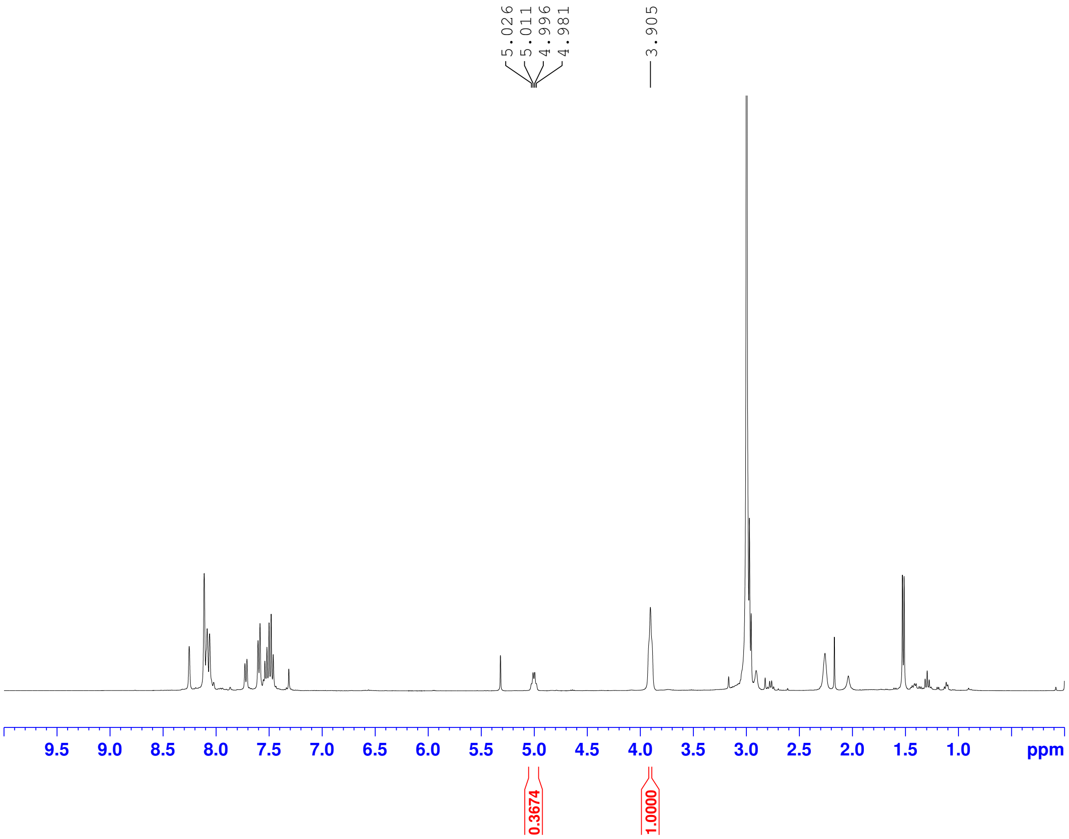

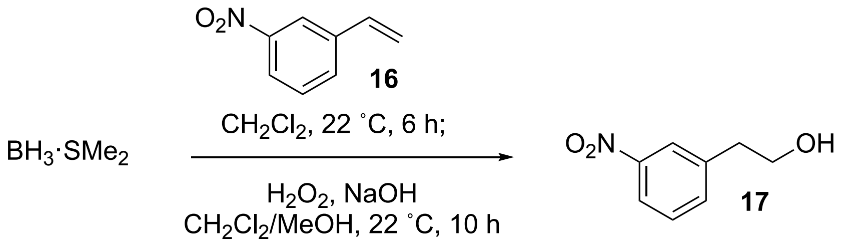

($^{13}C\{^{1}H\}$ NMR, 100 MHz, $CDCl_3$)

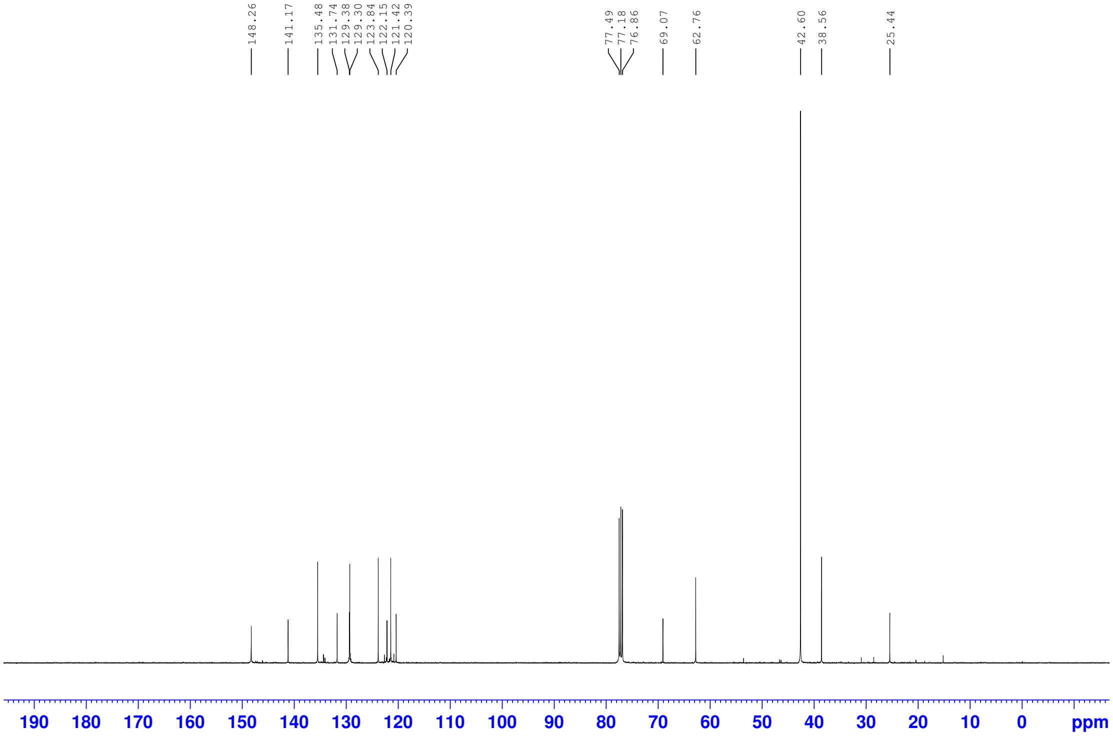

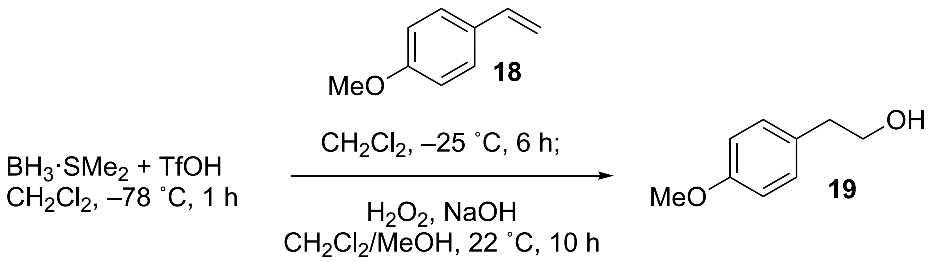

(Single-Pulse ^{1}H NMR, 400 MHz, CDCl$_3$)

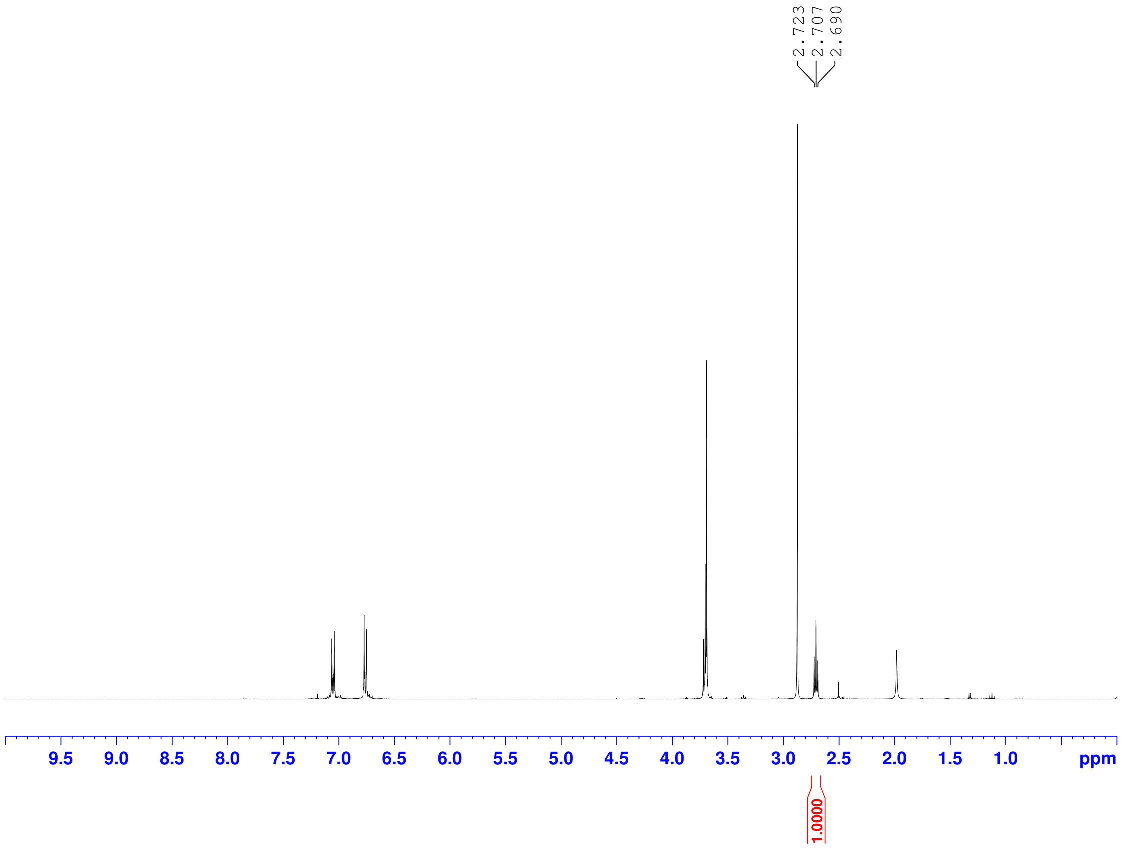

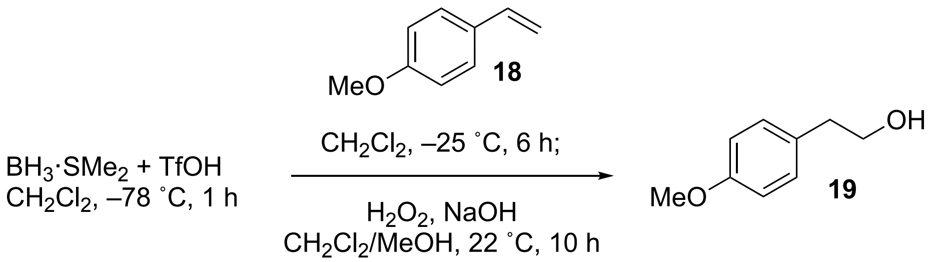

($^{13}C\{^{1}H\}$ NMR, 100 MHz, $CDCl_3$)

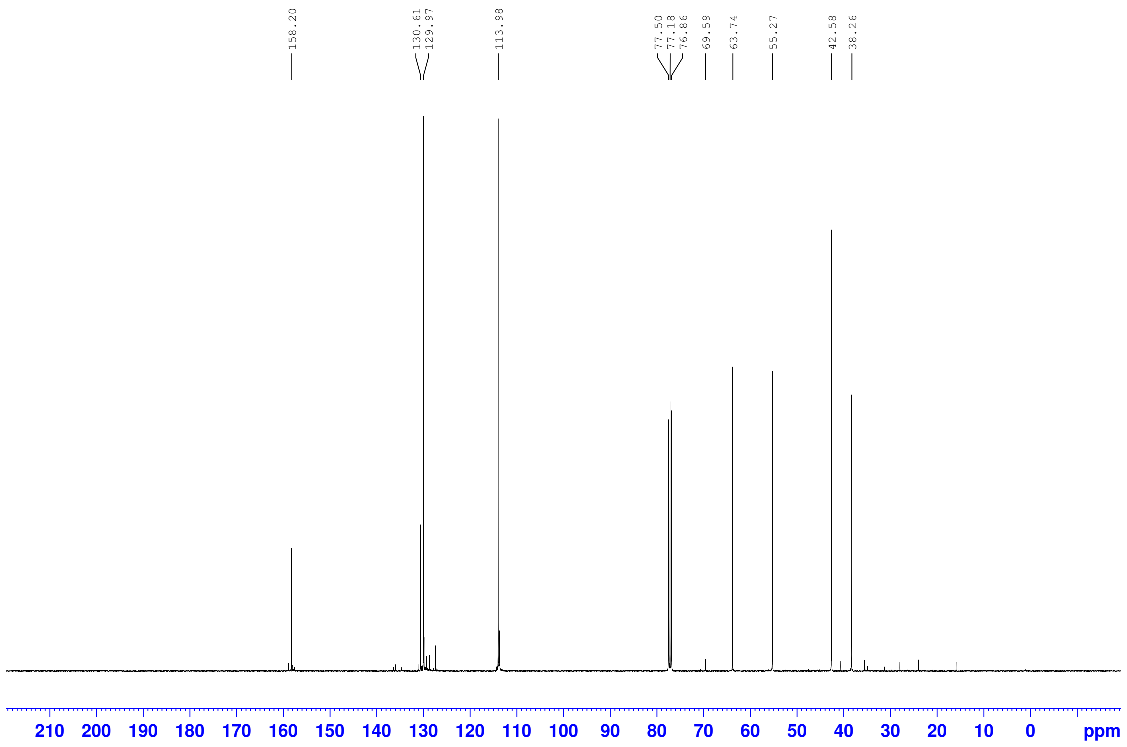

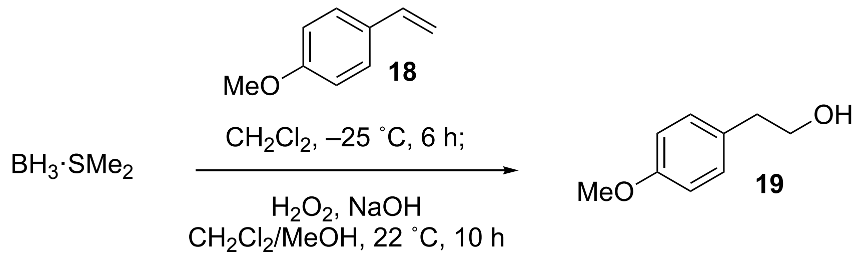

(Single-Pulse ^1H NMR, 400 MHz, $CDCl_3$)

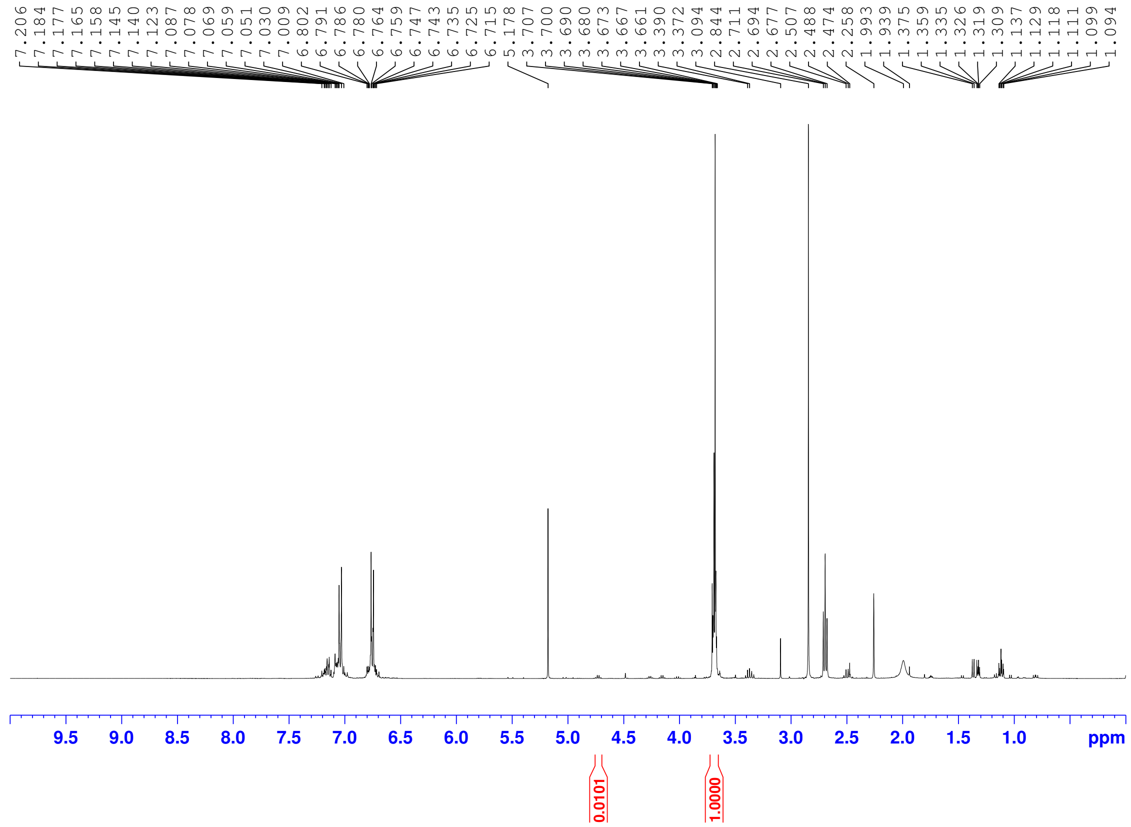

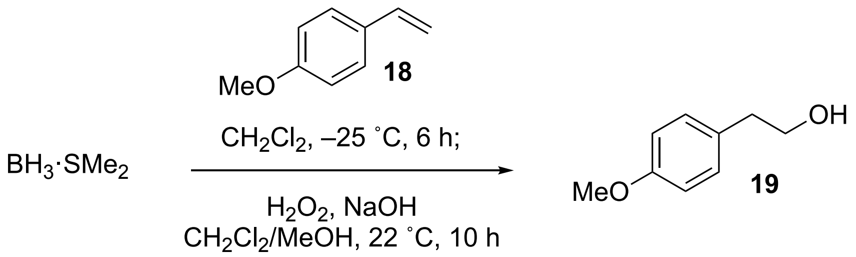

($^{13}C\{^1H\}$ NMR, 100 MHz, $CDCl_3$)

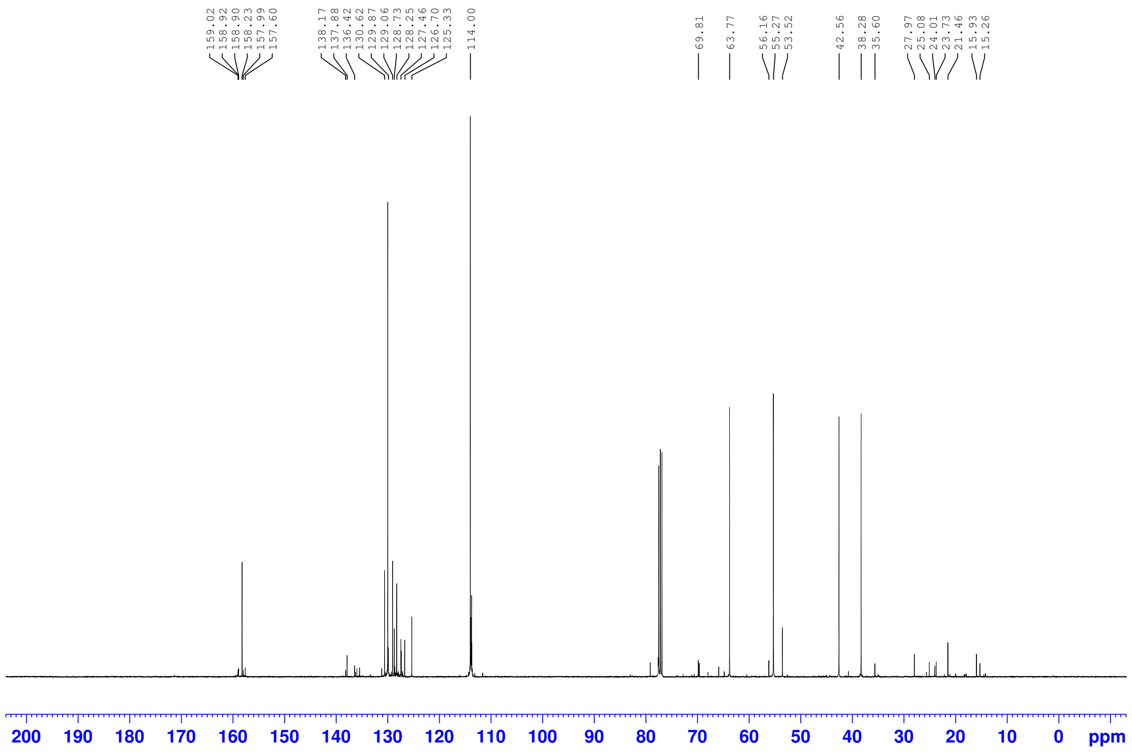

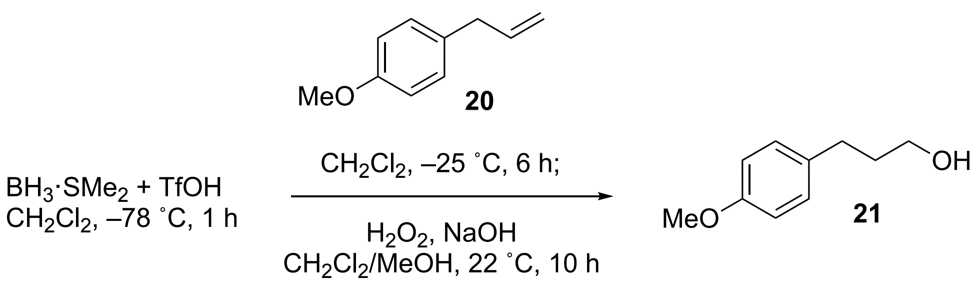

(Single-Pulse ^{1}H NMR, 400 MHz, $CDCl_3$)

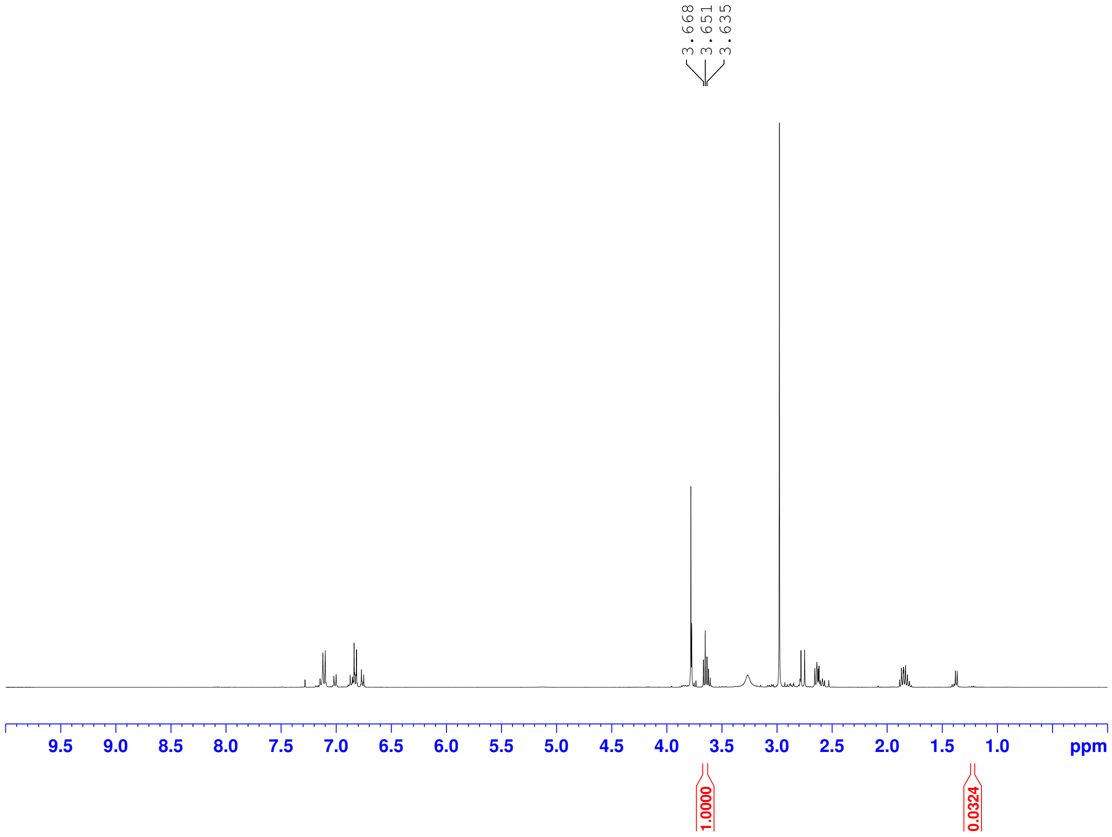

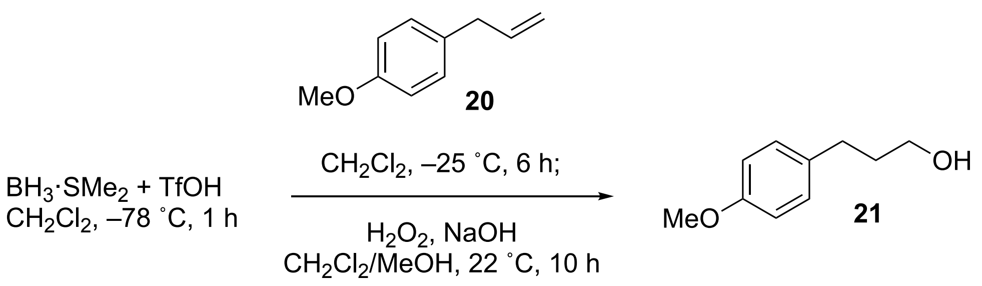

($^{13}C\{^{1}H\}$ NMR, 100 MHz, $CDCl_3$)

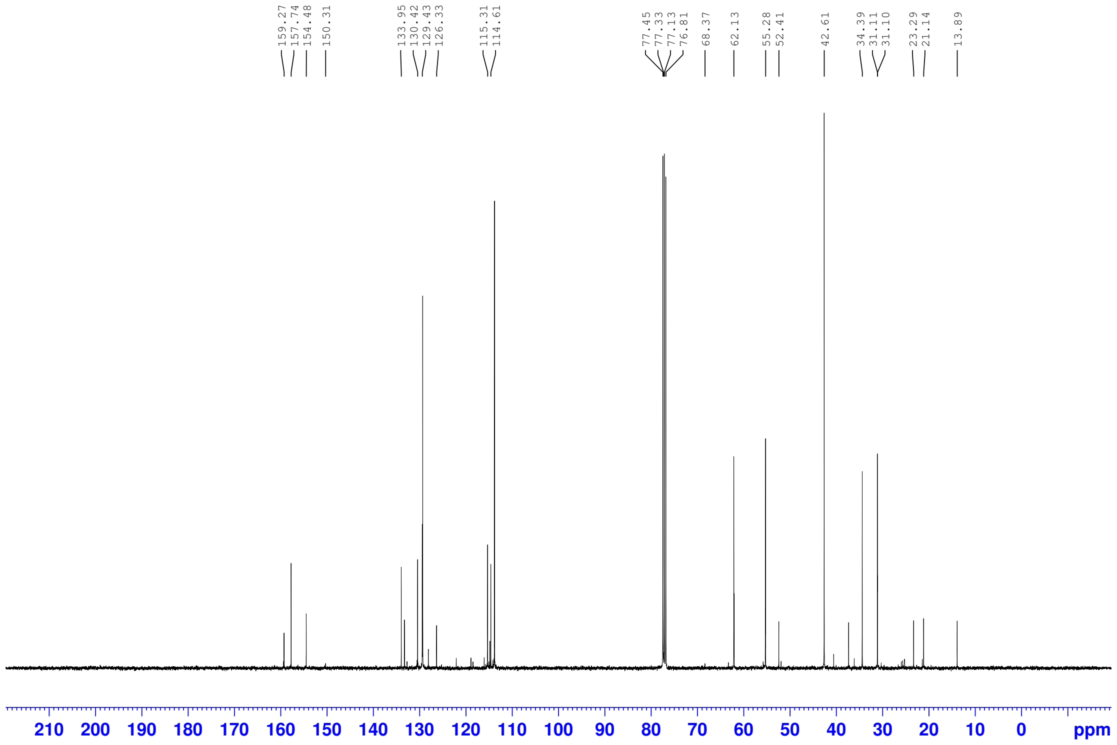

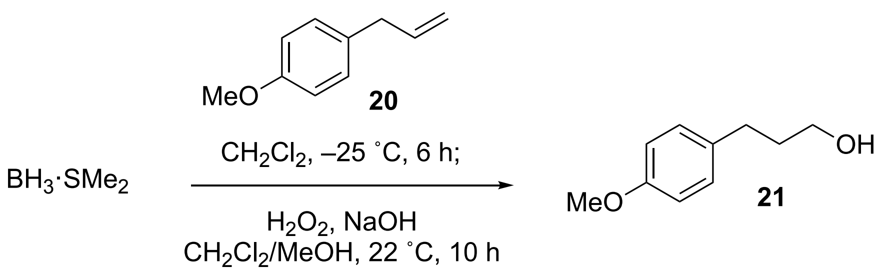

(Single-Pulse ^{1}H NMR, 400 MHz, $CDCl_3$)

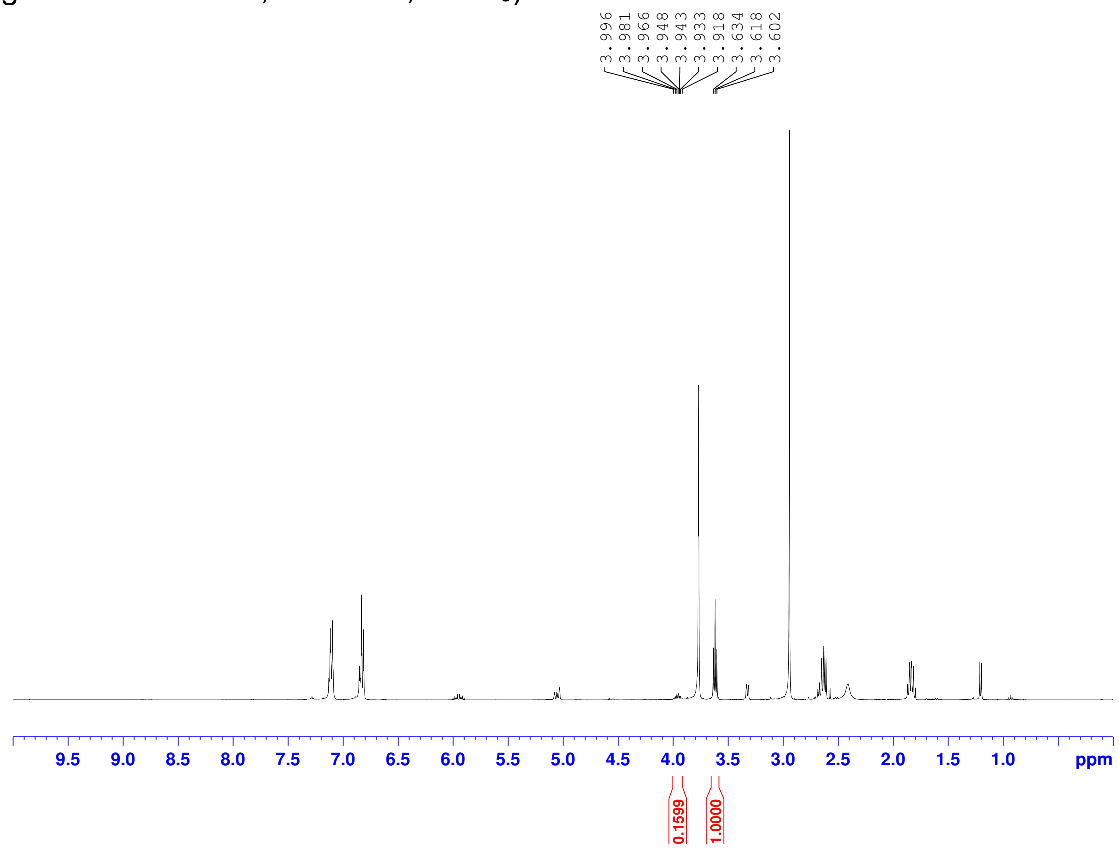

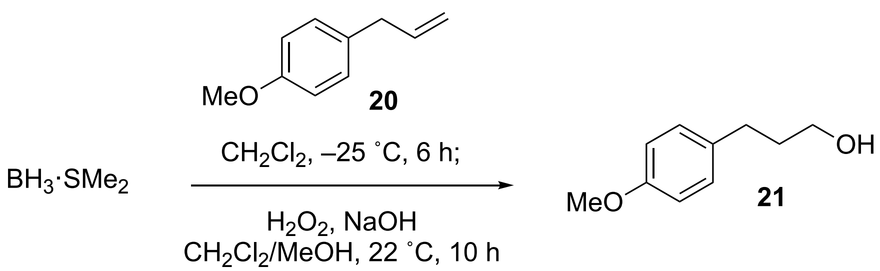

($^{13}C\{^{1}H\}$ NMR, 100 MHz, $CDCl_3$)

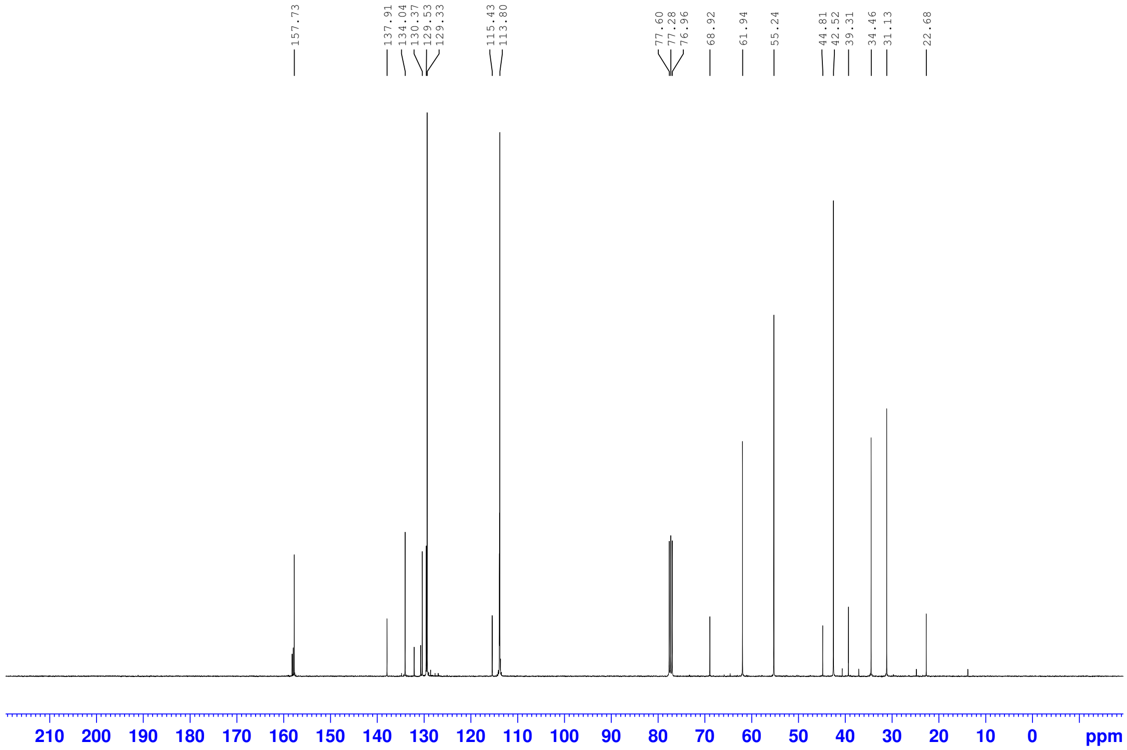

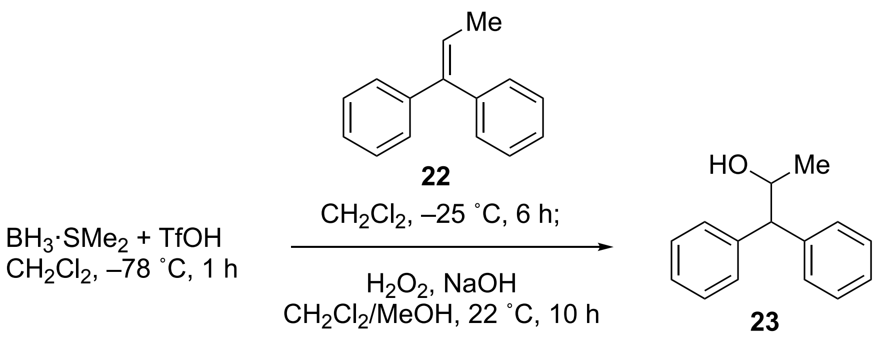

(Single-Pulse ^{1}H NMR, 400 MHz, $CDCl_3$)

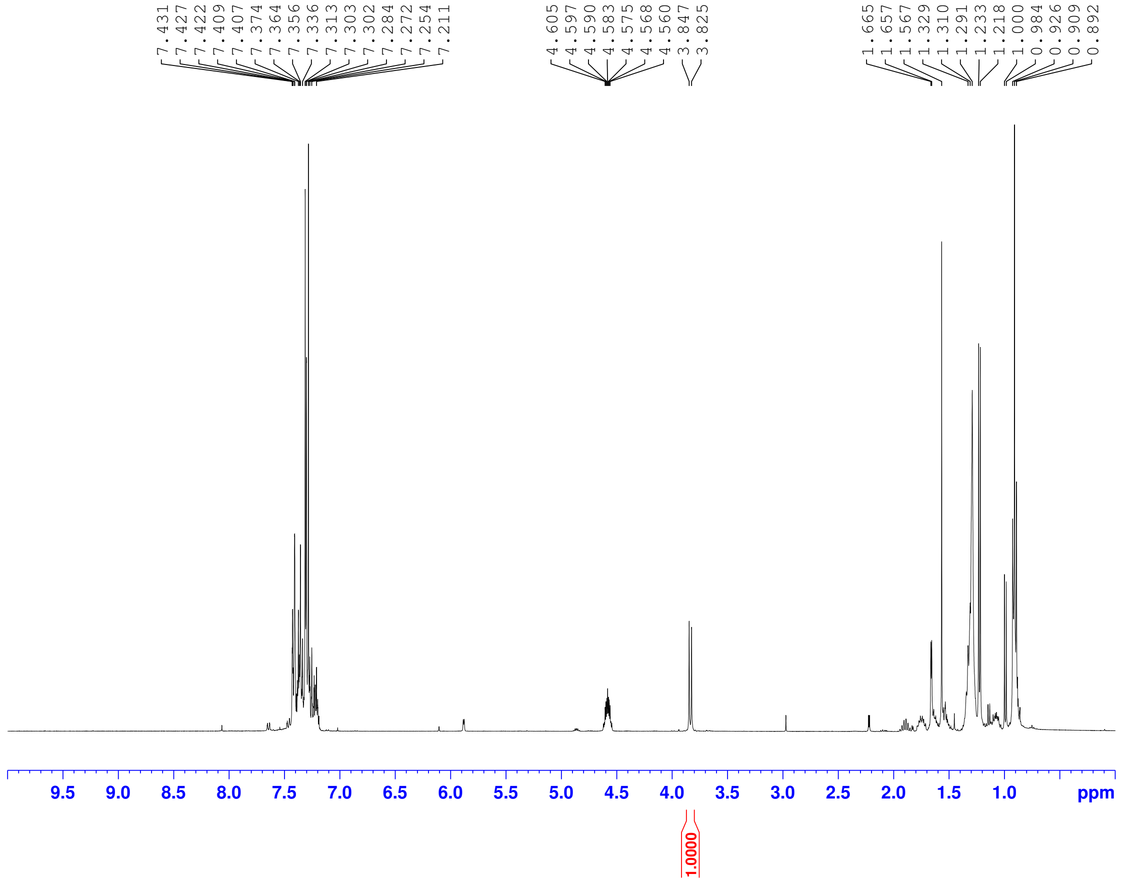

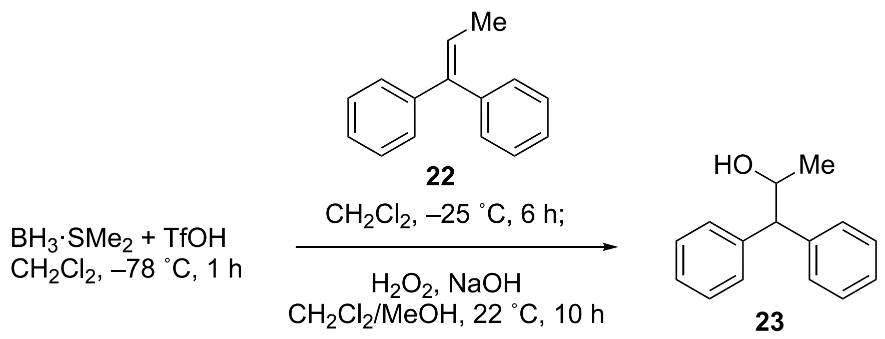

($^{13}C\{^1H\}$ NMR, 100 MHz, $CDCl_3$)

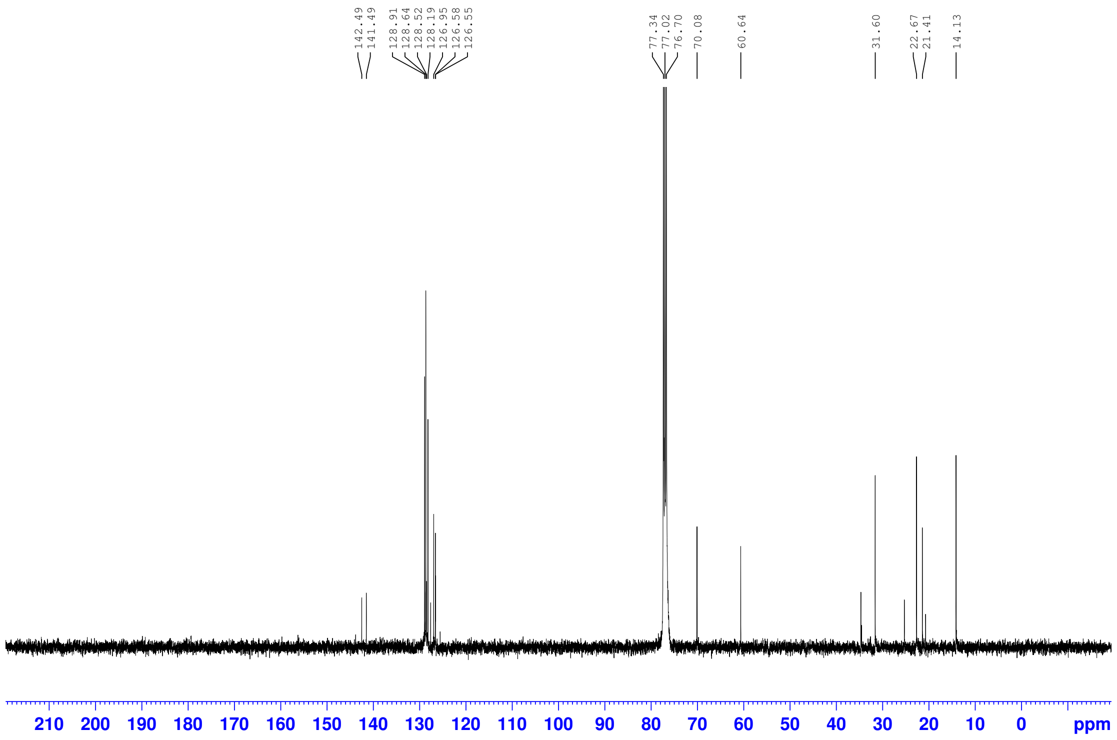

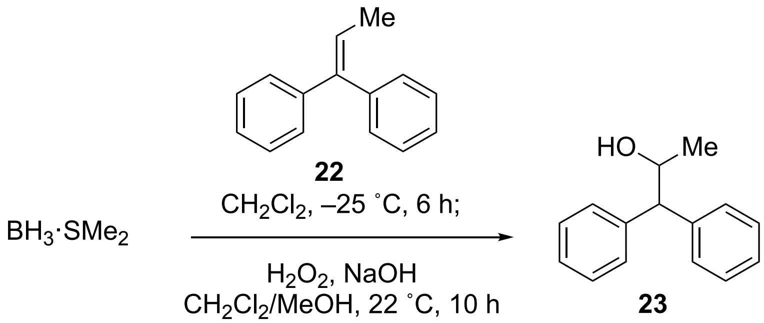

(Single-Pulse ^{1}H NMR, 400 MHz, $CDCl_3$)

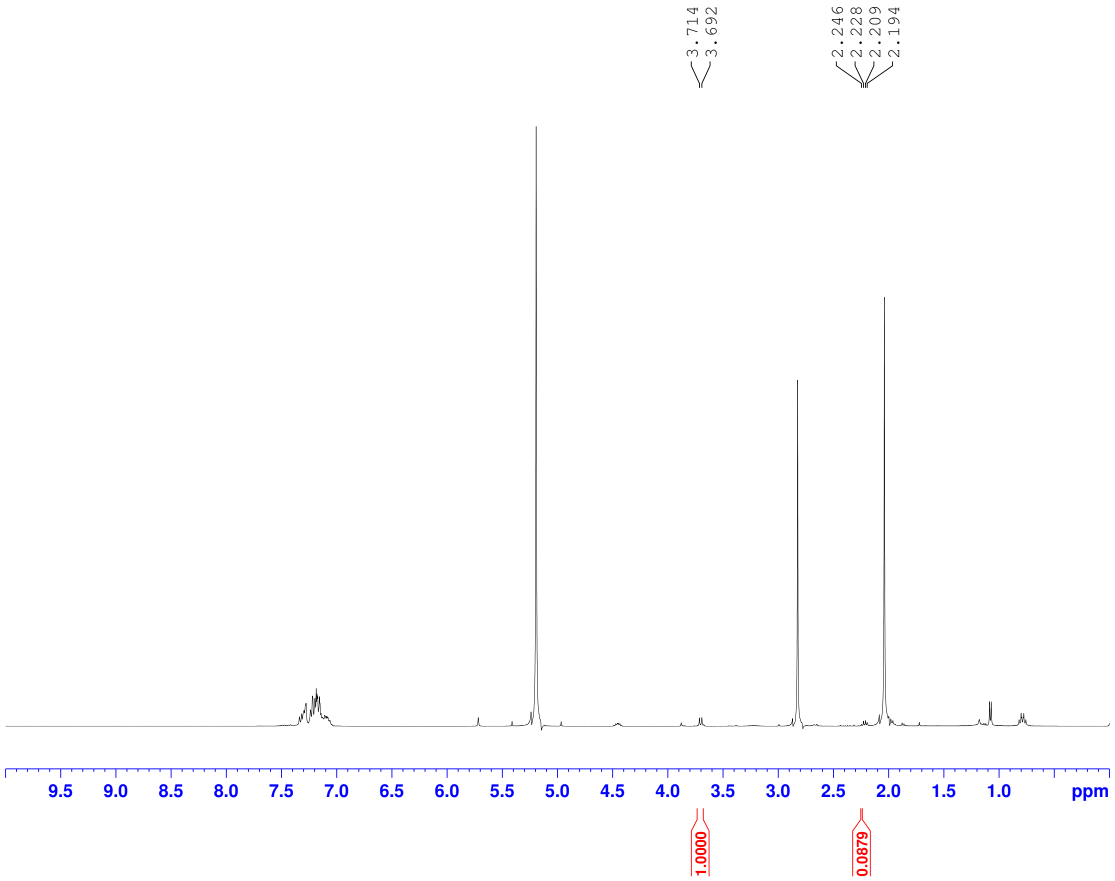

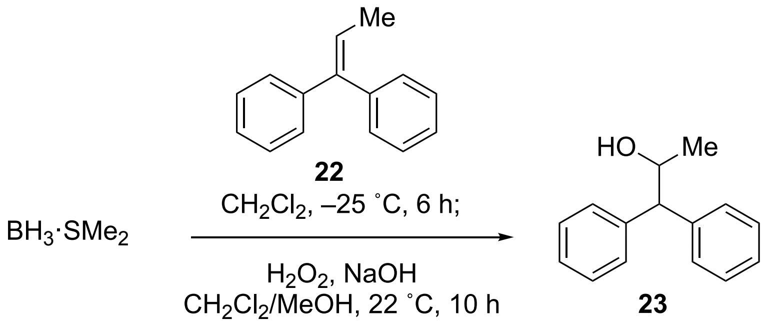

(^{13}C{^{1}H} NMR, 100 MHz, $CDCl_3$)

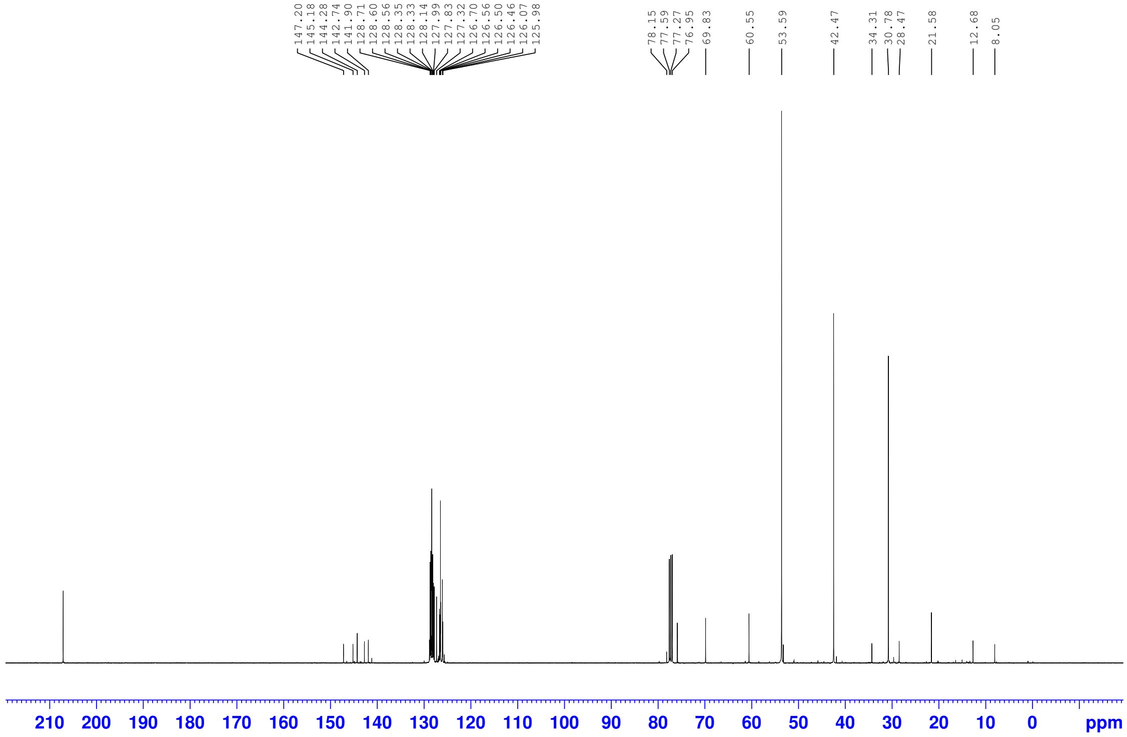

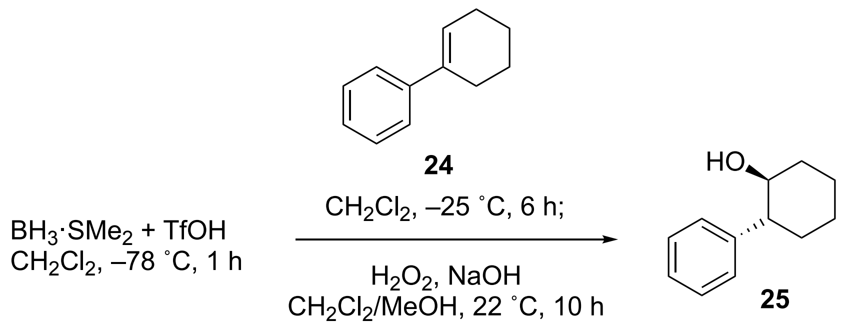

(Single-Pulse ^{1}H NMR, 400 MHz, $CDCl_3$)

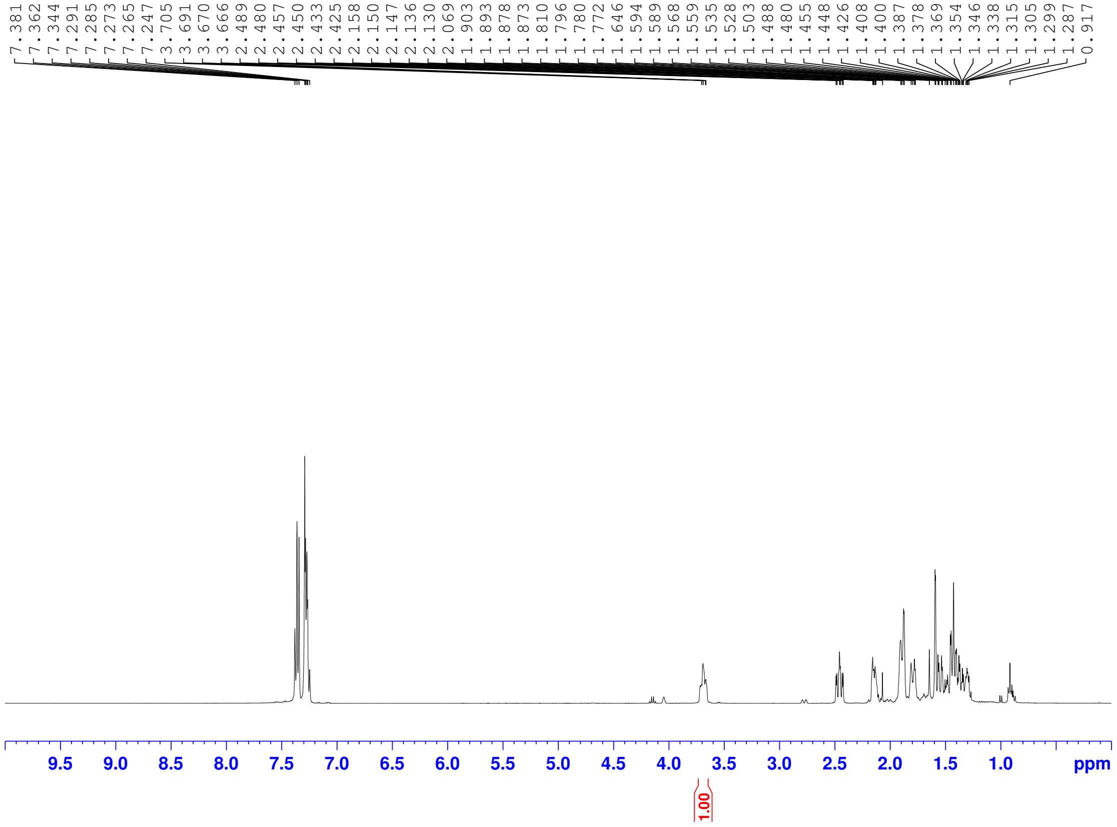

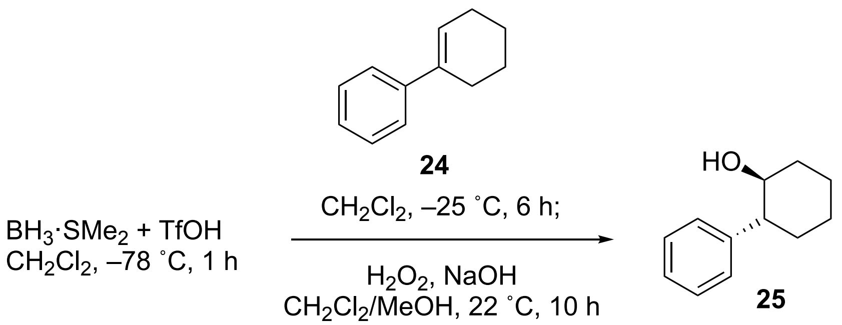

($^{13}C\{^{1}H\}$ NMR, 100 MHz, $CDCl_3$)

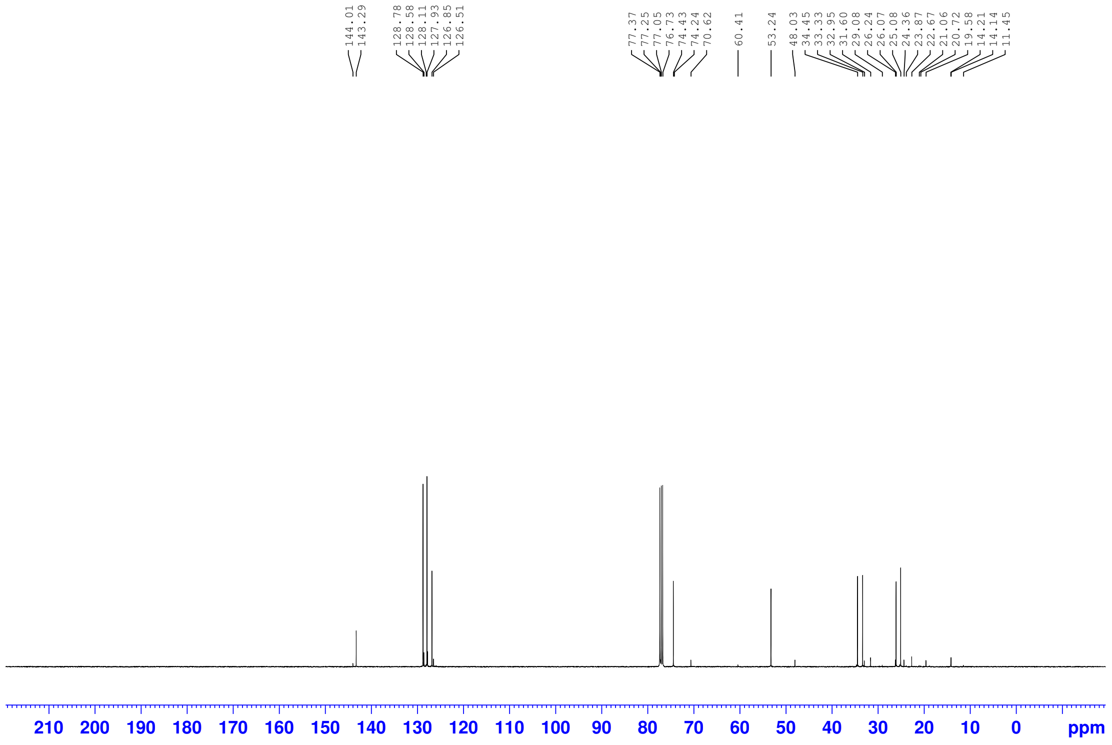

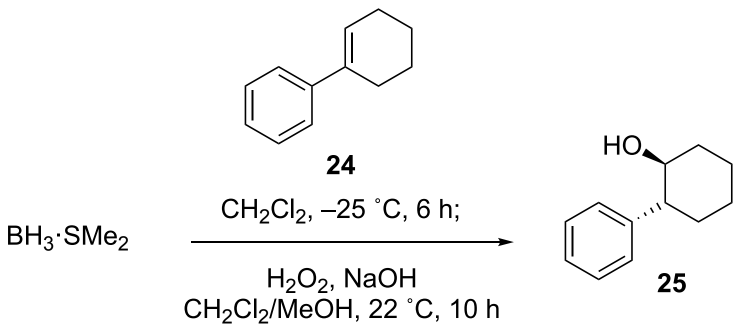

(Single-Pulse ^{1}H NMR, 400 MHz, CDCl$_3$)

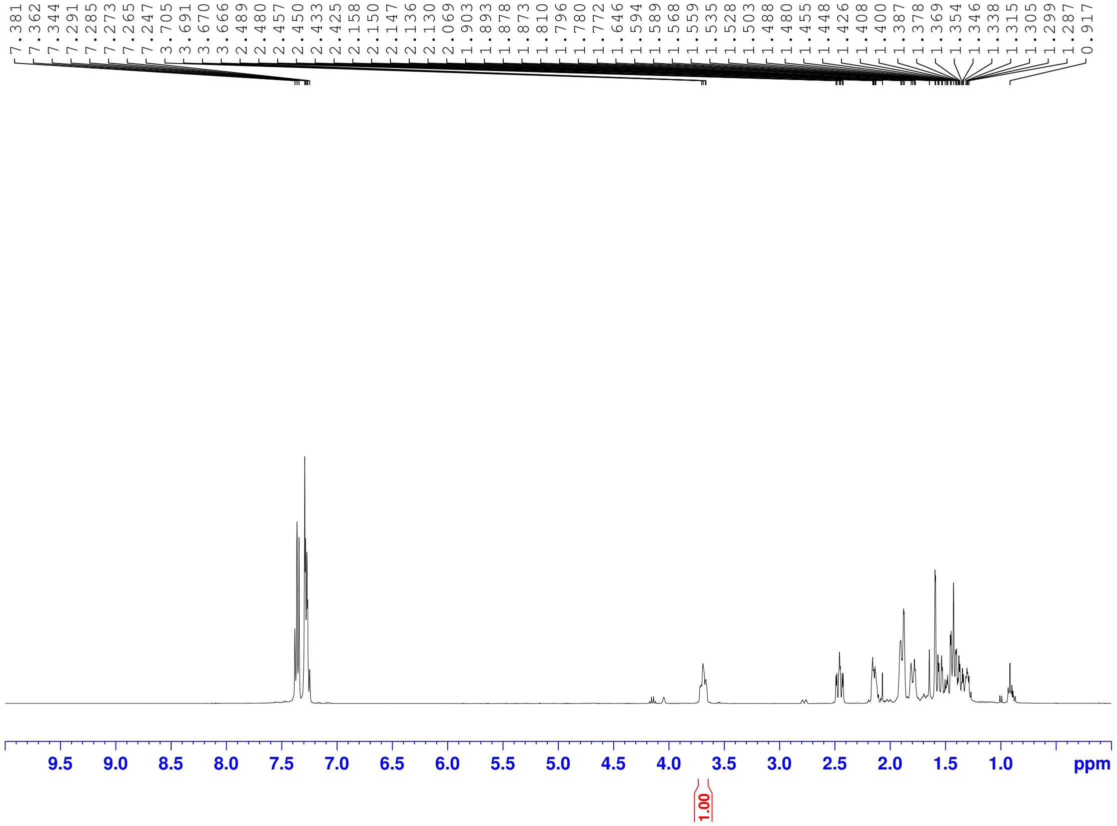

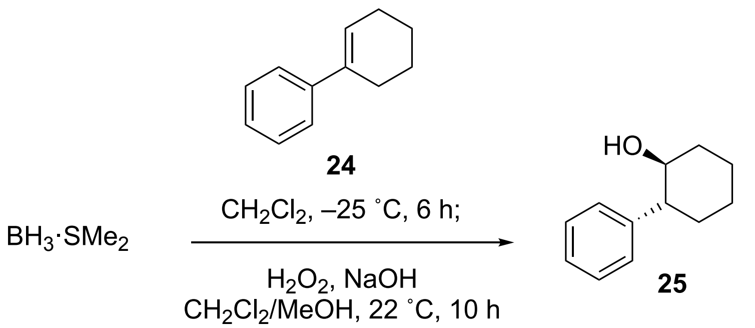

(^{13}C{^{1}H} NMR, 100 MHz, $CDCl_3$)

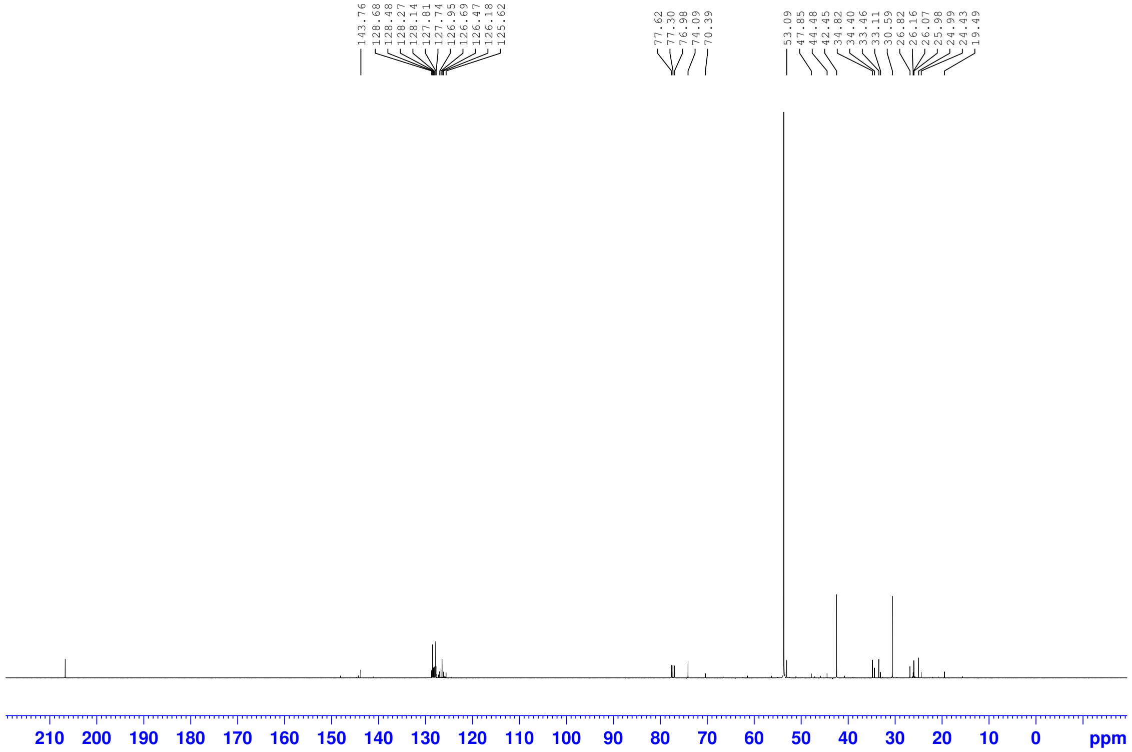

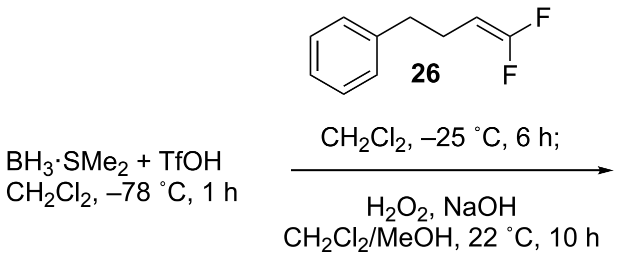

(Single-Pulse ^{1}H NMR, 400 MHz, $CDCl_3$)

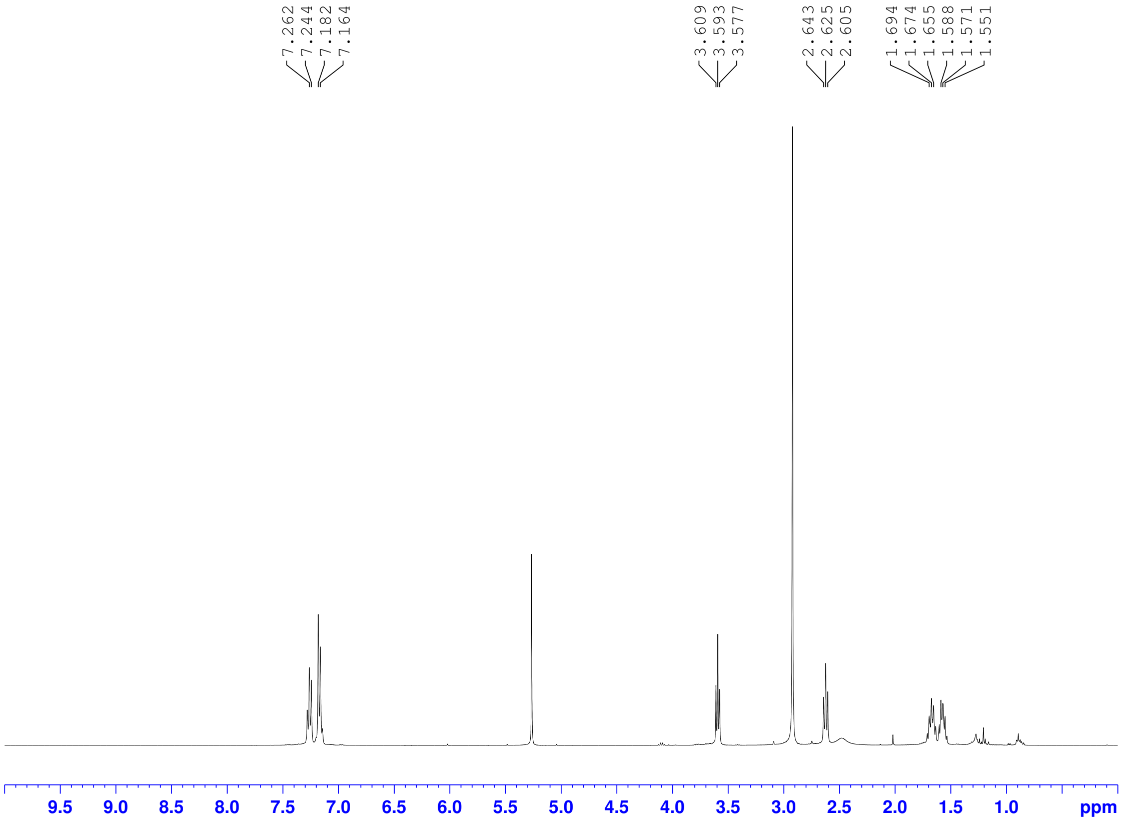

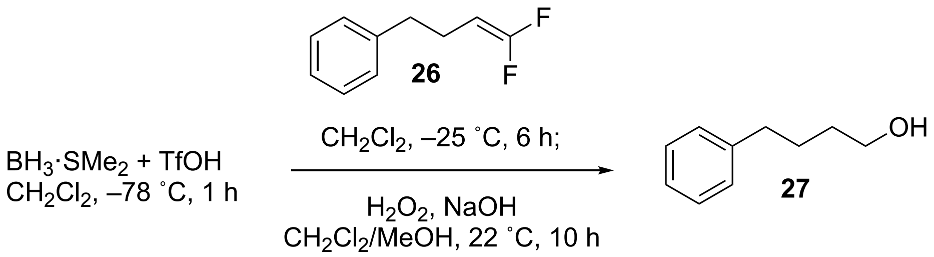

($^{13}C\{^{1}H\}$ NMR, 100 MHz, $CDCl_3$)

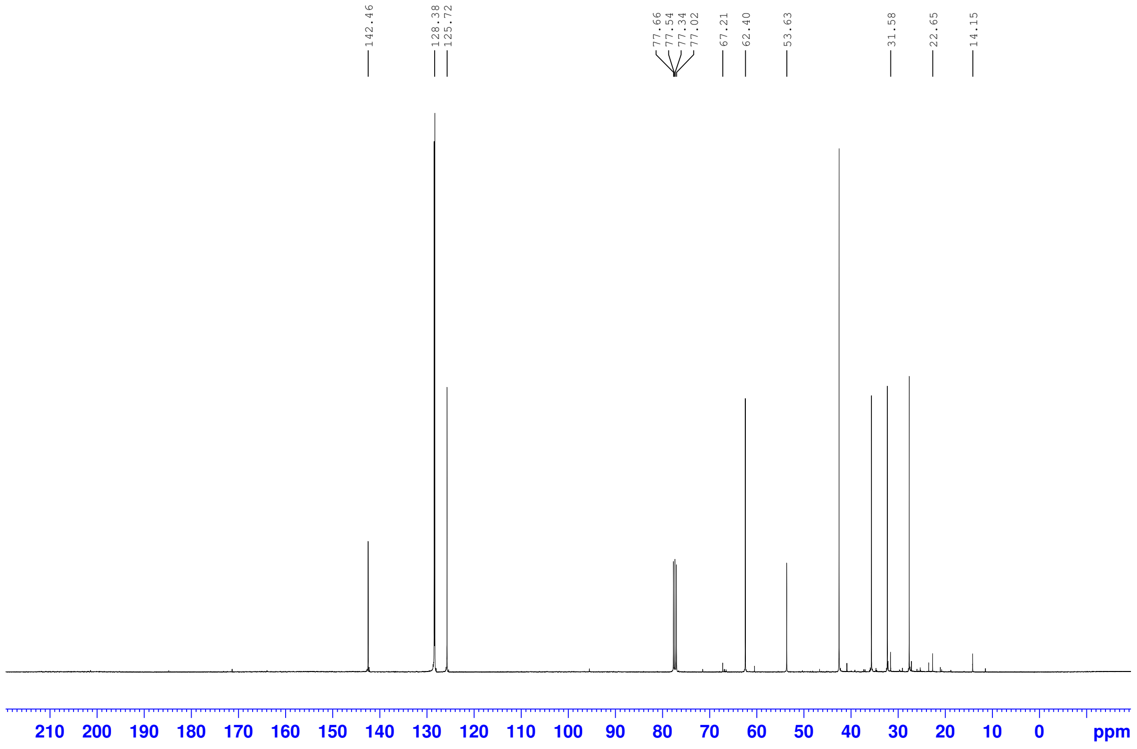

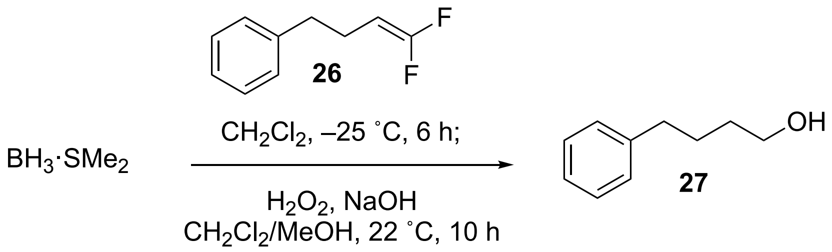

(Single-Pulse ^{1}H NMR, 400 MHz, $CDCl_3$)

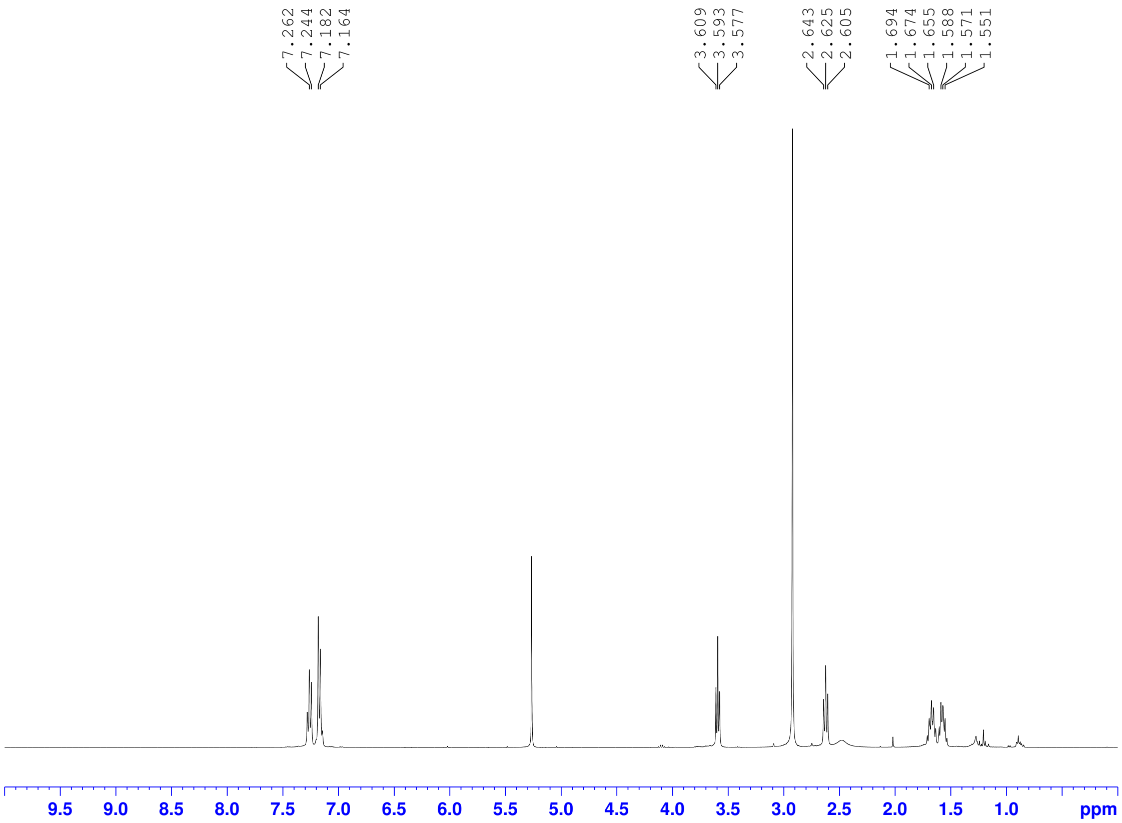

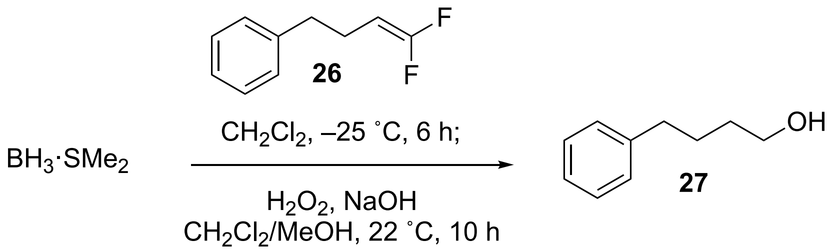

($^{13}C\{^1H\}$ NMR, 100 MHz, $CDCl_3$)

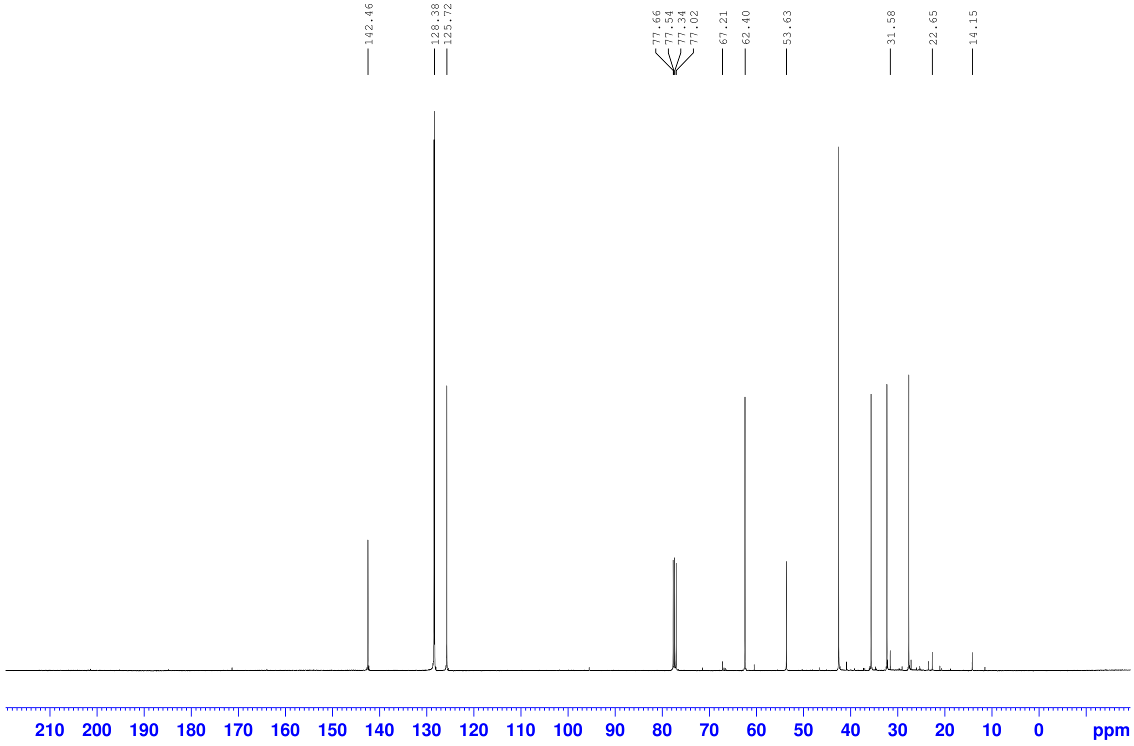

Supplement: Supplementary file 1 [file jo5c03231_si_001.pdf]
